# Parvovirus dark matter in the cloaca of wild birds

**DOI:** 10.1093/gigascience/giad001

**Published:** 2023-02-03

**Authors:** Ziyuan Dai, Haoning Wang, Haisheng Wu, Qing Zhang, Likai Ji, Xiaochun Wang, Quan Shen, Shixing Yang, Xiao Ma, Tongling Shan, Wen Zhang

**Affiliations:** Department of Laboratory Medicine, School of Medicine, Jiangsu University, Zhenjiang, Jiangsu 212013, China; Department of Clinical Laboratory, The Sixth Affiliated Hospital of Nantong University, Yancheng Third People's Hospital, Yancheng, Jiangsu 224001, China; School of Geography and Tourism, Harbin University, Harbin, Heilongjiang 150076, China; Department of Laboratory Medicine, School of Medicine, Jiangsu University, Zhenjiang, Jiangsu 212013, China; Qinghai Institute of Endemic Disease Prevention and Control, Xining, Qinghai 810099, China; Qinghai Institute of Endemic Disease Prevention and Control, Xining, Qinghai 810099, China; Department of Laboratory Medicine, School of Medicine, Jiangsu University, Zhenjiang, Jiangsu 212013, China; Department of Laboratory Medicine, School of Medicine, Jiangsu University, Zhenjiang, Jiangsu 212013, China; Department of Laboratory Medicine, School of Medicine, Jiangsu University, Zhenjiang, Jiangsu 212013, China; Department of Laboratory Medicine, School of Medicine, Jiangsu University, Zhenjiang, Jiangsu 212013, China; Qinghai Institute of Endemic Disease Prevention and Control, Xining, Qinghai 810099, China; Shanghai Veterinary Research Institute, Chinese Academy of Agricultural Sciences, Shanghai 810099, China; Department of Laboratory Medicine, School of Medicine, Jiangsu University, Zhenjiang, Jiangsu 212013, China

**Keywords:** metagenomic, *Parvoviridae*, wild bird, dark matter

## Abstract

With the development of viral metagenomics and next-generation sequencing technology, more and more novel parvoviruses have been identified in recent years, including even entirely new lineages. The *Parvoviridae* family includes a different group of viruses that can infect a wide variety of animals. In this study, systematic analysis was performed to identify the “dark matter” (datasets that cannot be easily attributed to known viruses) of parvoviruses and to explore their genetic diversity from wild birds’ cloacal swab samples. We have tentatively defined this parvovirus “dark matter” as a highly divergent lineage in the *Parvoviridae* family. All parvoviruses showed several characteristics, including 2 major protein-coding genes and similar genome lengths. Moreover, we observed that the novel parvo-like viruses share similar genome organizations to most viruses in *Parvoviridae* but could not clustered with the established subfamilies in phylogenetic analysis. We also found some new members associated with the *Bidnaviridae* family, which may be derived from parvovirus. This suggests that systematic analysis of domestic and wild animal samples is necessary to explore the genetic diversity of parvoviruses and to mine for more of this potential dark matter.

## Introduction

The ongoing pandemic of severe acute respiratory virus coronavirus 2 poses a serious threat to human health and has caused significant global economic loss. It has been suggested that the novel coronavirus originated in wild animals and infected humans through intermediate hosts such as bats and pangolins [1]. Many emerging infectious diseases in humans are caused by pathogens originating from a wide variety of animals [2] and are dominated by zoonoses (60.3%): the majority of these (71.8%) originate in wildlife [3] and have increased significantly over time [4, 5]. Animal-derived human pathogens have mainly arisen from warm-blooded vertebrates, mammals, and birds [6]. Birds’ unique adaptive immune system makes them a natural reservoir for viruses [7] and allows asymptomatic infection and virus coevolution to occur [8]. The destruction of wetlands, the hunting and killing of migratory birds, and increasing poultry consumption by humans have facilitated avian viruses to cross species barriers to other populations that subsequently may bring the viruses to new areas [9]. Birds may serve as vectors for disease vector transmission, as amplified hosts in the bird–vector–bird cycle, or as genetic sources for emerging cross-species viruses, including avian influenza viruses [10], West Nile virus [11], Sindbis virus [12], and Crimean-Congo hemorrhagic fever virus [13]. The transmission of viruses from birds to poultry production [10, 14] and then to humans therefore continues to be a threat to socioeconomic and public health [15] and may cause severe morbidity and mortality [16].

Parvoviruses are nonenveloped, round, icosahedral symmetry viruses with an approximately 4- to 6-kb-long single-stranded DNA genome. All parvoviruses have long inverted terminal repeats (LTRs) at both the 5′ and 3′ ends that can fold into hairpin-like structures related to expression and transcription strategies [17]. Their overall genomic structure is relatively conservative: a nonstructural (NS or Rep) open reading frame (ORF) and structural (VP or Cap) ORF about half the length of the genome, respectively [18]. The *Parvoviridae* family is divided into 3 subfamilies: the *Parvovirinae*, the *Densovirinae*, and the *Hamaparvovirinae*. The *Parvovirinae* is further subdivided into 10 genera: *Amdoparvovirus, Artiparvovirus, Aveparvovirus, Bocaparvovirus, Copiparvovirus, Dependoparvovirus, Erythroparvovirus, Loriparvovirus, Protoparvovirus*, and *Tetraparvovirus*. The *Densovirinae* comprise 11 genera: *Aquambidensovirus, Blattambidensovirus, Diciambidensovirus, Hemiambidensovirus, Iteradensovirus, Miniambidensovirus, Muscodensovirus,Pefuambidensovirus, Protoambidensovirus, Scindoambidensovirus*, and *Tetuambidensovirus*. The newly established subfamily *Hamaparvovirinae* comprises 5 genera: *Brevihamaparvovirus, Chaphamaparvovirus, Hepanhamaparvovirus, Ichthamaparvovirus*, and *Penstylhamaparvovirus* [19].

However, many parvovirus sequences cannot be accurately classified into a particular species or genus. According to the demarcation criteria of the International Committee for the Taxonomy of Viruses (ICTV), parvoviruses can be considered members of the same species if their NS1 proteins share >85% amino acid sequence identity. A genus can be identified as a group of species representing a single branch and shares at least 35–40% amino acid sequence identity with a coverage of >80% between any 2 members [19]. There have been reports of parvoviruses in various countries from very diverse hosts, including mammals such as humans [20], mice [21], canines [22], and chimpanzees [23]; arthropods such as crickets [24]; and birds such as ducks [25], red-crowned cranes [26], and pigeons [2].

Parvoviruses are often associated with the clinical signs of growth retardation and watery diarrhea in a wide range of animals and have been described in different species of birds [27]. In the early 1960s, goose parvovirus was identified in Europe, where it could cause a highly fatal disease of Muscovy ducklings and goslings called Derzsy's disease. In 1989, another type of parvovirus, with symptoms similar to those of goose parvovirus, was isolated from Muscovy ducks and was named Muscovy duck parvovirus. Both parvoviruses can cause substantial economic losses in waterfowl production and industry [28]. Recent surveys have shown widespread distribution of parvoviruses in wild birds and commercial chickens around the world, including China, South Korea, the United States, and European countries, including Hungary, Poland, and Croatia [29–32]. Due to the stability of the parvovirus in natural conditions, the virus can survive in feces or contaminated surfaces for up to a year, providing an additional source of infection to other animals [33]. The maternally derived specific antibodies to parvovirus have been detected in hatching eggs and newly hatched ducklings, confirming the possibility of a potential vertical transmission of the virus [34].

With the development of next-generation sequencing (NGS) technology, more and more viral pathogens have been detected. However, most of the detected viral sequence (usually 60–95%) [35] cannot be aligned to any reference viral sequences [36], which are referred to as “viral dark matter” and may contain potential zoonotic pathogens. Therefore, mining “viral dark matter” can not only help us fill the “gap” in the evolutionary relationship between viruses and expand the range of known viral hosts but also help us predict and monitor the prevalence of viral diseases that may occur in the future. Using viral metagenomics, we analyzed the composition and distribution of divergent parvovirus in the intestinal tract of 3,404 wild birds so as to further explore the parvovirus “dark matter” to enrich known virus libraries and to explore their potential public health significance.

## Materials and Methods

### Sample collection and preparation

A total of 3,404 cloacal swabs of wild and breeding bird specimens were collected for a previously published virome study from 5 different provinces in China (Supplementary Fig. S2) from 2018 to 2019 [37]. All specimens were shipped on dry ice. Cloacal swabs specimens were resuspended individually in 0.5 mL phosphate-buffered saline and vortexed at 1,800 rpm for 5 minutes and centrifugated for 10 minutes at 15,000 × *g*; the supernatant was then collected in a microcentrifuge tube and stored at −80°C. About 0.1 mL supernatant of each cloacal swab specimen from the same bird species was added to sample pools (Supplementary Table S1). Subsequently, the supernatant was filtered through a 0.45-µm filter (Millipore, Darmstadt, Germany) to remove eukaryotic, giant viruses and bacterial cell-sized particles [38].

Ethical approvals were given by the Ethics Committee of Key Laboratory of Wildlife Diseases and Biosecurity Management of Heilongjiang Province (reference number WDBM2018-023), the Ethics Committee of Jiangsu University (reference number 2018ujs18023), and the Ethics Committee of Chinese Academy of Agricultural Sciences (reference number SVRI2017091). Sample collecting was performed in accordance with the Wildlife Protection Law of the People's Republic of China. All samples were shipped to the Shanghai Veterinary Research Institute of Chinese Academy of Agricultural Sciences, where sample preparations were conducted in a biosafety level 2 laboratory.

### Viral metagenomic analysis

The filtrates enriched in viral particles were then treated with a cocktail of DNase, RNase, benzonase, and Baseline-ZERO to digest unprotected nucleic acid at 37°C for 90 minutes [39]. Total nucleic acids were then extracted using the QIAamp MinElute Virus Spin Kit (Qiagen, Venlo, Netherlands ) according to the manufacturer's protocol. A total of 238 libraries were then constructed using a Nextera XT DNA Sample Preparation Kit (Illumina, San Diego, USA) and sequenced using the Illumina MiSeq platform (RRID:SCR_016379) with 250 bp ends with dual barcoding for each individual sample or sample pool. The information about each library is shown in Supplementary Table S1. For bioinformatics analysis, pair-end reads of 250 bp generated by Miseq were debarcoded using vendor software from Illumina. An in-house analysis pipeline running on a 32-node Linux cluster was used to process the data. Clonal reads were removed, and low-sequencing-quality tails were trimmed using a Phred quality score of 10 as the threshold. Adapters were trimmed using the default parameters of VecScreen (NCBI BLASTn) with specialized parameters designed for adapter removal. The cleaned reads were *de novo* assembled within each barcode using the ENSEMBLE assembler [40]. Contigs and unassembled reads were then matched against a customized viral proteome database using BLASTx with an E-value cutoff of <10^−5^, where the virus BLASTx database was compiled using NCBI virus reference proteome (ftp://ftp.ncbi.nih.gov/refseq/release/viral/) to which viral proteins sequences were added from the NCBI nr fasta file (based on annotation taxonomy in the Virus Kingdom). Candidate viral hits were then compared to an in-house nonvirus, nonredundant (NVNR) protein database to remove false-positive viral hits, where the NVNR database was compiled using nonviral protein sequences extracted from the NCBI nr fasta file (based on annotation taxonomy excluding the Virus Kingdom). Contigs without significant BLASTx similarity to the viral proteome database were searched against viral protein families in the vFam database [41] using HMMER3 (RRID:SCR_005305) [42–44] to detect remote viral protein similarities [42–44].

### Analysis of the sequence

For assembly of the parvovirus genomes, the contigs showing significant BLASTx similarity to parvoviruses were selected [45]. The contigs with consensus sequence length >500 bp were subjected to further analysis, where the individual contig was used as reference for mapping to the raw data of its original barcode using the Low Sensitivity/Fastest parameter in Geneious (RRID:SCR_010519) [45]. Those prolonged contigs that had the major nonstructural protein and structural protein, as well as some contigs that had only a nonstructural protein, were included in this study. The contigs that had only a putative nonstructural protein are not shown in this study. Splice sites were also detected using Neural Network of the Berkeley Drosophila Genome Project. The search for protein homologies was made by BLAST programs at the NCBI website (http://www.ncbi.nlm.nih.gov/Blast.cgi) against the nonredundant protein database, and alignment of protein sequences was performed using the Mega 10.2.2 (RRID:SCR_000667).

### Phylogenetic analysis

To investigate the evolutionary relationship of bird fecal parvovirus to other members of the family *Parvoviridae*, translated sequences from the coding region NS of wild bird fecal parvoviruses and reference sequences in GenBank were aligned using MUSCLE in MEGA v10.2.2 with default settings. Bayesian inference trees were then constructed using MrBayes v3.2 (RRID:SCR_012067) [46]. During MrBayes analysis, we set “prset aamodelpr = mixed” for the phylogenetic analysis using amino acid sequences, which allows the program to utilize the 10 built-in amino acid models. The Markov chain was run for a maximum of 1 million generations, in which every 50 generations were sampled and the first 25% of Markov chain Monte Carlo samples were discarded as burn-in.

## Results

### Overview of the virome

**Figure 1:**
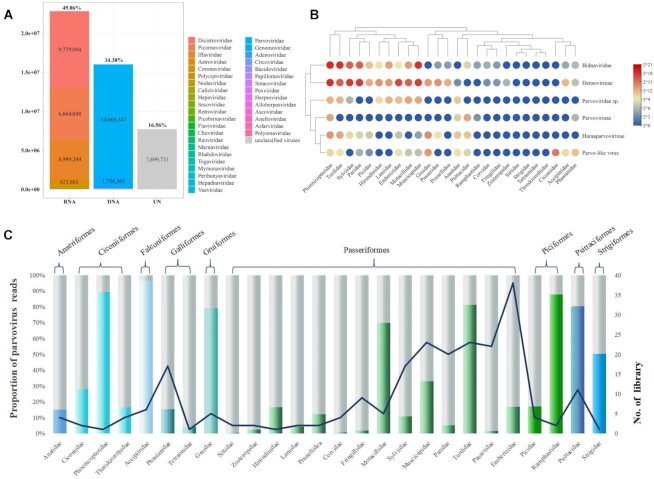
Overview of virome. (A) Composition of each virus family in bird cloacal sample. (B) The shade of color in each circle shows the abundance of each group virus in each family of birds. The 170 viruses identified in this study were tentatively grouped into 6 groups. (C) Information of bird species and library. Colored bars show the proportion of parvovirus reads of all viruses. The broken line shows the number of libraries for each family of birds.

This study included 3,404 wild bird cloacal swab specimens belonging to 26 different families of birds. The 3,404 samples were combined into 228 pools for viral metagenomic analysis, each of the pools being of the same species (Supplementary Table S1). After Illumina sequencing, a total of 46,494,515 reads showing similarity to viruses were obtained, accounting for 9.62% of the total reads. The cellular organisms (archaea, bacteria, and eukaryotes) and other non-virion-associated reads were removed. As shown in Fig. 1 A, the proportion of RNA virus reads and DNA virus reads was 49.06% and 34.38%, respectively. There were about 36 families of viruses in the gut of the wild birds, the highest of which was parvovirus, with 14,068,347 *Parvoviridae*-associated reads, maxing approximately 30.26% of the total virus reads.

The sequence reads of the family *Parvoviridae* were selected for further analysis. The singlets and the *de novo* assembled contigs of 228 pools were compared to the GenBank nucleotide database using BLASTn to remove those showing significant similarity to known virus, and 170 viral contigs (1.4 to 7.0 kb in length) were obtained (Supplementary Table S2). Seventy of the 170 contigs were mostly related to densovirus belonging to clades infecting arthropods. In addition, we obtained 2 *Dependoparvovirus* contigs and 3 *Aveparvovirus*contigs of the *Parvovirinae* subfamily, which can infect vertebrate hosts. There were 31 contigs of Parvo-like hybrid virus and 28 contigs belonging to the novel subfamily *Hamaparvovirinae*, and 17 contigs could not be assigned to an existing subfamily. Interestingly, we found 19 uncommon contigs that may be new members of different genera within the *Bidnaviridae* family, which are thought to have evolutionarily derived from a parvovirus ancestor [47]. The mapping analysis using the 170 genomes against the 228 NGS data revealed the virus distribution in the 228 sample pools, where the distribution patterns were further analyzed based on birds’ families (Fig. 1B) and sampling sites (Supplementary Fig. S3). Most *Densovirnae, Bidnaviridae*, and unclassified *Parvoviridae* sequences shared between samples were related to passeriformes, while *Parvovirinae* sequences were less likely to be shared. In addition, many parvoviruses were found among different birds at MaoEr mountain (MES), such as MW046591, MW046463, and MW046598, which were grouped as unclassified *Parvoviridae*. Some highly similar viruses were also found in birds from different regions, such as the strains fcc107par07 (MW046633) and wiw119par01 (MW046604), both belonging to the subfamily *Densovirinae*, were found in birds from Jilin and Heilongjiang provinces (Supplementary Fig. S3).

### Identification of novel viruses of the subfamily *Parvovirinae*

Among the 10 genera of the *Parvovirinae* subfamily, 5 virus genomes from *Aveparvovirus* (*n* = 3) and *Dependoparvovirus* (*n* = 2) genera were found in the cloaca of birds, and these 2 genera are known avian parvoviruses. The poultry parvovirus, first identified in the early 1980s and later assigned to the genus *Aveparvovirus*, has been found worldwide in the intestines of young and healthy birds with intestinal syndrome [35]. The *Dependoparvovirus*, or adeno-associated virus as it was originally known, is helper dependent and requires coinfection with a helper virus (herpesvirus or adenovirus) for productive infection.

**Figure 2:**
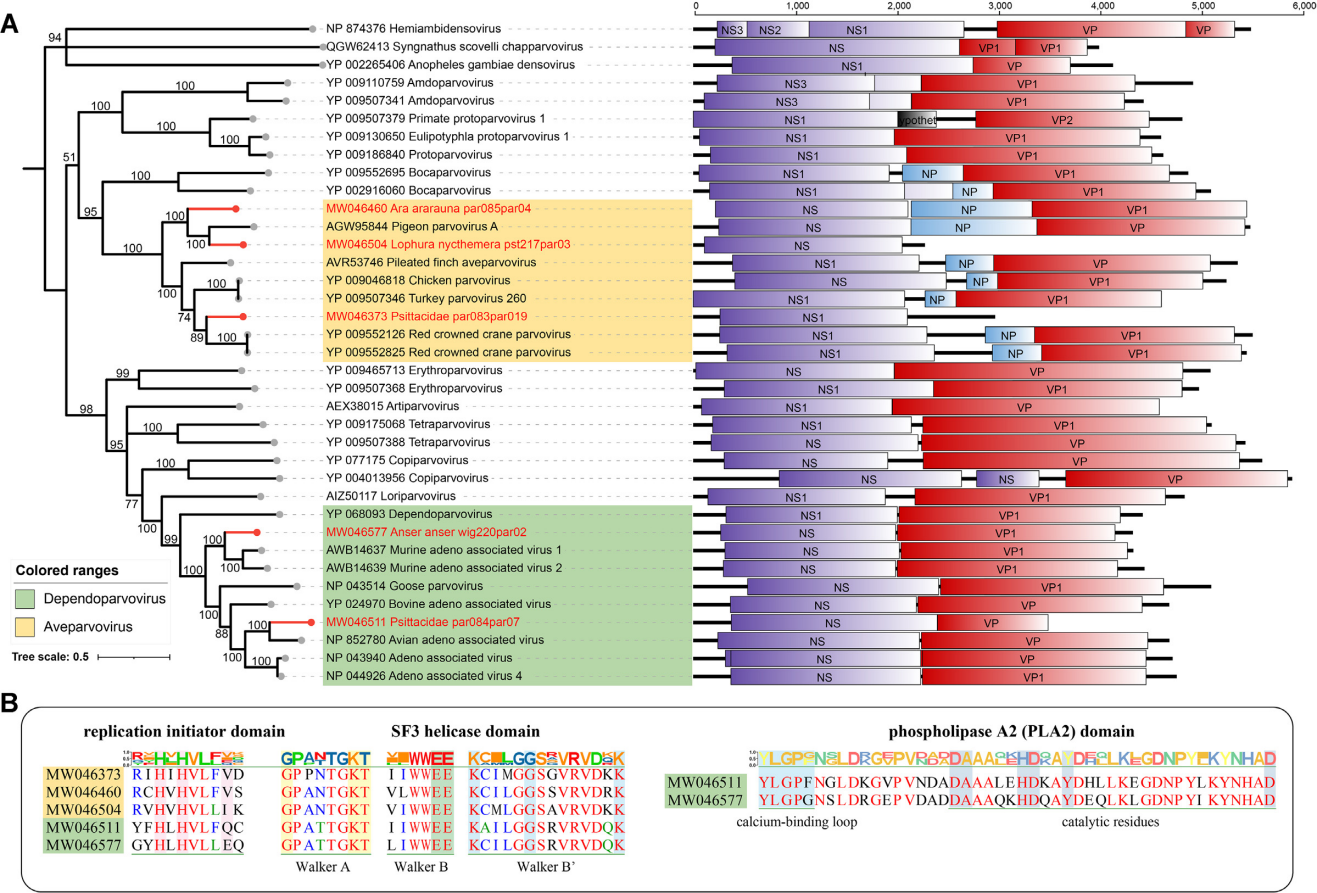
Identification of novel viruses of the subfamily *Parvovirinae*. (A) Bayesian inference trees were constructed using MrBayes v3.2 based on amino acid sequences of NS1 of parvovirus; within trees, the viruses found in this study are labeled in red. Scale bar indicates the amino acid substitutions per site. Genome organization of each parvovirus is indicated. Purple rectangles: putative NS; red: putative VP; blue: putative NP. (B) Identification of the replication initiator domain and SF3 helicase domain in the NS1 protein and the PLA2 domain in the N-terminal portion of the VP1 protein.

Sequence analysis of the 2 nearly complete genomes (MW046460 and MW046577) showed typical genomic size and organization that contained 2 major ORFs (Fig. 2A). The ORF located on the left side of the viral genome encodes the nonstructural protein of around 600 amino acids (aa). The ORF on the right side of the viral genome encodes about a 700-aa capsid protein. As shown in Fig. 2B, several conserved domains were identified, including a replication initiator domain (xxHxHxxxxx) and an SF3 helicase domain with an ATP- or GTP-binding Walker A loop (GxxxxGKT), Walker B loop (xxxxEE), and Walker B′ loop (KxxxxGxxxxxxxK). In contrast to *Dependoparvovirus, Aveparvovirus* does not contain the phospholipase A2 (PLA2) sequence motif, the VP1-unique region. Furthermore, a putative nucleoprotein (NP) was identified, in the middle of 2 major ORFs.

Phylogenetic analysis based on the complete NS1 amino acid sequences showed that these avian parvoviruses grouped into 4 different clades in the genera *Aveparvovirus* and *Dependoparvovirus* (Fig. 2). Combined with the results of BLASTp, the similarity of 5 NS1 proteins with their most closely related viruses is all less than 65%, lower than the demarcation criteria of 85%, suggesting that these viruses are new species.

### Identification of novel viruses of the subfamily *Denovirinae*

*Densovirinae* have in common the capacity of causing morphologic “dense cores” (nuclei forming large cuboidal or circular inclusions). The entire viral subfamily is named densonucleosisviruses, “densoviruses” for short, because of this pathologic feature [48]. In recent years, the unexpected diversity of densovirus, along with the rapid development of high-throughput sequencing and viral metagenomics, has revealed how little we know about their biological characteristics and evolutionary history.

**Figure 3:**
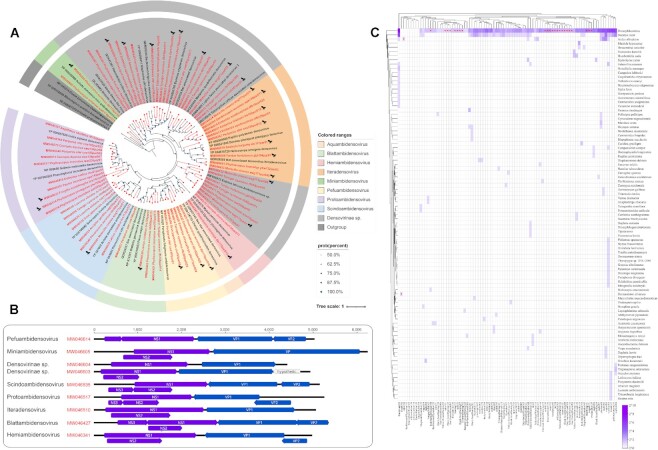
Identification of novel viruses of the subfamily *Densovirinae*. (A) Bayesian inference trees were constructed using MrBayes v3.2, respectively based on amino acid sequences of NS1 of densovirus; within trees, the viruses found in this study are labeled in red. Scale bar indicates the amino acid substitutions per site. Potential viral hosts are shown in black silhouette. (B) Genome organization of each genus is indicated. Purple arrows and rectangles: putative NS1; blue: putative VP. (C) The composition of arthropod species in the densovirus-positive pools. The horizontal ordinate represents different pools, while the longitudinal axis represents the arthropod species. The shade of the color represents the number of the sequence reads. The library that detected only 1 arthropod species is marked with a red star.

We identified 70 densovirus genomes in this study. Most of them were rather divergent from all other densoviruses with an aa identity of 49–65%, except strains wag171par017 (MW046541), coa196par03 (MW046427), stc111par01 (MW046510), and gbt104par01 (MW046508), which shared >85% sequence similarity. As shown in Fig. 3A, the phylogenetic tree based on NS1 protein showed that the first 44 densoviruses we identified clustered with members of 7 genera of the subfamily *Densovirinae*, while the remaining 26 novel densoviruses (*Densovirinae* sp.) formed new clades that could not cluster with previously established genera. The NS1 gene is the most conserved of parvovirus gene sequences, while the VP genes are much more diverse. It is therefore reasonable to speculate that the phylogenetic trees of these 2 genes may have some differences in their topological structure. Even so, the phylogenetic tree shows a similar topology across the board. Specifically, densoviruses have 2 main genomic structures: the monosense genome, which mainly includes the genus *Iteradensovirus*, and the ambisense genome, which mostly includes the remaining 7 genera and unclassified densovirus. Just like iteradensoviruses described previously, 20 novel monosense genomes contained 3 intronless genes with essentially identical positions but slightly different sizes. The largest, ORF1, had a coding capacity of 566–753 aa and the typical nucleoside triphosphatase motif for NS1. ORF2, with the PLA2 motif typical for VP1, had a coding capacity of 590–716 aa. ORF3 corresponded to NS2, with a 253–466 aa coding capacity, and typically overlapped the N-terminus of NS1. The novel densovirus genomes had an ambisens genome organization of 4,514–6,256 nucleotide long. In the clade of ambidensovirus, 40 novel densovirus genomes were exceptionally compact in size, including unusually small NS proteins and a predicted major capsid protein (Fig. 3B). The NS cassette consisted of 3 genes on 1 strand, while a single gene or 2 genes encoded the structural proteins on the complementary strand. Interestingly, the PLA2 motif was absent in VP1 but found in the N-terminal region of VP2. It is possible that the leak-scanning mechanism divided VP transcripts into VP1 and VP2.

Densoviruses are generally hosted by invertebrates [49], and to study the host assignments of these novel densoviruses, we included densovirus-positive libraries in this study for further analysis. We compared the sequences of densovirus-positive libraries against the total mitochondrial proteome database downloaded from GenBank. The results are displayed as a heatmap, which indicates the composition of invertebrate species present in the samples. There were 23 pools that contained only mitochondrial sequences from a single species of *Drosophila erecta*. Our data indicated that the potential hosts of these 26 novel densoviruses in this study could be fruit flies (*D. erecta*) (Fig. 3A, C).

### Identification of novel viruses of the subfamily *Hamaparvovirinae*

In the past few years, a type of divergent parvovirus has been identified in a broad range of host species, including wild rats, mice, domestic turkeys, fish, and dogs [21, 50–53]. This divergent lineage was described under an unofficial umbrella term “Chapparvovirus” and grouped in unclassified Parvovirinae. In 2019, they were reclassified by the ICTV and placed in the genus *Chaphamaparvovirus* of the newly proposed subfamily *Hamaparvovirinae* [19]. The name “Hama” means “together” in Greek, reflecting the fact that their natural hosts infect both vertebrates and invertebrates.

**Figure 4:**
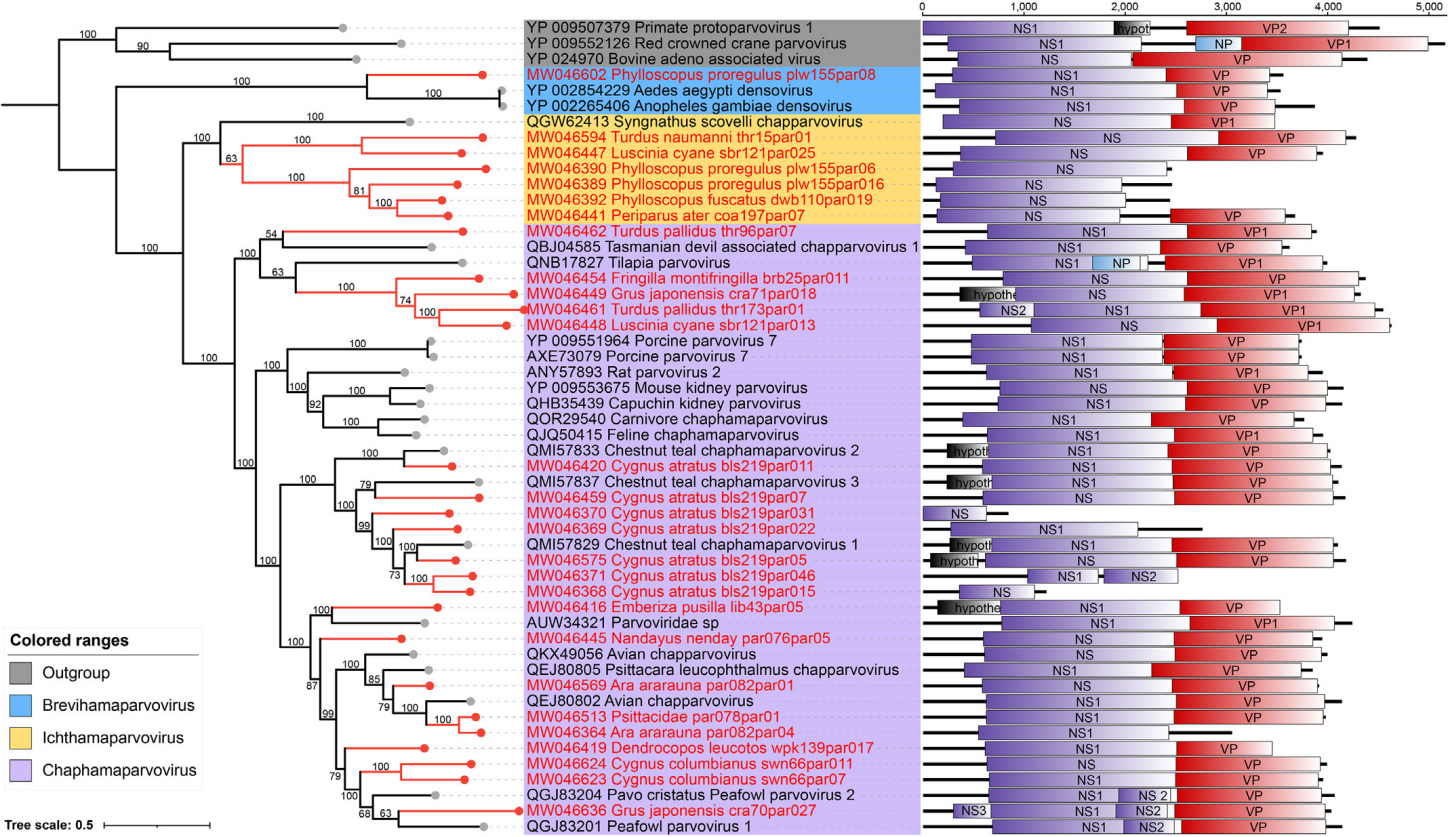
Identification of novel viruses of the subfamily *Hamaparvovirinae*. Bayesian inference trees were constructed using MrBayes v3.2, respectively based on amino acid sequences of NS1 of hamavirus; within trees, the viruses found in this study are labeled in blue. Scale bar indicates the amino acid substitutions per site. Genome organization of hamavirus is indicated. Purple rectangles: putative NS; red: putative VP.

In this study, 28 virus sequences belonging in 3 genera (*Ichthamaparvovirus, n* = 6; *Brevihamaparvovirus, n* = 1; *Chaphamaparvovirus, n* = 21) of the subfamily *Hamaparvovirinae* had been identified. The novel members of the subfamily *Hamaparvovirinae* had an approximately 4.4-kb genome with a similar monosense genomic organization. The nearly complete genome sequences of novel hamavirus included a partial 5′ untranslated region (UTR), the complete NS1 sequence, the complete NP overlapping with C terminus of NS1, the complete VP sequence, and a partial 3′ UTR (Fig. 4). Compared to other members in the*Parvoviridae* family, the 3′ UTR length of novel hamaviruses was very short (17–146 nt). Moreover, the typical LTR at the terminal of the genome and the conserved PLA2 domain in VP proteins werenot found in all members of novel hamaviruses.

The topologies of the tree showed that the hamaviruses formed 3 relatively independent branches (Fig. 4). Twenty-one genomes from 8 different bird species were phylogenetically grouped into the genus *Chaphamaparvovirus*. Five hamaviruses identified from 6 species of birds clustered together with members of the genera *Ichthamaparvovirus*. They were rather divergent from all other hamaviruses, and shared less than 35% aa similarity to the closest hamavirus, *Syngnathus scovelli*chapparvovirus.. One genome from Pallas's leaf warbler (*Phylloscopus proregulus*) was clustered with viruses belonging to the *Brevihamaparvovirus* genus.

### New viruses that may originate from parvovirus

**Figure 5:**
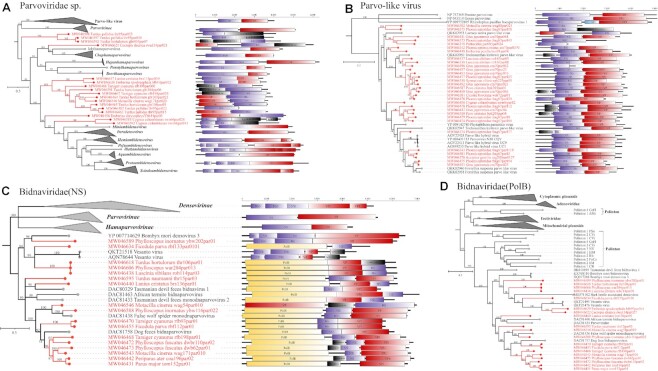
New viruses that may originate from parvovirus. (A) Bayesian inference tree established based on amino acid sequences of NS1 protein of unclassified *Parvoviridae*. (B) Bayesian inference tree established based on amino acid sequences of NS1 protein of Parvo-like virus. (C) Bayesian inference tree of NS1 of *Bidnaviridae*. (D) Bayesian inference tree of family B DNA polymerases from *Bidnaviridae*, Polintons, eukaryotic linear plasmids and viruses, and bacteriophages. Within trees, the viruses found in this study are labeled in red. Each scale bar indicates the amino acid substitutions per site. Purple rectangles: putative NS; red: putative VP; yellow: putative PolB.

In addition to the members of the family *Parvoviridae* identified above, we also discovered some viruses were too divergent to be grouped into any know genus. In the phylogenetic tree, 17 genomes formed a relatively distinct branch within *Parvoviridae*, which was separated from *Parvovirinae, Densovirinae*, and *Hamaparvovirinae* (Fig. 5A). They all had similar genomic organizations, either monosense or ambisense. But 2 (MW046628 and MW046637) of the monosense genomes were atypical, only 3.6 kb long, with the N-terminal of the capsid protein overlapping with the C-terminal of the nonstructural protein. Furthermore, 31 genomes from 12 different species of birds were closely related to a highly divergent DNA virus, named parvo-like hybrid virus (Fig. 5B), which had been found in the blood of seronegative hepatitis patients and in diatoms [54]. We also found another parvo-like virus that has been redefined as the family *Bidnaviridae* since it has a different genome organization and replication pattern from *Parvoviridae*. The novel members of the *Bidnaviridea* family have an approximately 6-kb-long genome that contains 3 major ORFs encoding capsid protein, nonstructural protein, and DNA polymerase of the family B (PolB) protein, respectively. DNA synthesis of the*Bidnaviride* family did not initiate by a self-priming mechanism but by using a PolB protein as a primer [55, 56]. A key point in bidnavirus evolution was the inheritance of a superfamily 3 helicase and a jelly-roll capsid protein from parvovirus and acquisition of the PolB from Polinton [57, 58]. Fig. 5C, D shows 2 phylogenetic trees of NS1 from bidnaviruses and parvoviruses and PolB from a wide range of viruses and plasmids. In this phylogeny, it is easy to see that the new bidnavirus NS proteins are clustered with the parvovirus family, and PolB proteins are closer to the Pollnton family than to other viruses and plasmids.

## Discussion

In the work that is presented here, we explored the viral nucleic acids enriched in cloacal swabs of wild birds and showed the prevalence and diversity of parvovirus dark matter.

The 170 new viruses identified in this study all had similar genomic structures, and no recombination events were found. One major ORF on the left side of the genome, encoding NS protein, is essential for virus packaging and replication and confers helicase, endonuclease, and DNA-binding functions [59, 60]. Another major ORF encodes capsid proteins that act as nuclear localization signals. The PLA2 enzyme domain allows the virus to be transported to the nucleus for replication without being lysed by late endosomes/lysosomes [61, 62]. Unlike other parvoviruses, aveparvovirus, amdoparvovirus, and all hamavirus VP1 do not have a PLA2. It had been reported that another membrane-penetrating mechanism dependent on divalent cations had evolved in the absence of PLA2 [63]. In phylogenetic analysis, the novel parvovirus NS1 proteins clustered with the previously established subfamily, but they formed a distinct lineage. In addition, the novel virus NS has only an average of 40% aa sequence homology with the NS1 proteins of currently known parvoviruses. The mapping analysis showed that there were highly similar parvoviruses among birds from the same habitat/location and among birds from different provinces, indicating that the virus had spread through birds. These results indicate that the novel parvoviruses are previously undetected dark matter, and future studies are needed to evaluate their potential for spillover to other species to better understand the risks to human health.

Here, 170 novel viruses were detected in avian cloaca samples using metagenomic analysis, but the real host origin of these new parvoviruses remains unknown. For example, 70 viruses belonged to the *Densovirinae* subfamily, which is thought to infect only arthropods. These newly identified viruses could therefore also be infecting birds, or they could simply be ingested and passed through the intestines temporarily without infecting birds, so we cannot exclude the possibility of a dietary origin of this virus. Although the samples in this study were from seemingly healthy wild birds, it has been shown that parvoviruses cause lethal disease in newly hatched chicks, young ducklings, and peafowl [59, 64]. Autonomic parvovirus DNA replicates in cells that are active in division, where they can use the DNA replication element portion of the host cell to accomplish their own replication. As a result, parvovirus often causes high morbidity and mortality in young hosts, and the same viruses generally cause asymptomatic or subclinical infections in adults [48, 60]. Hence, the epidemiology and taxonomy of these novel parvoviruses in these protected birds require further study.

Together, the present findings revealed unexpected diversity and the potential presence of parvovirus dark matter in the bird gut using viral metagenomics and a high-throughput strategy. Shedding light on viral dark matter will facilitate understanding of the evolution and biological characteristics of parvovirus.

## Supplementary Material

giad001_GIGA-D-22-00258_Original_Submission

giad001_GIGA-D-22-00258_Revision_1

giad001_Response_to_Reviewer_Comments_Original_Submission

giad001_Reviewer_1_Report_Original_Submission Wei Liu -- 10/30/2022 Reviewed

giad001_Reviewer_2_Report_Original_Submission Yanpeng Li -- 10/31/2022 Reviewed

giad001_Reviewer_2_Report_Revision_1 Yanpeng Li -- 11/29/2022 Reviewed

giad001_Supplemental_Figures_and_Tables

## Data Availability

The nucleotide sequences were deposited in the GenBank database, and the accession numbers are shown in Supplementary Table S2. The sequence raw data of bird cloaca samples were deposited into the NCBI sequence reads archive under accession number PRJNA600556. All data files that support our analysis were submitted to the *GigaScience* database [65].

## Additional Files

**Supplementary Fig. 1**. Bayesian inference tree established based on amino acid sequences of VP protein of *Densovirinae, Hamaparvovirinae*, Parvo-like virus, *Parvovirdae* sp., and *Bidnaviridae*. Within trees, the viruses found in this study are labeled in blue. Each scale bar indicates the amino acid substitutions per site.

**Supplementary Fig. 2**. The sampling sites are marked with colors. Samples were obtained from 5 provinces in China: Hunan, Zhejiang, Jiangsu, Jilin, and Heilongjiang.

**Supplementary Fig. 3**. The horizontal ordinate represents different virus genomes, while the longitudinal axis represents the pools that are arranged based on birds’ sampling sites. Heatmap representing the read number (in exponential form) in the mapping analysis using the genomes against NGS data of the libraries (see color legend).

**Supplementary Table 1**. Information of bird species and library included in the present study.

**Supplementary Table 2**. Information of viruses identified in cloaca of birds.

## Abbreviations

aa: amino acid; bp: base pair; ICTV: International Committee for the Taxonomy of Viruses; kb: kilobase; LTR: long terminal repeat; NCBI: The National Center for Biotechnology Information; NGS: next-generation sequencing; np: nucleoprotein; NS: nonstructural; nt: nucleotide; NVNR: nonvirus, nonredundant; ORF: open reading frame; PLA2: phospholipase A2; PolB: DNA polymerase of the family B; UTR: untranslated region.
