# Supplementary material for: Parvovirus dark matter in the cloaca of wild birds
Source: Gigascience. 2023 Feb 3;12:giad001. doi: 10.1093/gigascience/giad001 (PMC9896142; doi:10.1093/gigascience/giad001)
Supplement: giad001_GIGA-D-22-00258_Original_Submission [file giad001_giga-d-22-00258_original_submission.pdf]

GigaScience

Parvovirus dark matter in the cloaca of wild birds

--Manuscript Draft--

**Manuscript Number:**	GIGA-D-22-00258
**Full Title:**	Parvovirus dark matter in the cloaca of wild birds
**Article Type:**	Data Note
**Funding Information:**	Key Laboratory of Microbial Resources and Drug Development in Guizhou Province (2017YFC1200201) — Dr. Wen Zhang
**Abstract:**	With the development of viral metagenomics and next-generation sequencing technology, more and more novel parvoviruses even entirely new lineages have been identified in recent years. The Parvoviridae family includes a different group of viruses that can infect a wide variety of animals. In this study, systematically analyzed was performed to identity 'dark matter' of parvovirus and to explore its genetic diversity from wild birds' cloacal swab samples. We have tentatively defined parvovirus 'dark matter' as a highly divergent lineage in the Parvoviridae family. Consistent with all known parvovirus, these viruses showed several characteristics, including two major protein-coding genes and similar genome lengths. Moreover, we observed that novel parvo-like virus structurally like viruses in Parvoviridae but could not clustered with the established subfamilies in phylogenetic analysis. Furthermore, we observe that novel parvo-like virus structurally like Parvoviridae but could not cluster with the established subfamilies in phylogenetic analysis. We also found some new members associated with Bidnaviridae family, which may be derived from parvovirus. This suggests that systematic analysis of domestic and wild animal samples is necessary to explore the genetic diversity of parvoviruses and to mine for potential dark matter.
**Corresponding Author:**	Wen Zhang<br>Jiangsu University<br>Zhenjiang, Jiangsu CHINA
**Corresponding Author Secondary Information:**	
**Corresponding Author's Institution:**	Jiangsu University
**Corresponding Author's Secondary Institution:**	
**First Author:**	Ziyuan Dai
**First Author Secondary Information:**	
**Order of Authors:**	Ziyuan Dai
	Haoning Wang
	Haisheng Wu
	Qing Zhang
	Likai Ji
	Xiaochun Wang
	Quan Shen
	Shixing Yang
	Xiao Ma
	Tongling Shan
	Wen Zhang
**Order of Authors Secondary Information:**	

Powered by Editorial Manager® and ProduXion Manager® from Aries Systems Corporation

Additional Information:	
**Question**	**Response**
Are you submitting this manuscript to a special series or article collection?	Yes
Please select an option from the menu: as follow-up to "Are you submitting this manuscript to a special series or article collection?"	Functional Metagenomics
**Experimental design and statistics**<br><br>Full details of the experimental design and statistical methods used should be given in the Methods section, as detailed in our Minimum Standards Reporting Checklist. Information essential to interpreting the data presented should be made available in the figure legends.<br><br>Have you included all the information requested in your manuscript?	Yes
**Resources**<br><br>A description of all resources used, including antibodies, cell lines, animals and software tools, with enough information to allow them to be uniquely identified, should be included in the Methods section. Authors are strongly encouraged to cite Research Resource Identifiers (RRIDs) for antibodies, model organisms and tools, where possible.<br><br>Have you included the information requested as detailed in our Minimum Standards Reporting Checklist?	Yes
**Availability of data and materials**<br><br>All datasets and code on which the conclusions of the paper rely must be either included in your submission or deposited in publicly available repositories (where available and ethically appropriate), referencing such data using	Yes

a unique identifier in the references and in the “Availability of Data and Materials” section of your manuscript.<br><br>Have you have met the above requirement as detailed in our Minimum Standards Reporting Checklist?	

Powered by Editorial Manager® and ProduXion Manager® from Aries Systems Corporation

Manuscript

Click here to access/download;Manuscript;Parvovirus dark matter in the cloaca of wild birds.doc

Title:

Parvovirus dark matter in the cloaca of wild birds

Authors: Ziyuan Dai[2#], Haoning Wang[3#], Haisheng Wu[1,4#], Qing Zhang[4], Likai Ji[1], Xiaochun Wang[1], Quan Shen[1], Shixing Yang[1], Xiao Ma[4*], Tongling Shan[5*], Wen Zhang[1*]

1. Department of Laboratory Medicine, School of Medicine, Jiangsu University, Zhenjiang, Jiangsu, China

2.Department of Clinical Laboratory, Yancheng Third People's Hospital， Yancheng, Jiangsu,China

3. School of Geography and Tourism, Harbin University, Harbin, Heilongjiang, China

4. Qinghai Institute of Endemic Disease Prevention and Control, Xining, Qinghai, China

5. Shanghai Veterinary Research Institute, Chinese Academy of Agricultural Sciences, Shanghai, China.

* Correspondence:

Xiao Ma

maxiao0971@163.com

Tongling Shan

shantongling@shvri.ac.cn

Wen Zhang

z0216wen@yahoo.com

[#] Ziyuan Dai, Haoning Wang, Haisheng Wu contributed equally to this work.Keywords: Metagenomic, Parvoviridae, wild bird, dark matter

Repositories: The sequence raw data of bird fecal samples were deposited into the NCBI sequence reads archive under accession number PRJNA600556.

Parvovirus dark matter in the cloaca of wild birds

Abstract:

With the development of viral metagenomics and next-generation sequencing technology, more and more novel parvoviruses even entirely new lineages have been identified in recent years. The *Parvoviridae* family includes a different group of viruses that can infect a wide variety of animals. In this study, systematically analyzed was performed to identity 'dark matter' of parvovirus and to explore its genetic diversity from wild birds' cloacal swab samples. We have tentatively defined parvovirus 'dark matter' as a highly divergent lineage in the *Parvoviridae* family. Consistent with all known parvovirus, these viruses showed several characteristics, including two major protein-coding genes and similar genome lengths. Moreover, we observed that novel parvo-like virus structurally like viruses in *Parvoviridae* but could not clustered with the established subfamilies in phylogenetic analysis. Furthermore, we observe that novel parvo-like virus structurally like *Parvoviridae* but could not cluster with the established subfamilies in phylogenetic analysis. We also found some new members associated with *Bidnaviridae* family, which may be derived from parvovirus. This suggests that systematic analysis of domestic and wild animal samples is necessary to explore the genetic diversity of parvoviruses and to mine for potential dark matter.

Keywords:

Metagenomic, Parvoviridae, wild bird, dark matter

Introduction

The ongoing pandemic of SARS-CoV-2 poses a serious threat to human health and causes significant economic losses globally. It has been suggested that novel coronavirus originated in wild animals and infected humans through intermediate hosts such as bats and pangolins (1). Many emerging infectious diseases in humans are caused by pathogens originating from a wide variety of animals(2) and are dominated by zoonoses (60.3%): the majority of these (71.8%) originate in wildlife(3) and have increased significantly over time. Animal-derived human pathogens mainly arose from warm-blooded vertebrates, mammals and birds. (4) Birds' unique adaptive immune system makes them a natural reservoir for viruses (5) and allows asymptomatic infection and virus co-evolution to occur(6). The destruction of wetlands, the hunting and killing of migratory birds, and the increasing poultry consumption by human have facilitated avian viruses to cross species barriers to other populations that subsequently may bring the viruses to new areas (7). Birds may serve as vectors for disease vector transmission, as amplified hosts in the bird-vector-bird cycle, or as genetic sources for emerging cross-species viruses including Avian influenza viruses (8), West Nile Virus (9), Sindbis virus (10)

and Crimean-Congo hemorrhagic fever virus (11).The transmission of viruses from birds to poultry production (8) (12) than to humans, therefore, continues to be a threat to socio-economic and public health (13) and may cause severe morbidity and mortality(14).

Parvoviruses are non-enveloped, round, icosahedral symmetry viruses with an approximately 4 to 6 kb long single-stranded DNA genome with long inverted terminal repeats (LTR) at each end which can fold into hairpin-like structural related to expression and transcription strategies (15). Their overall genomic structure is relatively conservative: a non-structural (NS or Rep) ORF and structural (VP or Cap) ORF about half the length of the genome, respectively(16). The *Parvoviridae* family is divided into three subfamilies: the *Parvovirinae*, the *Densovirinae* and the *Hamaparvovirinae*. The *Parvovirinae* is further subdivided into ten genera: *Amdoparvovirus*, *Artiparvovirus*, *Aveparvovirus*, *Bocaparvovirus*, *Copiparvovirus*, *Dependoparvovirus*, *Erythroparvovirus*, *Loriparvovirus*, *Protoparvovirus* and *Tetraparvovirus*. The *Densovirinae* comprised eight genera: *Aquambidensovirus*, *Blattambidensovirus*, *Hemiambidensovirus*, *Iteradensovirus*, *Miniambidensovirus*, *Pefuambidensovirus*, *Protoambidensovirus* and *Scindoambidensovirus*. The newly established subfamily *Hamaparvovirinae* comprises five genera *Brevihamaparvovirus*, *Chaphamaparvovirus*, *Hepanhamaparvovirus*, *Ichthamaparvovirus* and *Penstylhamaparvovirus*(17).

There have been reports of parvoviruses in various countries from very diverse hosts, including mammals such as human (18), mouse (19), canine (20) and chimpanzees (21); arthropod such as crickets (22); and birds such as ducks (23), red-crowned cranes (24) and pigeon (2).Many species of *Parvoviridae* are known human or animal pathogens, but there are still lots of parvovirus sequences that cannot be accurately classified into a particular genus which requires their NS1 proteins share more than 85% amino acid sequence identity, according to the demarcation criteria of the International Committee for the Taxonomy of Viruses (ICTV) (17).

With the development of metagenome Next-Generation Sequencing technology, more and more viral pathogens have been detected, and the following problem is that most of the surveyed viral sequence (usually 60–95%) (25) cannot be aligned to any reference virus sequences (26) and/or cannot be obtained functional or taxonomic annotations, which is called “viral dark matter”.

Here, we analyzed the composition and distribution of divergent parvovirus in the intestinal tract of 3404 wild birds based on viral metagenomics to further explore the parvovirus “dark matter” to enrich known virus libraries and to explore their potential public health significance.

Materials and methods

Sample collection and preparation

A total of 3404 cloacal swabs of wild and breeding bird specimens were collected from 5 different provinces in China (Supplementary Fig.2), from 2018 to 2019(27). All specimens were shipped on dry ice. Cloacal swabs specimens were resuspended individually in 0.5mL phosphate-buffered saline (PBS) and vigorously vortexed for 5 min and centrifugated for 10 min, 15,000×g, the supernatant was then collected in microcentrifuge tube and stored at -80 °C. Sample pools were added about 0.1mL supernatant of each cloacal swab specimens from the same bird species. Subsequently, the supernatant was filtered through a 0.45 um filter (Millipore) to remove eukaryotic, giant viruses and bacterial cell-sized particles.

Viral metagenomic analysis

The filtrates enriched in viral particles were than treated with a cocktail of DNase, RNase, benzonase and Baseline-ZERO to digest unprotected nucleic acid at 37℃ for 90 min(28). Total nucleic acids were then extracted using QIAamp MinElute Virus Spin Kit (Qiagen) according to the manufacturer's protocol. 238 libraries were then constructed using a Nextera XT DNA Sample Preparation Kit (Illumina) and sequenced using the Illumina MiSeq platform with 250 bases paired ends with dual barcoding for each individual sample or sample pool. The information about each library was shown in Supplementary Table 1. For bioinformatics analysis, pair-end reads of 250 bp generated by Miseq were debarcoded using vendor software from Illumina. An in-house analysis pipeline running on a 32-node Linux cluster was used to process the data. Clonal reads were removed, and low-sequencing-quality tails were trimmed using a Phred quality score of ten as the threshold. Adaptors were trimmed using the default parameters of VecScreen which is NCBI BLASTn with specialized parameters designed for adapter removal. The cleaned reads were denovo assembled within each barcode using the ENSEMBLE assembler(29). Contigs and unassembled reads are then matched against a customized viral proteome database using BLASTx with an E-value cutoff of $<10^{-5}$, where the virus BLASTx database was compiled using NCBI virus reference proteome (ftp://ftp.ncbi.nih.gov/refseq/release/viral/) to which was added viral proteins sequences from NCBI nr fasta file (based on annotation taxonomy in Virus Kingdom). Candidate viral hits are then compared to an in-house non-virus non-redundant (NVNR) protein database to remove false positive viral hits, where the NVNR database was compiled using non-viral protein sequences extracted from NCBI nr fasta file (based on annotation taxonomy excluding Virus Kingdom). Contigs without significant BLASTx similarity to viral proteome database are searched against viral protein families in vFam database(30) using HMMER3(31-33) to detect remote viral protein similarities.

Analysis of the sequence

For assembly of the parvoviruses genomes, the contigs showing significant BLASTx similarity to parvoviruses were selected(34). The contigs with consensus sequence length >500 bp were subjected to further analysis where the individual contig was used as reference for mapping to the raw data of its original barcode using the Low Sensitivity/Fastest parameter in Geneious. Those prolonged contigs that had the major non-structural protein and structural protein, as well as some contigs only had a non-structural protein, were included in this study. The contig only had a putative non-structural protein were not showed in this study. Splice sites were also detected using Neural Network of the Berkeley Drosophila Genome Project. The search for protein homologies was made by BLAST programs at the NCBI website (http://www.ncbi.nlm.nih.gov/Blast.cgi) against the nonredundant protein database, and alignment of protein sequences was performed using the Mega 10.2.2.

Phylogenetic analysis

To investigate the evolutionary relationship of bird fecal parvovirus to other members of the family *Parvoviridae*, translated sequences from the coding region NS of wild bird fecal parvoviruses and reference sequences in GenBank were aligned using MUSCLE in MEGA v10.2.2 with default settings. Bayesian inference trees were then constructed using MrBayes v3.2 (35). The Markov chain was run for a maximum of 1 million generations, in which every 50 generations were sampled and the first 25% of Markov chain Monte Carlo (mcmc) samples were discarded as burn-in.

Results

Overview of Virome

This study included 3404 wild bird cloacal swab specimens belonging to 26 different families of birds. The 3404 samples were combined into 228 pools for viral metagenomic analysis, each of the pool was of the same species (Supplementary Table 1). After Illumina sequencing, a total of 46494515 reads showing similarity to viruses were obtained. The cellular organisms (archaea, bacteria, and eukaryotes) and other non-virion-associated reads were removed. As shown in Fig.1 A, there are about 36 families of viruses in the gut of wild birds, the first of which is parvovirus, 14068347 *Parvoviridae*-associated reads, approximately 30% of the total virus reads.

The sequence reads of the family *Parvoviridae* was selected for further analysis. The singlets and the de novo assembled contigs of 228 pools were compared to the GenBank nucleotide database using BLASTn to remove that showing significant similarity to known virus and finally obtained 170 viral contigs (1.4 to 7.0kb in length) (Supplementary Table 2). 70 out of the 170

contigs were mostly related to densovirus belonging to clades infecting arthropods. In addition, we obtained two Dependoparvovirus contigs and three Aveparvovirus contigs of the *Parvovirinae* subfamily which infect vertebrate hosts. There were 31 contigs is Parvo-like hybrid virus, 28 contigs belonging to the novel subfamily the *Hamaparvovirinae* and 17 contigs could not be assigned to an existing subfamily. Interestingly, we found 19 uncommon contigs may be new members of different genera within the *Bidnaviridae* family, which are thought to have evolutionarily derived from parvoviruses ancestor (36). Most Densovirnae, Bidnaviridae and unclassified Parvoviridae sequences shared between samples were related to passeriformes, while Parvovirinae sequences were less likely to be shared (Fig.1B).

Identification of novel viruses of the subfamily *Parvovirinae*

Among the ten genera of the *Parvovirinae* subfamily, five virus genomes from *Aveparvovirus* (n=3) and *Dependoparvovirus* (n=2) genera were found in cloaca of birds, and these two genera are known avian parvovirus. The poultry parvovirus, first identified in the early 1980s and later assigned to the genus *Aveparvovirus*, has been found worldwide in the intestines of young and healthy birds with intestinal syndrome (35). The dependoparvovirus, or adeno-associated virus (AAV) as it was originally known, are helper-dependent and require coinfection with a helper virus (herpesvirus or adenovirus) for productive infection.

Sequence analysis of the two nearly complete genome (MW046460 and MW046577) showed typical genomic size and organization which contained two major ORFs (Fig.2) The ORF located on the left side of the viral genome encodes the nonstructural protein about 600 aa. The ORF on the right side of the viral genome encodes about 700 aa capsid protein. In contrast to dependoparvovirus, the aveparvovirus does not contain the phospholipase A2 (PLA2) sequence motif, the VP1-unique region. Furthermore, a putative nucleoprotein (NP) was identified, in the middle of two major ORFs.

Phylogenetic analysis based on the complete NS1 amino acid sequences showed that these avian parvoviruses grouped into four different clades in the genus *aveparvovirus* and *dependoparvovirus* (Fig.2). Combined with the results of BLASTp, the similarity of five NS1 proteins with their most closely related viruses is all less than 65%, lower than the demarcation criteria of 85%, suggesting that these viruses are new species.

Identification of novel viruses of the subfamily *Denovirinae*

Densovirinae have one thing in common, capable of causing dense cores morphology changes. In fact, the entire subfamily was named densonucleosisviruses, “densoviruses” for short, because of this pathological feature (37). In recent years, the unexpected diversity of densovirus reveals along with the rapid development of high-throughput sequencing and viral metagenomics that emphasizes how little we know about its biological characteristics and evolutionary history.

We identified 70 densovirus genomes in this study. Most of them were rather divergent from all other densoviruses with aa identity of 49–65% except strain wag171par017 (MW046541), coa196par03 (MW046427), stc111par01 (MW046510) and gbt104par01 (MW046508) which sharing >85% sequence similarity. As shown in Fig.3A, the phylogenetic tree based on NS1 protein showed that the first 44 densoviruses we identified were clustered with members of seven genera of the subfamily *Densovirinae*, while the remaining 26 novel densoviruses (Densovirinae sp.) formed new clades that could not cluster with previously established genera. The NS1 gene is the most conserved of parvovirus gene sequences, while the VP genes are much more diverse. Accordingly, it is reasonable to speculate that the phylogenetic trees of these two genes may have some differences in their topological structure. Even so, the phylogenetic tree shows a similar topology across the board. Specifically, densoviruses have two main genomic structures: the monosense genome, which mainly includes the genus Iteradensovirus; and the ambisense genome, which mostly includes the remaining seven genera and unclassified densovirus. Just like Iteradensoviruses described previously, 20 novel monosense genomes contained three intronless genes with essentially identical positions but slightly different sizes. The largest, ORF1 had a coding capacity of 566-753 aa and the typical nucleoside triphosphatase (NTPase) motif for NS1. ORF2 with the PLA2 motif typical for VP1 had a coding capacity of 590-716 aa. ORF3 corresponded to NS2 with a 253-466 aa coding capacity and typically overlapped the N terminus of NS1. The novel densovirus genomes had an ambisens genome organization of 4514-6256 nucleotide long. In the clade of ambidensovirus, 40 novel densovirus genomes were exceptionally compact in size, including unusually small NS proteins and a predicted major capsid protein (Fig.3B). The NS cassette consisted of three genes on one strand, while a single or two genes encodes the structural proteins on the complementary strand. Interestingly, the PLA2 motif was absent in VP1 but found in the N-terminal region of VP2. It is possible that the leak scanning mechanism divided VP transcripts into VP1 and VP2.

Identification of novel viruses of the subfamily *Hamaparvovirinae*

In the past few years, a type of divergent parvoviruses has been identified in a broad range of host species, including wild rats, mice, domestic turkey, fish and dogs (19, 38-41). This divergent lineage was described under an unofficial umbrella term “Chapparvovirus” and grouped in unclassified Parvovirinae. In 2019, they were reclassified by ICTV and placed in the genus *Chaphamaparvovirus* of the newly proposed subfamily *Hamaparvovirinae*(17). The name "Hama" means "together" in Greek, reflecting the fact that their natural host infect both vertebrates and invertebrates.

In this study, 28 virus sequences belonging in three genera (*Ichthamaparvovirus*, n=6, *Brevihamaparvovirus*, n=1, *Chaphamaparvovirus*, n=21) of subfamily *Hamaparvovirinae* had been identified. The novel members of the subfamily *Hamaparvovirinae* had an approximately 4.4 kb genome with a similar monosense genomic organization. The nearly complete genome sequences of novel hamavirus included a partial 5′ untranslated region (UTR), the complete NS1 sequence, the complete NP overlapping with C terminus of NS1, the complete VP sequence and a partial 3’ UTR(Fig.4). Compared to other members in *Parvoviridae* family, the

3’ UTR length of novel hamaviruses were very short（17nt-146nt). Moreover, the typical LTR at the terminal of the genome and the conserved PLA2 domain in VP proteins was not found in all members of novel hamavirues.

The topologies of the tree showed that the hamavirues formed three relatively independent branches (Fig.4). 21 genomes from 8 different bird species were phylogenetically grouped into the genus *Chaphamaparvovirus*. Five hamaviruses identified from six species of birds clustered together with members of the genera *Ichthamaparvovirus*. was rather divergent from all other hamaviruses with <35% aa similarity with the closest hamavirus Syngnathus scovelli chapparvovirus. One genome from Pallas's Leaf Warbler (*Phylloscopus proregulus*) was clustered with viruses belonging to *Brevihamaparvovirus* genus.

New viruses that may originate from parvovirus

In addition to the members of the family *Parvoviridae* identified above, we also discovered some viruses were too divergent to be grouped into any know genus. In the phylogenetic tree, 17 genomes formed a relatively distinct branch within Parvoviridae which was separated from Parvovirinae, Densovirinae and Hamaparvovirinae(Fig.5A). They all had similar genomic organizations, either monosense or ambisense. But two (MW046628 and MW046637) of the monosense genomes were atypical, only 3.6 kb long, with the N-terminal of the capsid protein overlapping with the C-terminal of the non-structural protein. Besides, 31 genomes from 12 different species of birds were closely related to a highly divergent DNA virus, named parvo-like hybrid virus(Fig.5B), which had been found in the blood of seronegative hepatitis patients and in diatoms (42). We also found another parvo-like virus that has been redefined as the family *Bidnaviridae* since it has a different genome organization and replication pattern from Parvoviridae. The novel members of *Bidnaviridea* family with an approximately 6kb-long genome that contains three major ORFs encoding capsid protein, non-structural protein and DNA polymerase of the family B (PolB) protein, respectively. DNA synthesis of *Bidnaviride* family did not initiated by a self-priming mechanism but by using a PolB protein as a primer (43, 44). A key point in bidnavirus evolution was the inheritance of a superfamily 3 helicase and a jelly-roll capsid protein from parvovirus and acquisition of the PolB from Polinton (45, 46). Fig.5C and Fig.5D showed two phylogenetic trees of NS1 from bidnaviruses and parvoviruses and PolB from a wide range of viruses and plasmids. In this phylogeny, it was easy to see that the new bidnavirues NS proteins are clustered with the parvovirus family, and PolB proteins are closer to the Pollnton family than to other viruses and plasmids.

Nucleotide sequence accession number

The nucleotide sequences were deposited in the GenBank database under accession numbers MW046340 to MW046639. The sequence raw data of bird fecal samples were deposited into the NCBI sequence reads archive under accession number PRJNA600556.

Discussion

In the work that is presented here, we explored the viral nucleic acids enriched in cloacal swabs of wild birds and showed the prevalence and diversity of parvovirus dark matter.

The novel parvoviruses all have a similar genome structure. One major ORF on the left side of the genome, encoding NS protein, is essential for virus packaging and replication and confer helicase, endonuclease and DNA-binding functions (47, 48). Another major ORF encodes capsid proteins that act as nuclear localization signals. The PLA2 enzyme domain allows the virus to be transported to the nucleus for replication without being lysed by late endosomes/lysosomes (49, 50). Unlike other parvoviruses, aveparvovirus, amdoparvovirus and all hamavirus VP1 do not have a PLA2. It had been reported that another membrane-penetrating mechanism dependent on divalent cations had evolved in the absence of PLA2 (51). In phylogenetic analysis, the novel parvoviruses NS1 proteins clustered with the previously established subfamily, but they formed a distinct lineage. In addition, these novel viruses NS has only an average of 40% aa sequence homology with the NS1 proteins of currently known parvoviruses. These results indicate that the novel parvoviruses are previously undetected dark matter.

Here, 170 novel viruses were detected in avian cloaca samples using metagenomic sequence analysis, but the real host origin of these new parvoviruses remains unknown. For example, 70 viruses belonged to the *Densovirinae* subfamily, which only infects arthropods, these identified new viruses could infect birds, or they could simply be ingested and passed through the intestines temporarily without infecting birds, so we cannot exclude the possibility of a dietary origin of this virus. Although the samples in this study were from seemingly healthy wild birds, it has been shown that parvoviruses cause lethal disease in newly hatched chicks, young ducklings and peafowl (47) (52). Autonomic parvoviruses replicate DNA in cells that are active in division, where they can use the DNA replication element portion of the host cell to accomplish their own replication. As a result, parvovirus often causes high morbidity and mortality in young hosts, and the same viruses generally cause asymptomatic or subclinical infections in adults (37, 48). Hence, the epidemiology and taxonomy of these novel parvoviruses in these protected birds require further study.

Together, the present findings revealed unexpected diversity and the potential presence of parvovirus dark matter in the bird gut using viral metagenomics and high-throughput strategy. The shedding light on viral dark matter will facilitate the understanding of the evolution and biological characteristics of parvovirus.

Author statements

Conflict of interest

The authors declare that they have no confict of interest.

Ethical approval

We obtained cloacal swabs of wild and breeding bird in accordance with local laws and policies.

References

1. Xiao K, Zhai J, Feng Y, Zhou N, Zhang X, Zou J-J, et al. Isolation of SARS-CoV-2-related coronavirus from Malayan pangolins. Nature. 2020;583(7815):286-9.

2. Phan TG, Vo NP, Boros A, Pankovics P, Reuter G, Li OT, et al. The viruses of wild pigeon droppings. PLoS One. 2013;8(9):e72787.

3. Jones KE, Patel NG, Levy MA, Storeygard A, Balk D, Gittleman JL, et al. Global trends in emerging infectious diseases. Nature. 2008;451(7181):990-3.

4. Wolfe ND, Dunavan CP, Diamond J. Origins of major human infectious diseases. Nature. 2007;447(7142):279-83.

5. Chan JF, To KK, Tse H, Jin DY, Yuen KY. Interspecies transmission and emergence of novel viruses: lessons from bats and birds. Trends Microbiol. 2013;21(10):544-55.

6. Chan JF, To KK, Chen H, Yuen KY. Cross-species transmission and emergence of novel viruses from birds. Curr Opin Virol. 2015;10:63-9.

7. Olsen B, Munster VJ, Wallensten A, Waldenstrom J, Osterhaus AD, Fouchier RA. Global patterns of influenza a virus in wild birds. Science. 2006;312(5772):384-8.

8. Naguib MM, Verhagen JH, Mostafa A, Wille M, Li R, Graaf A, et al. Global patterns of avian influenza A (H7): virus evolution and zoonotic threats. FEMS Microbiol Rev. 2019;43(6):608-21.

9. Murray KO, Mertens E, Despres P. West Nile virus and its emergence in the United States of America. Vet Res. 2010;41(6):67.

10. Ziegler U, Fischer D, Eiden M, Reuschel M, Rinder M, Muller K, et al. Sindbis virus- a wild bird associated zoonotic arbovirus circulates in Germany. Vet Microbiol. 2019;239:108453.

11. Papa A, Tsergouli K, Tsioka K, Mirazimi A. Crimean-Congo Hemorrhagic Fever: Tick-Host-Virus Interactions. Front Cell Infect Microbiol. 2017;7:213.

12. Krammer F, Smith GJD, Fouchier RAM, Peiris M, Kedzierska K, Doherty PC, et al. Influenza. Nat Rev Dis Primers. 2018;4(1):3.

13. Fouchier RAM, Schneeberger PM, Rozendaal FW, Broekman JM, Kemink SAG, Munster V, et al. Avian influenza A virus (H7N7) associated with human conjunctivitis and a fatal case of acute respiratory distress syndrome. Proc Natl Acad Sci U S A. 2004;101(5):1356-61.

14. Wille M, Holmes EC. Wild birds as reservoirs for diverse and abundant gamma- and deltacoronaviruses. FEMS Microbiol Rev. 2020;44(5):631-44.

15. Brown KE. The expanding range of parvoviruses which infect humans. Rev Med Virol. 2010;20(4):231-44.

16. Pénzes JJ, de Souza WM, Agbandje-McKenna M, Gifford RJ. An ancient lineage of highly divergent parvoviruses infects both vertebrate and invertebrate hosts. 2019.

17. Penzes JJ, Soderlund-Venermo M, Canuti M, Eis-Hubinger AM, Hughes J, Cotmore SF, et al. Reorganizing the family Parvoviridae: a revised taxonomy independent of the canonical approach based on host association. Arch Virol. 2020;165(9):2133-46.

18. Phan TG, Vo NP, Bonkoungou IJ, Kapoor A, Barro N, O'Ryan M, et al. Acute diarrhea in West African children: diverse enteric viruses and a novel parvovirus genus. J Virol. 2012;86(20):11024-30.

19. Roediger B, Lee Q, Tikoo S, Cobbin JCA, Henderson JM, Jormakka M, et al. An Atypical Parvovirus Drives Chronic Tubulointerstitial Nephropathy and Kidney Fibrosis. Cell. 2018;175(2):530-43 e24.

20. Kapoor A, Mehta N, Dubovi EJ, Simmonds P, Govindasamy L, Medina JL, et al. Characterization of novel canine bocaviruses and their association with respiratory disease. The Journal of general virology. 2012;93(Pt 2):341-6.

21. Sharp CP, LeBreton M, Kantola K, Nana A, Diffo JLD, Djoko CF, et al. Widespread infection with homologues of human parvoviruses B19, PARV4, and human bocavirus of chimpanzees and gorillas in the wild. Journal of virology. 2010;84(19):10289-96.

22. Pham HT, Yu Q, Bergoin M, Tijssen P. A Novel Ambisense Densovirus, Acheta domesticus Mini Ambidensovirus, from Crickets. Genome Announc. 2013;1(6).

23. Vibin J, Chamings A, Klaassen M, Bhatta TR, Alexandersen S. Metagenomic characterisation of avian parvoviruses and picornaviruses from Australian wild ducks. Sci Rep. 2020;10(1):12800.

24. Wang Y, Yang S, Liu D, Zhou C, Li W, Lin Y, et al. The fecal virome of red-crowned cranes. Arch Virol. 2019;164(1):3-16.

25. Roux S, Hallam SJ, Woyke T, Sullivan MB. Viral dark matter and virus-host interactions resolved from publicly available microbial genomes. Elife. 2015;4.

26. Krishnamurthy SR, Wang D. Origins and challenges of viral dark matter. Virus Res. 2017;239:136-42.

27. Shan T, Yang S, Wang H, Wang H, Zhang J, Gong G, et al. Virome in the cloaca of wild and breeding birds revealed a diversity of significant viruses. Microbiome. 2022;10(1):60.

28. Zhang W, Li L, Deng X, Kapusinszky B, Pesavento PA, Delwart E. Faecal virome of cats in an animal shelter. J Gen Virol. 2014;95(Pt 11):2553-64.

29. Deng X, Naccache SN, Ng T, Federman S, Li L, Chiu CY, et al. An ensemble strategy that significantly improves de novo assembly of microbial genomes from metagenomic next-generation sequencing data. Nucleic Acids Res. 2015;43(7):e46.

30. Skewes-Cox P, Sharpton TJ, Pollard KS, DeRisi JL. Profile hidden Markov models for the detection of viruses within metagenomic sequence data. PloS one. 2014;9(8):e105067.

31. Eddy SR. A new generation of homology search tools based on probabilistic inference. Genome Inform. 2009;23(1):205-11.

32. Finn RD, Clements J, Eddy SR. HMMER web server: interactive sequence similarity searching. Nucleic Acids Res. 2011;39(Web Server issue):W29-W37.

33. Johnson LS, Eddy SR, Portugaly E. Hidden Markov model speed heuristic and iterative HMM search procedure. BMC Bioinformatics. 2010;11:431.

34. Kearse M, Moir R, Wilson A, Stones-Havas S, Cheung M, Sturrock S, et al. Geneious Basic: an integrated and extendable desktop software platform for the organization and analysis of sequence data. Bioinformatics. 2012;28(12):1647-9.

35. Ronquist F, Teslenko M, van der Mark P, Ayres DL, Darling A, Höhna S, et al. MrBayes 3.2: efficient Bayesian phylogenetic inference and model choice across a large model space. Syst Biol. 2012;61(3):539-42.

36. Krupovic M, Koonin EV. Evolution of eukaryotic single-stranded DNA viruses of the Bidnaviridae family from genes of four other groups of widely different viruses. Sci Rep. 2014;4:5347.

37. Johnson RM, Rasgon JL. Densonucleosis viruses (‘densoviruses’) for mosquito and pathogen control. Current Opinion in Insect Science. 2018;28:90-7.

38. Yang S, Liu Z, Wang Y, Li W, Fu X, Lin Y, et al. A novel rodent Chapparvovirus in feces of wild rats. Virol J. 2016;13:133.

39. Reuter G, Boros A, Delwart E, Pankovics P. Novel circular single-stranded DNA virus from turkey faeces. Arch Virol. 2014;159(8):2161-4.

40. Penzes JJ, de Souza WM, Agbandje-McKenna M, Gifford RJ. An Ancient Lineage of Highly Divergent Parvoviruses Infects both Vertebrate and Invertebrate Hosts. Viruses. 2019;11(6).

41. Palombieri A, Di Profio F, Lanave G, Capozza P, Marsilio F, Martella V, et al. Molecular detection and characterization of Carnivore chaphamaparvovirus 1 in dogs. Vet Microbiol. 2020;251:108878.

42. Naccache SN, Greninger AL, Lee D, Coffey LL, Phan T, Rein-Weston A, et al. The perils of pathogen discovery: origin of a novel parvovirus-like hybrid genome traced to nucleic acid extraction spin columns. J Virol. 2013;87(22):11966-77.

43. <Analysis of proteins encoded in the bipartite genome of a new type of parvo-like virus isolated from silkworm — structural protein with DNA polymerase motif.pdf>.

44. <Molecular cloning and expression of key gene encoding hypothetical DNA polymerase from B. mori parvo-like virus.pdf>.

45. Evolution of eukaryotic single-stranded DNA viruses of the Bidnaviridae family from genes of four other groups of widely different viruses.

46. Krupovic M, Koonin EV. Polintons: a hotbed of eukaryotic virus, transposon and plasmid evolution. Nat Rev Microbiol. 2015;13(2):105-15.

47. Kapgate SS, Kumanan K, Vijayarani K, Barbuddhe SB. Avian parvovirus: classification, phylogeny, pathogenesis and diagnosis. Avian Pathol. 2018;47(6):536-45.

48. Kailasan S, Agbandje-McKenna M, Parrish CR. Parvovirus Family Conundrum: What Makes a Killer? Annual Review of Virology. 2015;2(1):425-50.

49. A Viral Phospholipase A2 Is Required for Parvovirus Infectivity.

50. Girod A, Wobus CE, Zádori Z, Ried M, Leike K, Tijssen P, et al. The VP1 capsid protein of adeno-associated virus type 2 is carrying a phospholipase A2 domain required for virus infectivity. The Journal of general virology. 2002;83(Pt 5):973-8.

51. Penzes JJ, Pham HT, Chipman P, Bhattacharya N, McKenna R, Agbandje-McKenna M, et al. Molecular biology and structure of a novel penaeid shrimp densovirus elucidate convergent parvoviral host capsid evolution. Proc Natl Acad Sci U S A. 2020;117(33):20211-22.

52. Liu X, Wang H, Liu X, Li Y, Chen J, Zhang J, et al. Genomic and transcriptional analyses of novel parvoviruses identified from dead peafowl. Virology. 2020;539:80-91.

Figures and tables

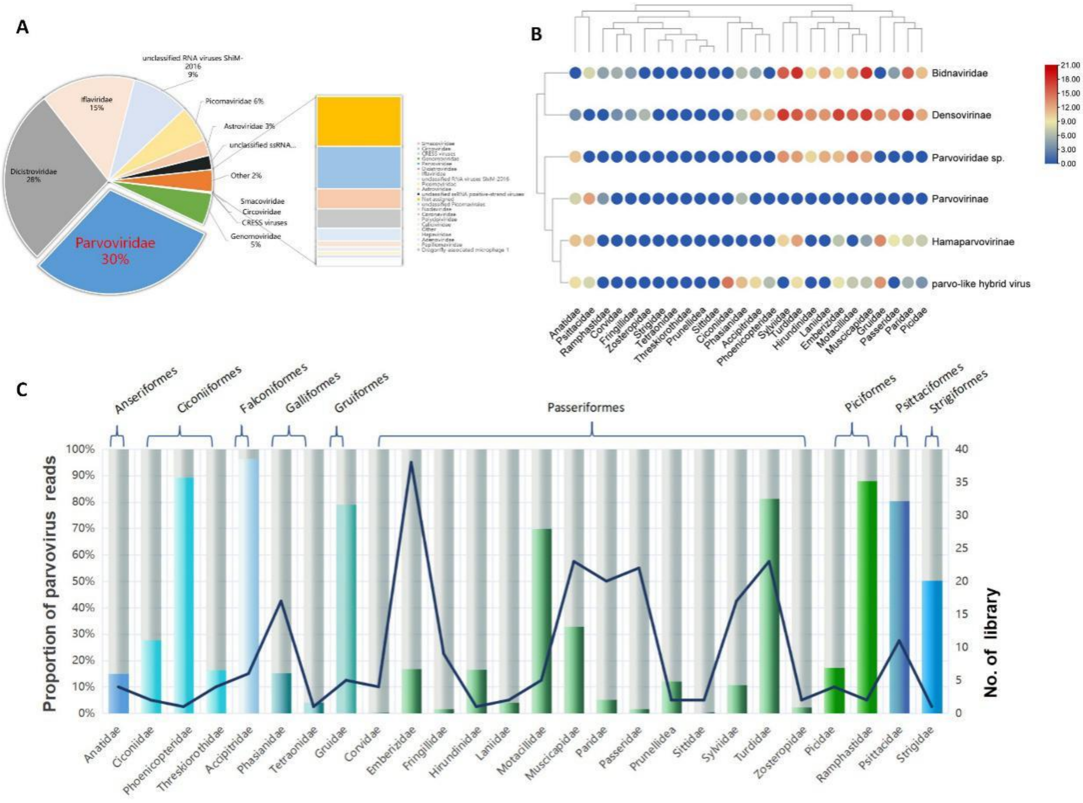

Fig 1. Overview of Virome

(A) Composition of each virus family in bird cloacal sample. (B)The shade of color in each circle shows the abundance of each group virus in each family of birds. The 170 viruses identified in this study were tentatively grouped into six groups.(C)Information of bird species and library.Colored bar shows the proportion of parvovirus reads of all viruses. The broken line shows the number of libraries for each family of birds.

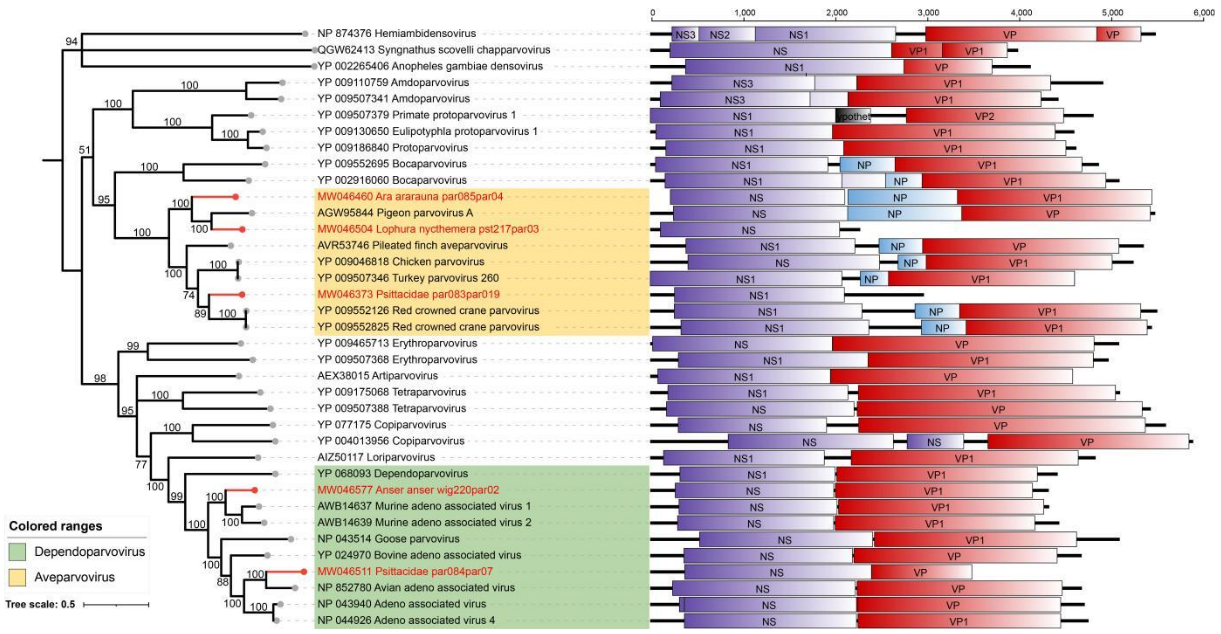

Fig2. Identification of novel viruses of the subfamily *Parvovirinae*

Bayesian inference trees were constructed using MrBayes v3.2 based on amino acid sequences of NS1 of parvovirus, within trees the viruses found in this study are labeled with red. Scale bar indicates the amino acid substitutions per site. Genome organization of each parvovirus was indicated.Purple rectangles: putative NS; red: putative VP; blue: putative NP.

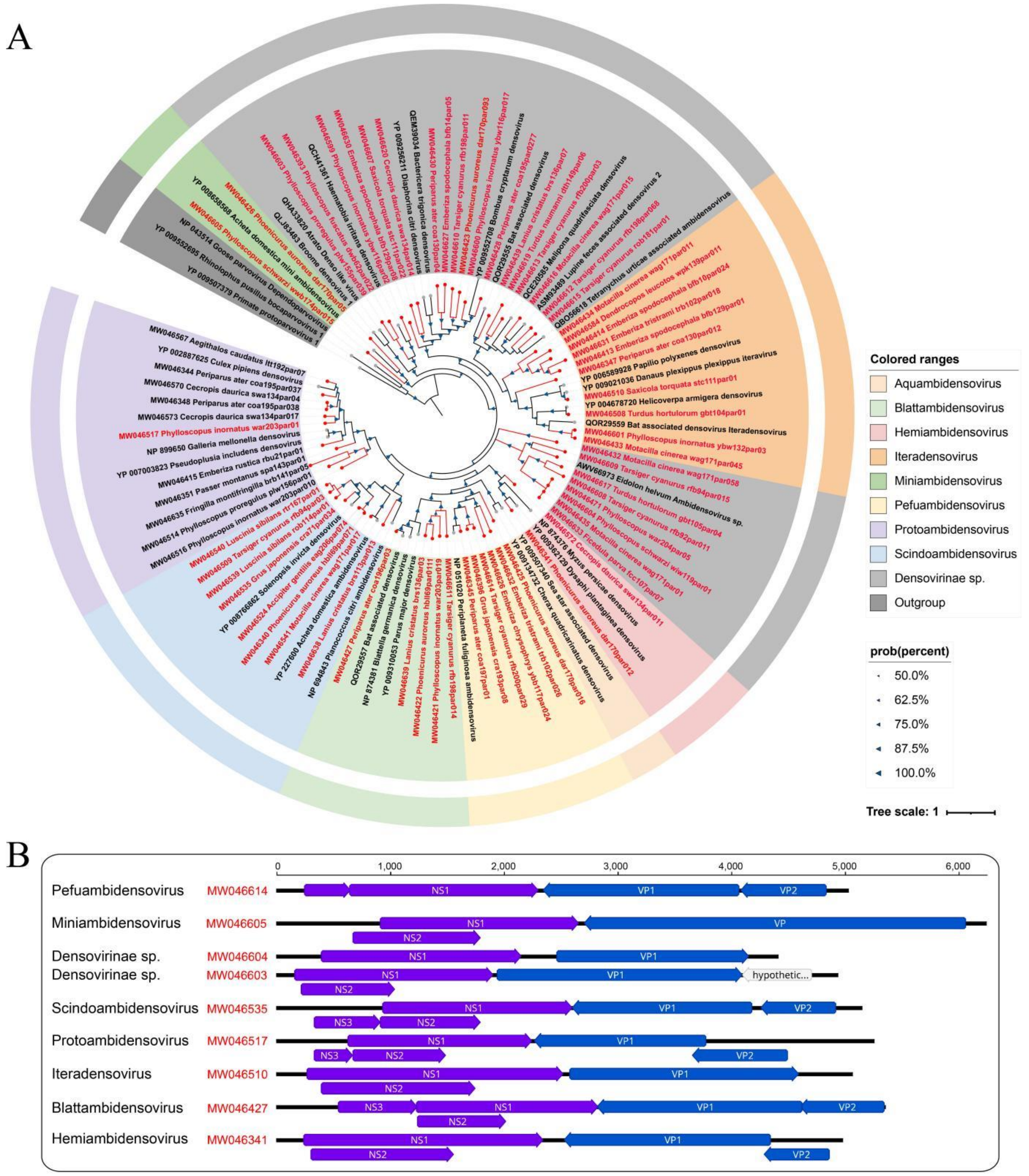

Fig3. Identification of novel viruses of the subfamily *Densovirinae*

(A) Bayesian inference trees were constructed using MrBayes v3.2 respectively based on amino acid sequences of NS1 of densovirus, within trees the viruses found in this study are labeled with red. Scale bar indicates the amino acid substitutions per site. (B) Genome organization of each genus are indicated. Purple arrows and rectangles: putative NS1; blue: putative VP.

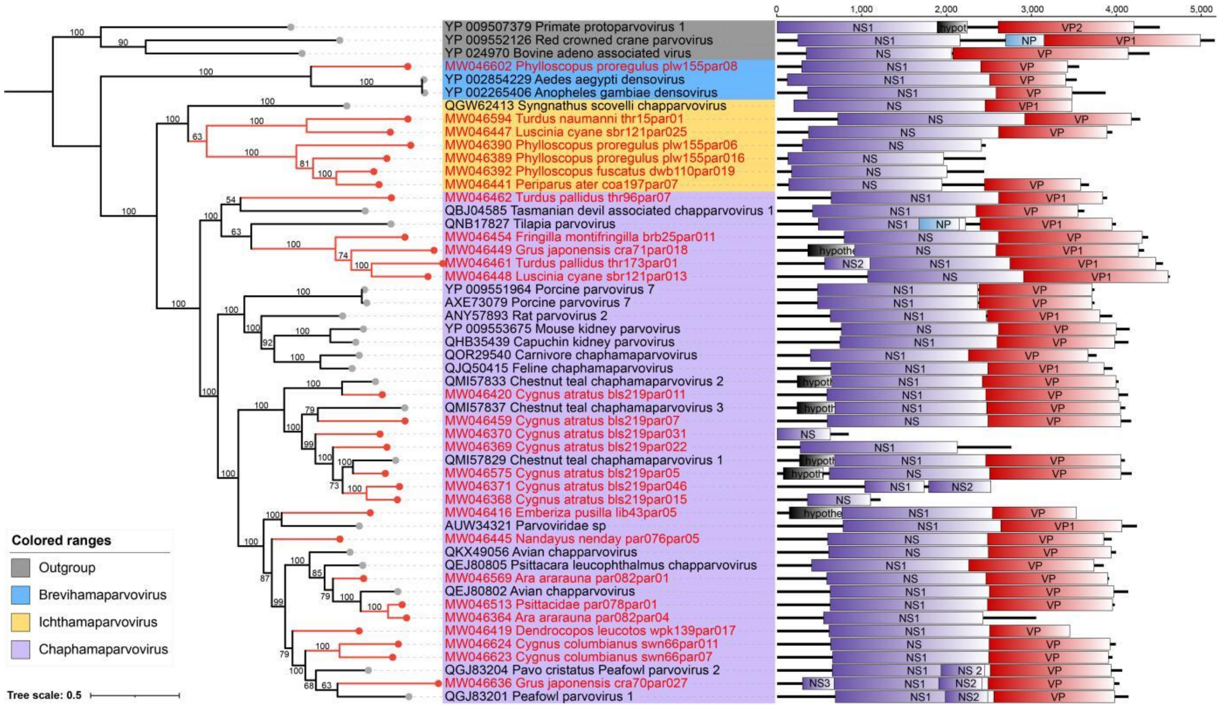

Fig4. Identification of novel viruses of the subfamily *Hamaparvovirinae*

Bayesian inference trees were constructed using MrBayes v3.2 respectively based on amino acid sequences of NS1 of hamavirus, within trees the viruses found in this study are labeled with blue. Scale bar indicates the amino acid substitutions per site. Genome organization of hamavirus are indicated. Purple rectangles: putative NS; red: putative VP.

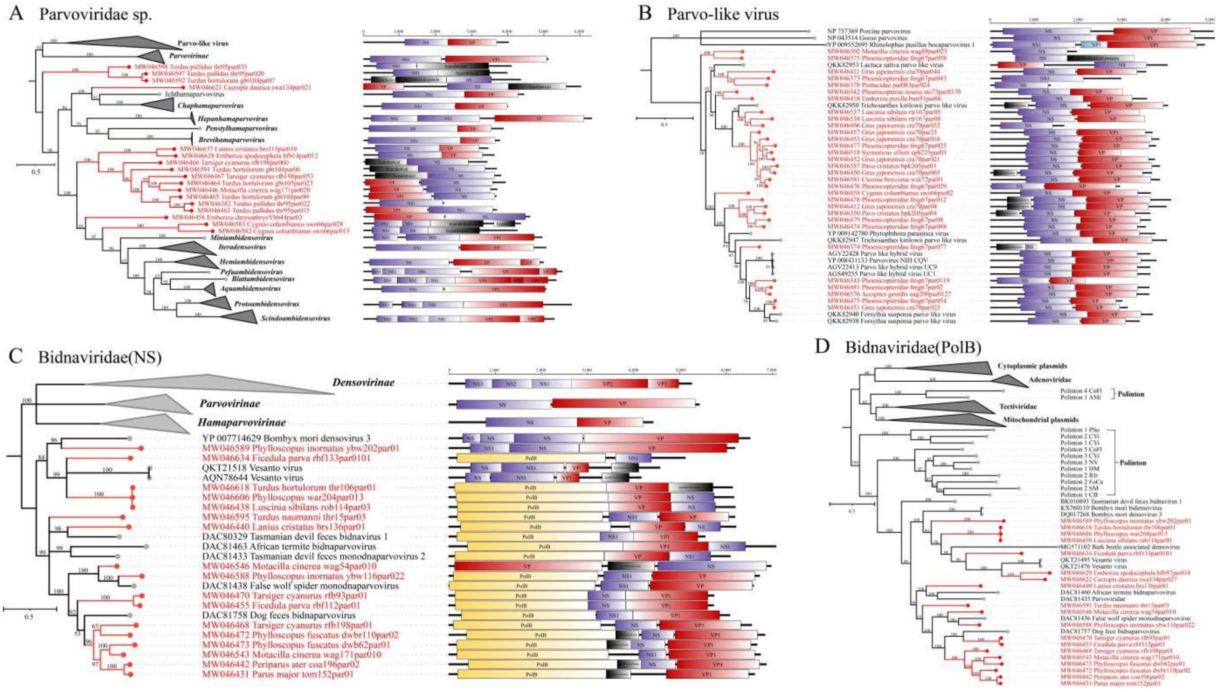

Fig5. New viruses that may originate from parvovirus

(A) Bayesian inference tree established based on amino acid sequences of NS1 protein of unclassified Parvoviridae. (B) Bayesian inference tree established based on amino acid sequences of NS1 protein of Parvo-like virus. (C) Bayesian inference tree of NS1 of Bidnaviridae.(D) Bayesian inference tree of family B DNA polymerases from Bidnaviridae, Polintons, eukaryotic linear plasmids and viruses, and bacteriophages.Within trees the viruses found in this study are labeled with red. Each scale bar indicates the amino acid substitutions persite. Purple rectangles: putative NS; red: putative VP; yellow: putative PolB.

Supplementary

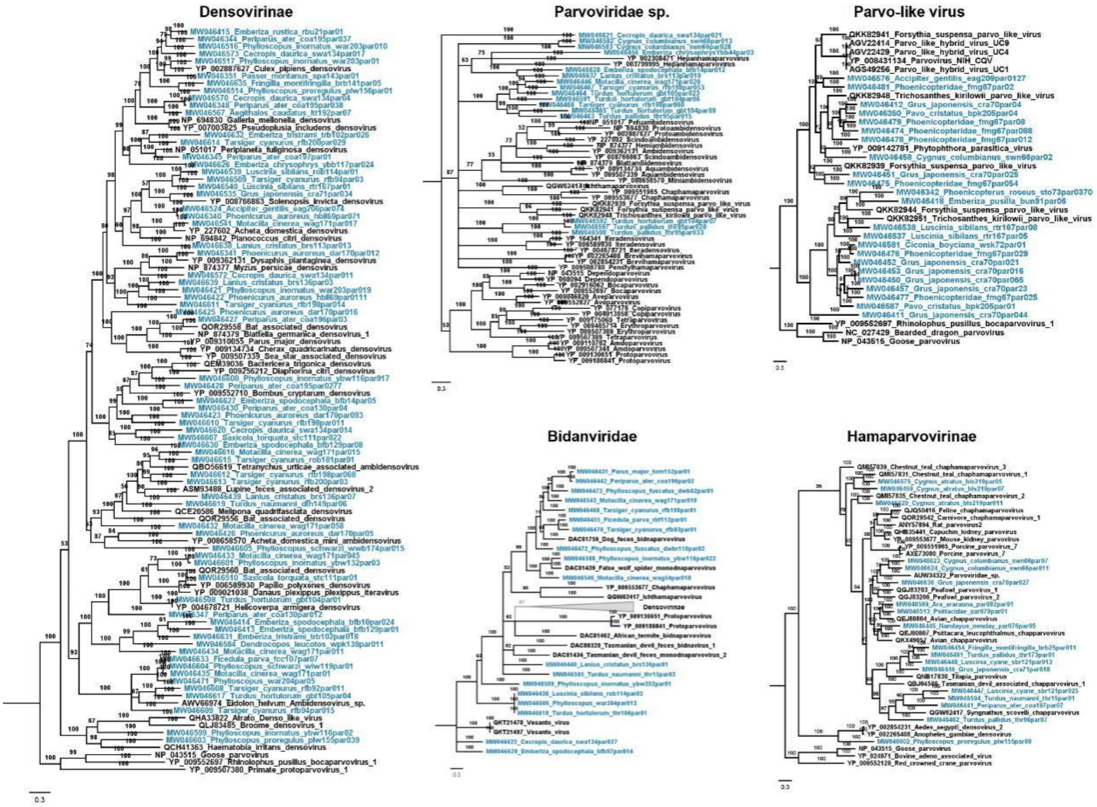

Supplementary Fig.1 The phylogenies of VP.

Bayesian inference tree established based on amino acid sequences of VP protein of Densovirinae, Hamaparvovirinae, Parvo-like virus, Parvovirdae sp. and Bidnaviridae Within trees the viruses found in this study are labeled with blue. Each scale bar indicates the amino acid substitutions per site.

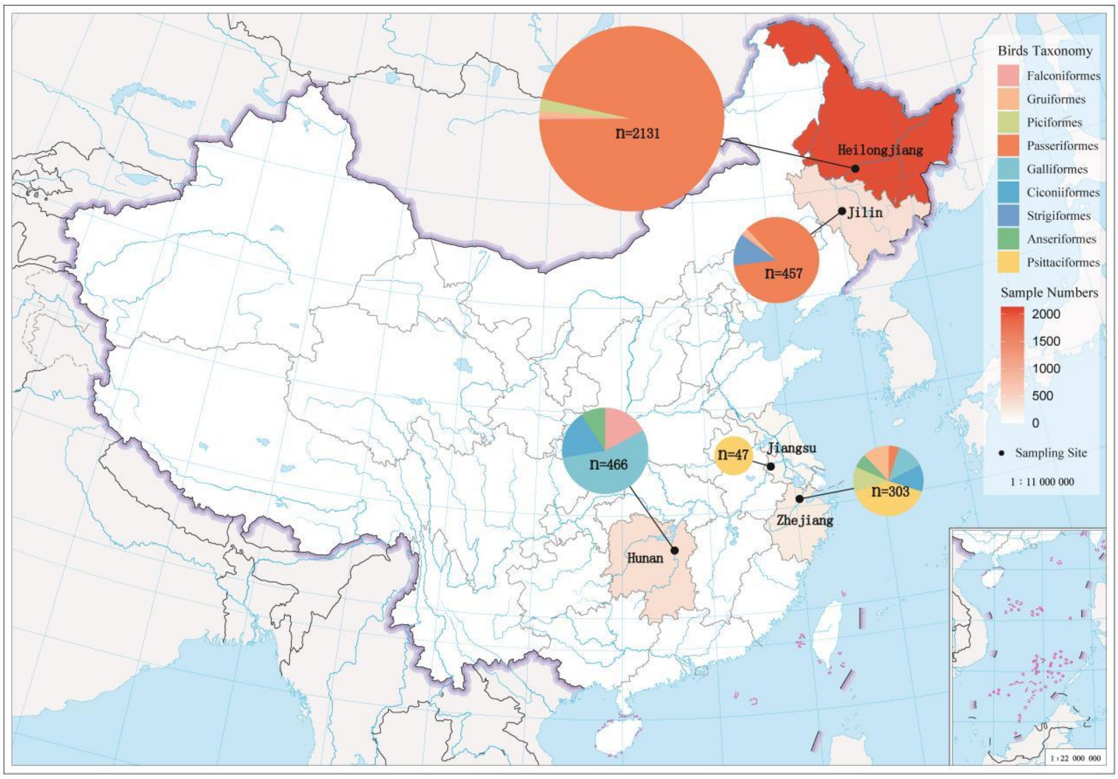

Supplementary Fig.2 Map of sampling locations.

The sampling sites are marked with colors. Samples were obtained from 5 sites in China: Hunan, Zhejiang, Jiangsu, Jilin and Heilongjiang.

Supplementary Table 1. Information of bird species and library included in the present study.

Supplementary Table 2. Information of viruses identified in cloaca of birds.

Figure 1 Click here to access/download;Figure;Fig1.jpg

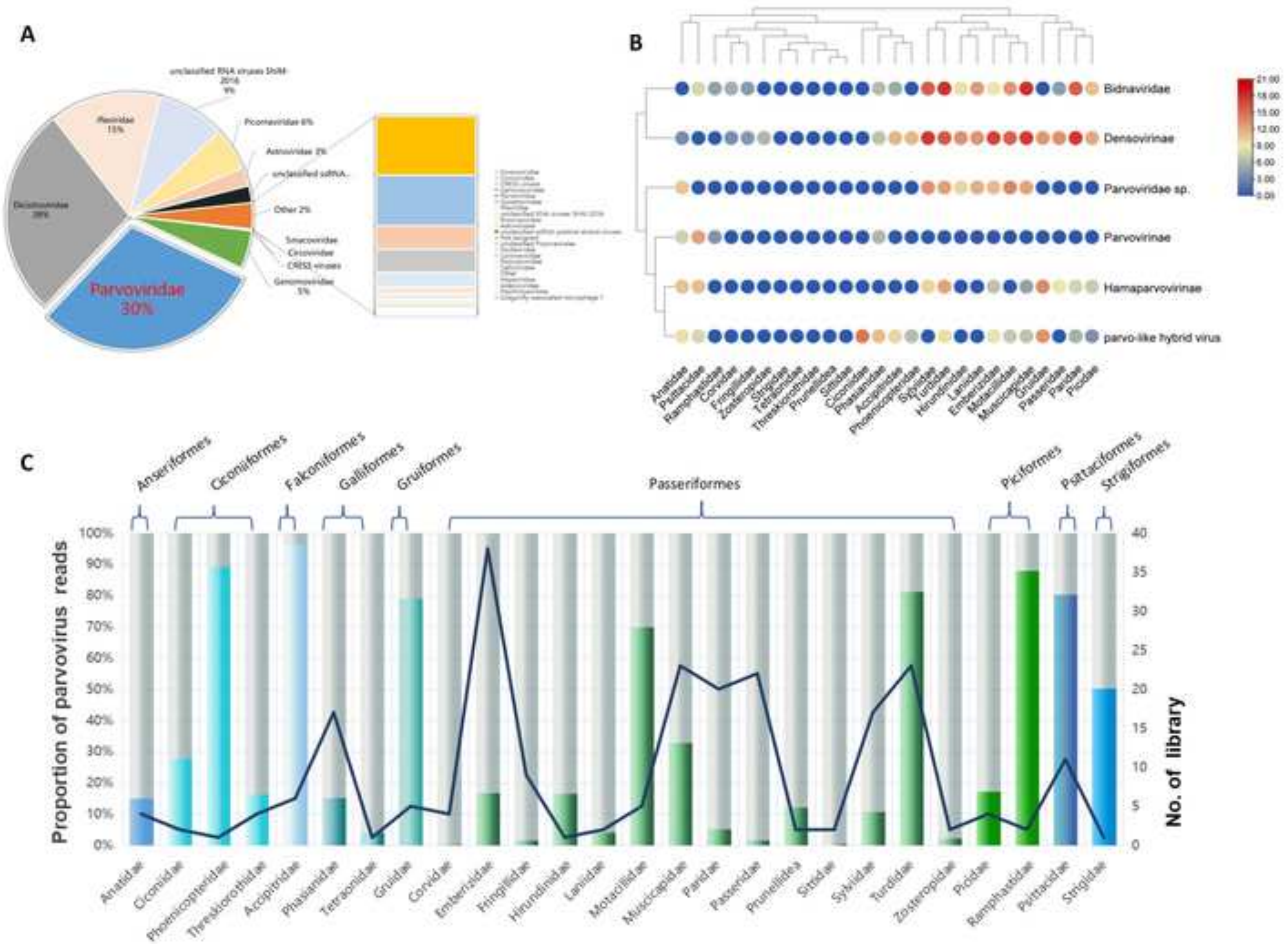

Figure 2

Click here to access/download;Figure;Fig 2 .jpg

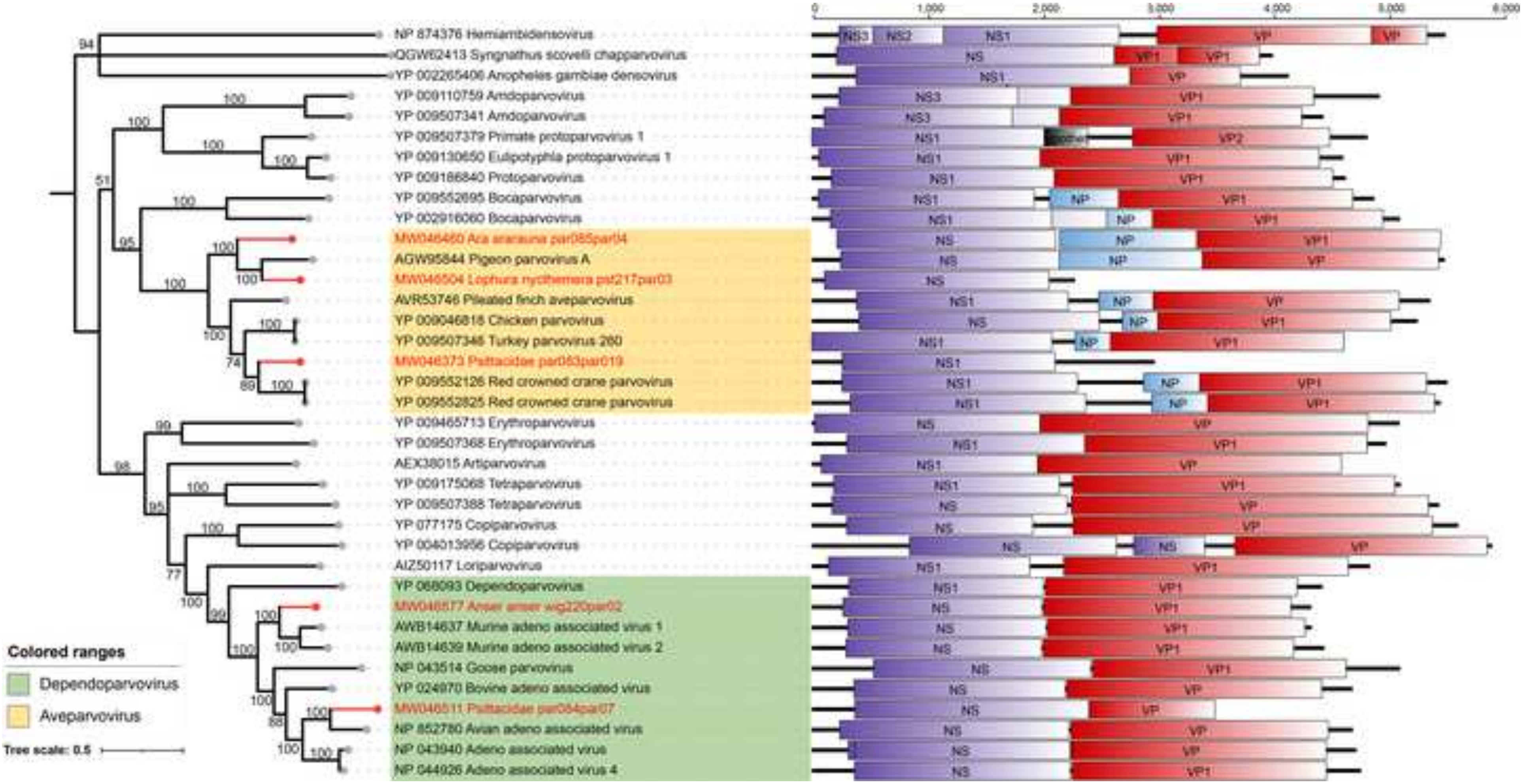

Figure 3 Click here to access/download;Figure;Fig 3 .jpg

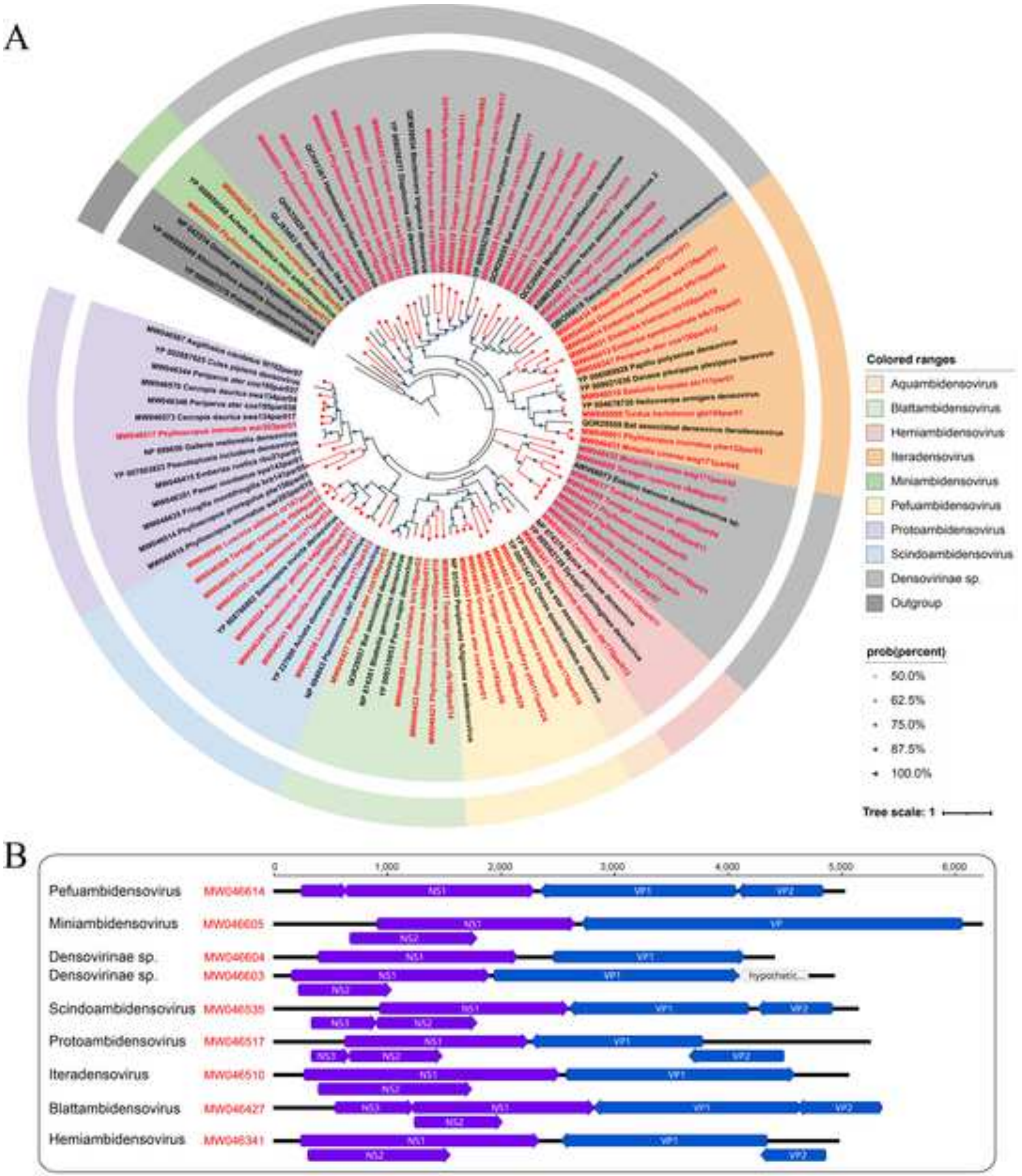

Figure 4

Click here to access/download;Figure;Fig 4 .jpg

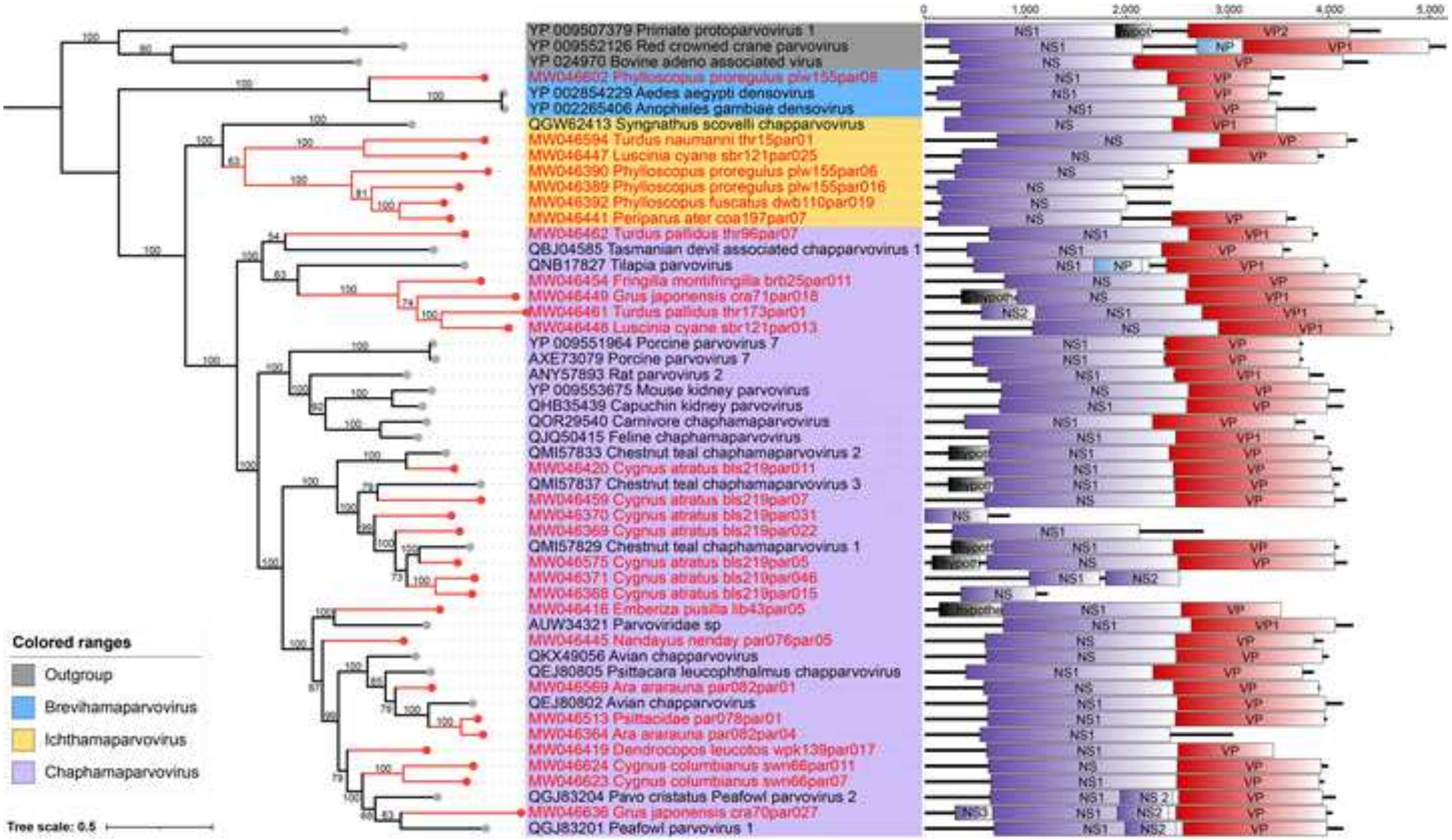

Figure 5

Click here to access/download;Figure;Fig 5 .jpg

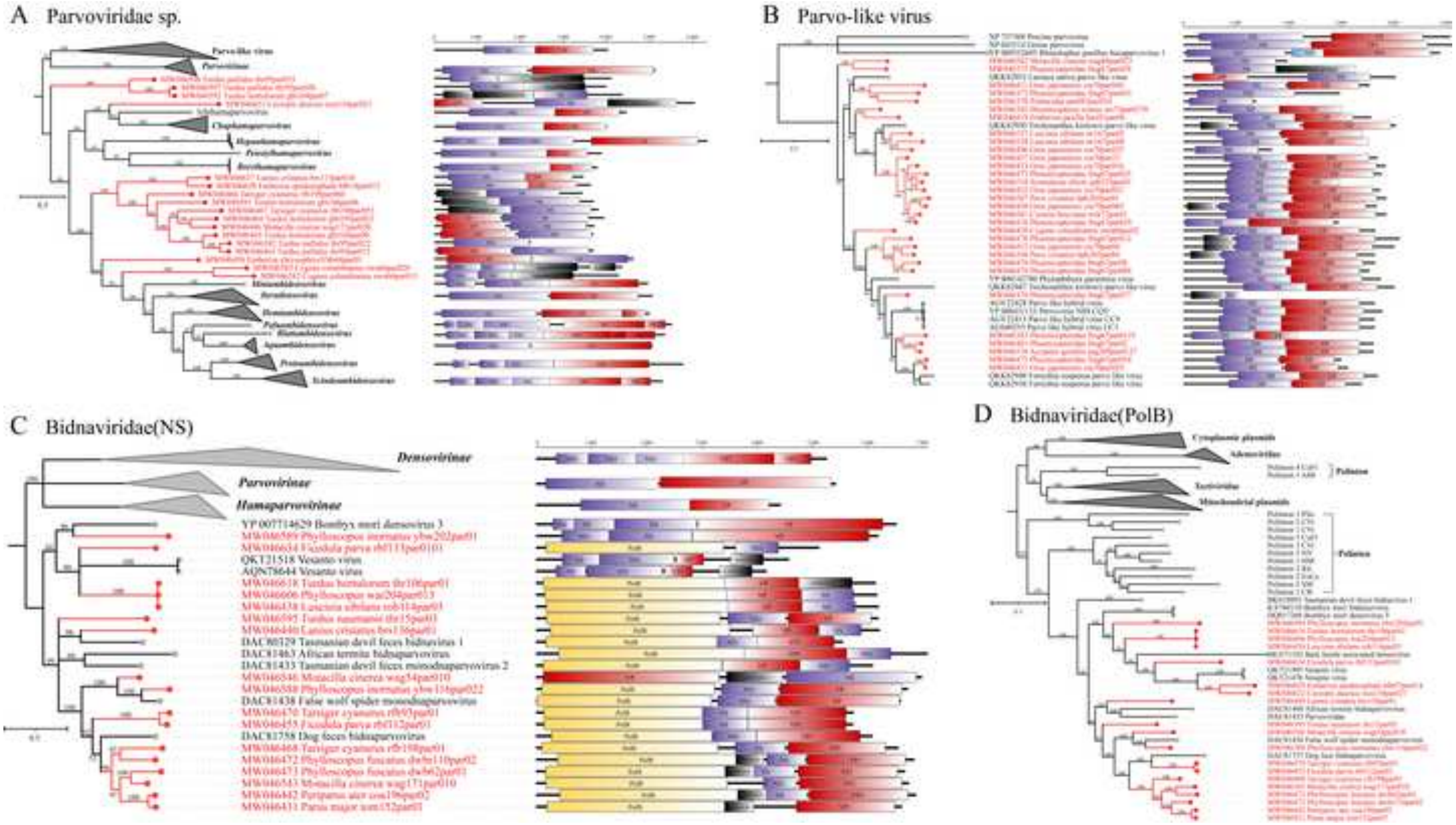

S Fig1

Click here to access/download;Figure;S Fig1 .jpg

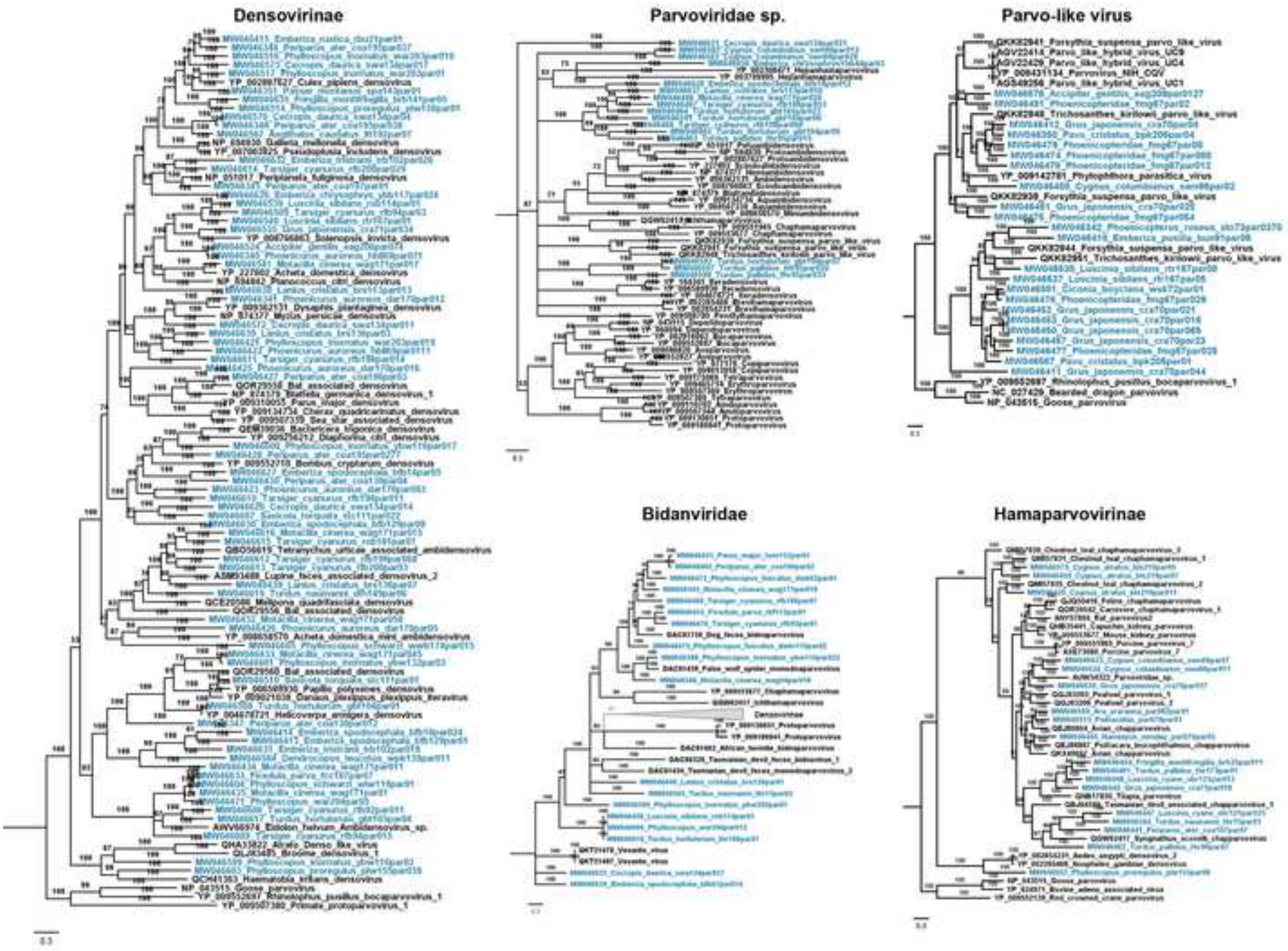

S Fig.2

Click here to access/download;Figure;S Fig.2 .jpg

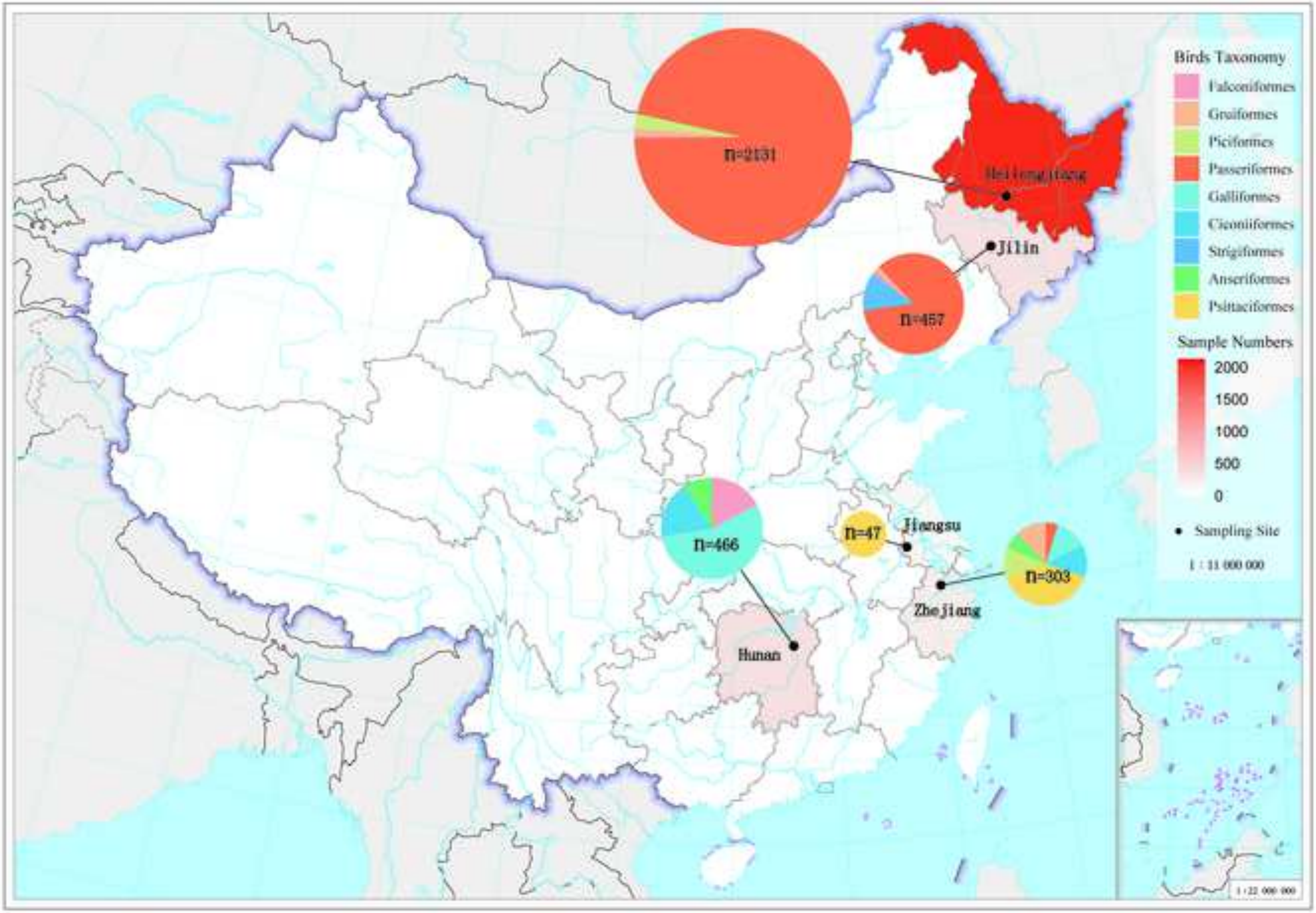

Supplementary Table 1

Click here to access/download
Supplementary Material
Supplementary Table 1.xlsx

Supplementary Table 2

Click here to access/download
Supplementary Material
Supplementary Table 2. .xlsx
