# Supplementary material for: Parvovirus dark matter in the cloaca of wild birds
Source: Gigascience. 2023 Feb 3;12:giad001. doi: 10.1093/gigascience/giad001 (PMC9896142; doi:10.1093/gigascience/giad001)
Supplement: giad001_GIGA-D-22-00258_Revision_1 [file giad001_giga-d-22-00258_revision_1.pdf]

# GigaScience

## Parvovirus dark matter in the cloaca of wild birds

--Manuscript Draft--

| | |
|---|---|
| **Manuscript Number:** | GIGA-D-22-00258R1 |
| **Full Title:** | Parvovirus dark matter in the cloaca of wild birds |
| **Article Type:** | Data Note |
| **Funding Information:** | Key Laboratory of Microbial Resources and Drug Development in Guizhou Province (2017YFC1200201) — Dr. Wen Zhang |
| **Abstract:** | With the development of viral metagenomics and next-generation sequencing technology, more and more novel parvoviruses even entirely new lineages have been identified in recent years. The Parvoviridae family includes a different group of viruses that can infect a wide variety of animals. In this study, systematically analyzed was performed to identity 'dark matter' of parvovirus and to explore its genetic diversity from wild birds' cloacal swab samples. We have tentatively defined parvovirus 'dark matter' as a highly divergent lineage in the Parvoviridae family. Consistent with all known parvovirus, these viruses showed several characteristics, including two major protein-coding genes and similar genome lengths. Moreover, we observed that novel parvo-like virus structurally like viruses in Parvoviridae but could not clustered with the established subfamilies in phylogenetic analysis. Furthermore, we observe that novel parvo-like virus structurally like Parvoviridae but could not cluster with the established subfamilies in phylogenetic analysis. We also found some new members associated with Bidnaviridae family, which may be derived from parvovirus. This suggests that systematic analysis of domestic and wild animal samples is necessary to explore the genetic diversity of parvoviruses and to mine for potential dark matter. |
| **Corresponding Author:** | Wen Zhang<br>Jiangsu University<br>Zhenjiang, Jiangsu CHINA |
| **Corresponding Author Secondary Information:** | |
| **Corresponding Author's Institution:** | Jiangsu University |
| **Corresponding Author's Secondary Institution:** | |
| **First Author:** | Ziyuan Dai |
| **First Author Secondary Information:** | |
| **Order of Authors:** | Ziyuan Dai |
| | Haoning Wang |
| | Haisheng Wu |
| | Qing Zhang |
| | Likai Ji |
| | Xiaochun Wang |
| | Quan Shen |
| | Shixing Yang |
| | Xiao Ma |
| | Tongling Shan |
| | Wen Zhang |
| **Order of Authors Secondary Information:** | |

Powered by Editorial Manager® and ProduXion Manager® from Aries Systems Corporation

| | |
|---|---|
| Response to Reviewers: | Reviewer #1: In this study, Dai and colleagues investigated the diversity of viruses in the cloaca samples from a large number of birds. The authors discovered an unexpected reservoir of pavoviridae family viruses among these species including several novel viruses. The paper is very interesting, I would like to support to be published; however, there are some issues that should be addressed.<br><br>1.Densoviruses are generally hosted by invertebrates. Here, 70 novel densoviruses or denso-like viruses were discovered from the bird digestive tract samples, which are often mixed with invertebrates as the later are often birds' diet. This means the host assignments of these densoviruses are not clear. I suggest that the authors should do is use the NGS data of these densovirus-positive libraries to try to identify possible invertebrate hosts of densoviruses in the samples (i.e. the sequence reads from invertebrates in the NGS data might provide useful host information for these densoviruses). This is one of the great benefits of shotgun sequencing over enrichment protocols: the former give you host data. For doing this, the authors can compare (using BLASTx searching) the contigs assembled from the NGS data of densovirus-positive libraries (those containing possible invertebrates sequence reads) against the total mitochondrial proteome database that can be downloaded from GenBank (mitochondrial gene is a good hallmark for species classification). The searching results can be displayed as a heatmap which indicates the composition of invertebrate species present in the samples.<br>=Thank you for this good advice. For better host assignment of these novel desoviruses or denso-like viruses, we compared (by using BLASTx searching) the NGS data of each densovirus-positive libraries against the total mitochondrial proteome database that were downloaded from GenBank. Based on the BLASTx results, we then analyzed the composition of invertebrate species in the densovirus-positive pools based on mitochondrial genes, which is shown as a heatmap in Fig 3C, we also included the possible host information of these densovirus in Fig 3A. Several sentences describing these results are located in line 241-248 in the revised manuscript.<br><br>2.Ethical concerns: (1) No information about permission and approval concerning bird sampling (e.g. licenses from wildlife conservation and/or management offices). Some of the species listed in this study belong to protected species. (2) No information on whether the experiments were conducted under bio-safety level 2 conditions.<br>=Ethical approvals were given by the Ethics Committee of Key Laboratory of Wildlife Diseases and Biosecurity Management of Heilongjiang Province with the reference number of WDBM2018-023, the Ethics Committee of Jiangsu University with the reference number of 2018ujs18023 and the Ethics Committee of Chinese Academy of Agricultural Sciences with the reference number of SVRI2017091. Sample collecting was performed in accordance with the Wildlife Protection Law of the People's Republic of China. All samples were shipped to the Shanghai Veterinary Research Institute of Chinese Academy of Agricultural Sciences where sample preparations were conducted in a biosafety level 2 laboratory. Please see lines 98-105.<br><br><br>3. Line 6 "identity" should be "identify"<br>=OK, we have corrected it. Please see line 6.<br>4.Lines 11-14. Redundant expressions?<br>=OK, we have deleted the redundant sentences.<br>5. Line 239 " initiated" should be "initiate"<br>=OK, we have corrected it. Please see line 289.<br><br><br>Reviewer #2: This manuscript by Dai et al. reports a large-scale metagenomic study of the fecal virome in wild birds, trying to explore the parvovirus "dark matter" to enrich known viral libraries and to explore their potential public health significance. The analyses were performed appropriately, but some issues need to be revised to meet the publication requirements.<br><br>Introduction<br>Line 55. Elaborate on what is known about pathology, prevalence, etc., of parvoviruses.<br>=OK, We describe the pathogenicity of GPV and MDPV, and describe the prevalence |

Powered by Editorial Manager® and ProduXion Manager® from Aries Systems Corporation

| | |
|---|---|
| | and transmission of parvoviruses. Please see lines 65-74.<br><br>Line 57. The threshold for species demarcation is 85%, what is the threshold for genus demarcation?<br>=NS1 proteins of members of the same genus should share at least 35-40% amino acid sequence identity with a coverage of >80% between any two members. Please see lines 53-60.<br><br><br>Materials and methods<br>Line 73. Change "vigorously" to the actual speed used.<br>=The "vigorously" have been changed to "1,800 rpm ". Please see line 92.<br><br><br>Line 74. The centrifuge speed is incorrect.<br>=The correct centrifugal force is “15,000× g ”. Please see line 93.<br><br><br>Line 75. "Sample collection and preparation" section does not properly explain the amount of fecal content represented in each one of the supernatants collected after preparation.<br>= Sample pools were added about 0.1mL supernatant of each cloacal swab specimens from the same bird species. Details of each pool can be found in Supplementary Table 1.<br><br>Line 88: Please describe the threshold that were used to define "low-sequencing-quality reads".<br>==Low-sequencing-quality reads, including low quality bases/reads, tag sequences, duplicates, etc. In NGS technology, the qualities of bases on most sequencing platforms will degrade as the run progresses, so it is common to see the quality of base calls falling towards the end of a read. Our research used 10 as the cutoff value to define "low-sequencing-quality reads".<br><br>Line 89. Why did the authors set the threshold to 10? Normally this value is 20.<br>=Our previous research used 10 as the cutoff value. Thank you for your professional reminder. We will use 20 as the cutoff value in future studies.<br><br>Line 91. why did the adapters get removed at the end of the bioinformatics pipeline and not after removing the barcodes?<br>=We generally remove adapters after trimming low-sequencing-quality tails, because we find that removing adapters before trimming low-sequencing-quality tails will cause more errors in de novo assembly.<br><br>Line 119. The phylogenetic analysis is missing a few important details. For instance, what does a Bayesian prior set? Were recombinant events investigated within the whole genome?<br>=We use the parameter “Aamodelpr” to set the rate matrix for amino acid data. Please see lines 148-150. No recombination events were found in the 170 new viral genomes identified in this study.<br><br>How to evaluate the best amino acid substitution model, and what amino acid substitution model was used to construct phylogenetic tree?<br>=In phylogenetic analysis, we used MrBayes to integrate over a predetermined set of fixed rate matrices and then summarize the MCMC samples and calculate the posterior probability estimate for each of these models. In our study, the particular posterior probability overwhelmingly supports the blosum model, so this maybe the only model sampled after the burn-in phase.<br><br><br>Results<br>Figure 1A, "unclassified RNA viruses-Shi M.2016" is not a proper taxa. Does it mean these RNA viruses are highly similar to the unclassified RNA viruses reported by Shi M?<br>= Thank you for your professional advice. We carefully reviewed the virus taxonomic |

Powered by Editorial Manager® and ProduXion Manager® from Aries Systems Corporation

| | |
|---|---|
| | information and replotted stacked bar graphs to show the composition of individual virus families in bird cloacal sample. Please see Fig.1A.<br><br>Line 159. The conserved PLA2 motif of VP1 was found and stated within the manuscript. Were the authors able to find conserved domains in NS1? Please refer to manuscript above for guidance with this motif.<br>= In NS1, several conserved domains were identified, including a replication initiator domain (xxHxHxxxxx), an SF3 helicase domain with an ATP- or GTP-binding Walker A loop (GxxxxGKT), Walker B loop (xxxxEE), and Walker B' loop (KxxxxGxxxxxxxK). These conservative domains are shown in Fig.2B.<br><br>Please indicate in the manuscript whether recombination events have been found. Birds could travel long distance and are major vectors for virus spread. Did they find any highly similar parvoviruses (potential transmission) among birds from the same habitat/location, and among birds from different provinces? This could be mentioned and discussed.<br>=No recombination events were found in the 170 new viral genomes identified in this study. Many parvoviruses were found among different birds at MES mountain, such as MW046591, MW046463 and MW046598, which grouped as unclassified Parvoviridae. Some highly similar viruses were also found in birds from different regions, such as strain fcc107par07 (MW046633) and wiw119par01 (MW046604), both belonging to the subfamily Densovirinae, were found in birds from Jilin and Heilongjiang provinces (Supplementary Fig. 3). Future studies are needed to evaluate their potential for spillover to other species to better understand the risks to human health. Please see lines 174-184.<br><br>In the results, archaea, bacteria, and phage … were removed. What the percentage of viruses reads before remove not viruses? And what are the proportions of RNA and DNA viruses in this study?<br>= In total, the 228 libraries generated 483,194,686 sequence reads. Virus sequence reads accounted for 9.62%. The total number of reads in each library was added in column G of Supplementary Table 1. As shown in Figure 1A, the proportion of RNA virus reads and DNA virus reads was 49.06% and 34.38%, respectively.<br><br>Discussion<br>Line 252. The viruses identified in this study were from cloacal swabs, please discuss whether or not cloacal swab is associated with infection within the birds.<br>=Thank you for your suggestion, please see lines 320 to 325. |
| **Additional Information:** | |
| **Question** | **Response** |
| Are you submitting this manuscript to a special series or article collection? | Yes |
| Please select an option from the menu:<br>as follow-up to "Are you submitting this manuscript to a special series or article collection?" | Functional Metagenomics |
| **Experimental design and statistics**<br><br>Full details of the experimental design and statistical methods used should be given in the Methods section, as detailed in our Minimum Standards Reporting Checklist. Information essential to interpreting the data presented should be made available in the figure legends. | Yes |

Powered by Editorial Manager® and ProduXion Manager® from Aries Systems Corporation

| | |
|---|---|
| Have you included all the information requested in your manuscript? | |
| **Resources**<br><br>A description of all resources used, including antibodies, cell lines, animals and software tools, with enough information to allow them to be uniquely identified, should be included in the Methods section. Authors are strongly encouraged to cite Research Resource Identifiers (RRIDs) for antibodies, model organisms and tools, where possible.<br><br>Have you included the information requested as detailed in our Minimum Standards Reporting Checklist? | Yes |
| **Availability of data and materials**<br><br>All datasets and code on which the conclusions of the paper rely must be either included in your submission or deposited in publicly available repositories (where available and ethically appropriate), referencing such data using a unique identifier in the references and in the "Availability of Data and Materials" section of your manuscript.<br><br>Have you have met the above requirement as detailed in our Minimum Standards Reporting Checklist? | Yes |

Manuscript

Click here to access/download;Manuscript;R3-Parvovirus dark matter in the cloaca of wild birds(1).docx

# Title: Parvovirus dark matter in the cloaca of wild birds

Authors: Ziyuan Dai[1,2#], Haoning Wang[3#], Haisheng Wu[1,4#], Qing Zhang[4], Likai Ji[1], Xiaochun Wang[1], Quan Shen[1], Shixing Yang[1], Xiao Ma[4]‡, Tongling Shan[5*], Wen Zhang[1*]

1. Department of Laboratory Medicine, School of Medicine, Jiangsu University, Zhenjiang, Jiangsu, China

2.Department of Clinical Laboratory, The Sixth Affiliated Hospital of Nantong University, Yancheng Third People's Hospital, Yancheng, Jiangsu, China

3. School of Geography and Tourism, Harbin University, Harbin, Heilongjiang, China

4. Qinghai Institute of Endemic Disease Prevention and Control, Xining, Qinghai, China

5. Shanghai Veterinary Research Institute, Chinese Academy of Agricultural Sciences, Shanghai, China

‡Senior author

Xiao Ma

maxiao0971@163.com

* Correspondence:

Tongling Shan: shantongling@shvri.ac.cn

Wen Zhang: z0216wen@yahoo.com

[#] Ziyuan Dai, Haoning Wang, Haisheng Wu contributed equally to this work.

ORCID IDs:
Ziyuan Dai: 0000-0002-3560-6091; Tongling Shan: 0000-0002-5329-6349; Wen Zhang: 0000-0002-9352-6153

Keywords: Viral Metagenomic, *Parvoviridae*, wild bird, dark matter

# Parvovirus dark matter in the cloaca of wild birds


## Abstract:

With the development of viral metagenomics and next-generation sequencing technology, more and more novel parvoviruses have been identified in recent years. Including even entirely new lineages. The *Parvoviridae* family includes a different group of viruses that can infect a wide variety of animals. In this study, systematic analysis was performed to identify the 'dark matter' (datasets that cannot be easily attributed to known viruses) of parvoviruses and to explore their genetic diversity from wild birds' cloacal swab samples. We have tentatively defined this parvovirus 'dark matter' as a highly divergent lineage in the *Parvoviridae* family. All parvoviruses showed several characteristics, including two major protein-coding genes and similar genome lengths. Moreover, we observed that the novel parvo-like viruses share similar genome organizations to most viruses in *Parvoviridae* but could not clustered with the established subfamilies in phylogenetic analysis. We also found some new members associated with the *Bidnaviridae* family, which may be derived from parvovirus. This suggests that systematic analysis of domestic and wild animal samples is necessary to explore the genetic diversity of parvoviruses and to mine for more of this potential dark matter.



## Keywords:

Metagenomic, *Parvoviridae*, wild bird, dark matter


## Introduction

The ongoing pandemic of SARS-CoV-2 poses a serious threat to human health and has caused significant global economic loss. It has been suggested that the novel coronavirus originated in wild animals and infected humans through intermediate hosts such as bats and pangolins (1). Many emerging infectious diseases in humans are caused by pathogens originating from a wide variety of animals(2) and are dominated by zoonoses (60.3%): the majority of these (71.8%) originate in wildlife(3) and have increased significantly over time (4, 5). Animal-derived human pathogens have mainly arisen from warm-blooded vertebrates, mammals and birds. (6) Birds' unique adaptive immune system makes them a natural reservoir for viruses (7) and allows asymptomatic infection and virus co-evolution to occur(8). The destruction of wetlands, the hunting and killing of migratory birds, and increasing poultry consumption by humans have facilitated avian viruses to cross species barriers to other populations that subsequently may bring the viruses to new areas (9). Birds may serve as vectors for disease vector transmission, as amplified hosts in the bird-vector-bird cycle, or as genetic sources for emerging cross-species viruses including Avian influenza viruses (10), West Nile Virus (11), Sindbis virus (12) and Crimean-Congo hemorrhagic fever virus (13).The transmission of viruses from birds to poultry

production (10) (14) then to humans, therefore, continues to be a threat to socio-economic and public health (15) and may cause severe morbidity and mortality(16).

Parvoviruses are non-enveloped, round, icosahedral symmetry viruses with an approximately 4 to 6 kb long single-stranded DNA genome. All parvoviruses have long inverted terminal repeats (LTR) at both the 5’ and 3’ ends that can fold into hairpin-like structures related to expression and transcription strategies (17). Their overall genomic structure is relatively conservative: a non-structural (NS or Rep) ORF (open reading frame) and structural (VP or Cap) ORF about half the length of the genome, respectively(18). The *Parvoviridae* family is divided into three subfamilies: the *Parvovirinae*, the *Densovirinae* and the *Hamaparvovirinae*. The *Parvovirinae* is further subdivided into ten genera: *Amdoparvovirus*, *Artiparvovirus*, *Aveparvovirus*, *Bocaparvovirus*, *Copiparvovirus*, *Dependoparvovirus*, *Erythroparvovirus*, *Loriparvovirus*, *Protoparvovirus* and *Tetraparvovirus*. The *Densovirinae* comprised eleven genera: *Aquambidensovirus*, *Blattambidensovirus*, *Diciambidensovirus*, *Hemiambidensovirus*, *Iteradensovirus*, *Miniambidensovirus*, *Pefuambidensovirus*, *Protoambidensovirus* and *Scindoambidensovirus*. The newly established subfamily *Hamaparvovirinae* comprises five genera *Brevihamaparvovirus*, *Chaphamaparvovirus*, *Hepanhamaparvovirus*, *Ichthamaparvovirus* and *Penstylhamaparvovirus*(19).

However, there are still lots of parvovirus sequences that cannot be accurately classified into a particular species or genus. According to the demarcation criteria of the International Committee for the Taxonomy of Viruses (ICTV), parvoviruses can be considered members of the same species if their NS1 proteins share >85% amino acid sequence identity. A genus can be identified as a group of species representing a single branch and share at least 35-40% amino acid sequence identity with a coverage of >80% between any two members (19).There have been reports of parvoviruses in various countries from very diverse hosts, including mammals such as human (20), mouse (21), canines (22) and chimpanzees (23); arthropods such as crickets (24); and birds such as ducks (25), red-crowned cranes (26) and pigeon (2).

Parvoviruses are often associated with the clinical signs of growth retardation and watery diarrhea in a wide range of animals and have been described in different species of birds (27). In the early 1960s, goose parvovirus (GPV) was identified in Europe, where it could cause a highly fatal disease of Muscovy ducklings and goslings called Derzsy’s disease. In 1989, another type of parvovirus, with symptoms similar to those of GPV, was isolated from Muscovy ducks and was named Muscovy duck parvovirus (MDPV). Both parvoviruses can cause substantial economic losses in waterfowl production and industry (28). Recent surveys have shown widespread distribution of parvoviruses in wild birds and commercial chicken around the world including China, South Korea, the United States and European countries including Hungary, Poland and Croatia (29-32). Due to the stability of the parvovirus in natural conditions, the virus can survive in feces or contaminated surfaces for up to a year, providing an additional source of infection to other animals (33). The maternally derived specific antibodies to parvovirus have been detected in hatching eggs and newly hatched ducklings, confirming the possibility of a potential vertical transmission of the virus (34).

With the development of next-generation sequencing (NGS) technology, more and more viral pathogens have been detected. However, most of the detected viral sequence (usually 60–95%) (35) cannot be aligned to any reference viral sequences (36), which are referred to as “viral dark matter” and may contain potential zoonotic pathogens. Therefore, mining "viral dark matter" can not only help us fill the "gap" in the evolutionary relationship between viruses and expand the range of known viral hosts, but also help us predict and monitor the prevalence of viral diseases that may occur in the future. Using viral metagenomics, we analyzed the composition and distribution of divergent parvovirus in the intestinal tract of 3,404 wild birds based on to further explore the parvovirus “dark matter” to enrich known virus libraries and to explore their potential public health significance.

# Materials and methods

## Sample collection and preparation

A total of 3,404 cloacal swabs of wild and breeding bird specimens were collected from 5 different provinces in China (Supplementary Fig.2), from 2018 to 2019(37). All specimens were shipped on dry ice. Cloacal swabs specimens were resuspended individually in 0.5mL phosphate-buffered saline (PBS) and vortexed at 1,800 rpm for 5 min and centrifugated for 10 min, 15,000× g, the supernatant was then collected in microcentrifuge tube and stored at -80 °C. Sample pools were added about 0.1mL supernatant of each cloacal swab specimens from the same bird species (Supplementary Table 1). Subsequently, the supernatant was filtered through a 0.45 µm filter (Millipore) to remove eukaryotic, giant viruses and bacterial cell-sized particles(38).

Ethical approvals were given by the Ethics Committee of Key Laboratory of Wildlife Diseases and Biosecurity Management of Heilongjiang Province with the reference number of WDBM2018-023, the Ethics Committee of Jiangsu University with the reference number of 2018ujs18023 and the Ethics Committee of Chinese Academy of Agricultural Sciences with the reference number of SVRI2017091. Sample collecting was performed in accordance with the Wildlife Protection Law of the People’s Republic of China. All samples were shipped to the Shanghai Veterinary Research Institute of Chinese Academy of Agricultural Sciences where sample preparations were conducted in a biosafety level 2 laboratory.

## Viral metagenomic analysis

The filtrates enriched in viral particles were than treated with a cocktail of DNase, RNase, benzonase and Baseline-ZERO to digest unprotected nucleic acid at 37℃ for 90 min(39). Total nucleic acids were then extracted using QIAamp MinElute Virus Spin Kit (Qiagen) according to the manufacturer’s protocol. 238 libraries were then constructed using a Nextera XT DNA Sample Preparation Kit (Illumina) and sequenced using the Illumina MiSeq platform (RRID:SCR_016379) with 250 bases paired ends with dual barcoding for each individual

sample or sample pool. The information about each library is shown in Supplementary Table 1. For bioinformatics analysis, pair-end reads of 250 bp generated by Miseq were debarcoded using vendor software from Illumina. An in-house analysis pipeline running on a 32-node Linux cluster was used to process the data. Clonal reads were removed, and low-sequencing-quality tails were trimmed using a Phred quality score of ten as the threshold. Adaptors were trimmed using the default parameters of VecScreen (NCBI BLASTn) with specialized parameters designed for adapter removal. The cleaned reads were *denovo* assembled within each barcode using the ENSEMBLE assembler(40). Contigs and unassembled reads are then matched against a customized viral proteome database using BLASTx with an E-value cutoff of $<10^{-5}$, where the virus BLASTx database was compiled using NCBI virus reference proteome (ftp://ftp.ncbi.nih.gov/refseq/release/viral/) to which viral proteins sequences were added from NCBI nr fasta file (based on annotation taxonomy in the Virus Kingdom). Candidate viral hits are then compared to an in-house non-virus non-redundant (NVNR) protein database to remove false positive viral hits, where the NVNR database was compiled using non-viral protein sequences extracted from NCBI nr fasta file (based on annotation taxonomy excluding the Virus Kingdom). Contigs without significant BLASTx similarity to viral proteome database are searched against viral protein families in vFam database(41) using HMMER3(RRID:SCR_005305)(42-44) to detect remote viral protein similarities(42-44).

## Analysis of the sequence

For assembly of the parvoviruses genomes, the contigs showing significant BLASTx similarity to parvoviruses were selected(45). The contigs with consensus sequence length >500 bp were subjected to further analysis where the individual contig was used as reference for mapping to the raw data of its original barcode using the Low Sensitivity/Fastest parameter in Geneious(RRID:SCR_010519)(45). Those prolonged contigs that had the major non-structural protein and structural protein, as well as some contigs only had a non-structural protein, were included in this study. The contig only had a putative non-structural protein were not showed in this study. Splice sites were also detected using Neural Network of the Berkeley Drosophila Genome Project. The search for protein homologies was made by BLAST programs at the NCBI website (http://www.ncbi.nlm.nih.gov/Blast.cgi) against the nonredundant protein database, and alignment of protein sequences was performed using the Mega 10.2.2(RRID:SCR_000667).

## Phylogenetic analysis

To investigate the evolutionary relationship of bird fecal parvovirus to other members of the family *Parvoviridae*, translated sequences from the coding region NS of wild bird fecal parvoviruses and reference sequences in GenBank were aligned using MUSCLE in MEGA v10.2.2 with default settings. Bayesian inference trees were then constructed using MrBayes v3.2 (RRID:SCR_012067)(46). During MrBayes analysis, we set “prset aamodelpr=mixed” for the phylogenetic analysis using amino acid sequences, which allows the program to utilize the

10 built-in amino acid models. The Markov chain was run for a maximum of 1 million generations, in which every 50 generations were sampled and the first 25% of Markov chain Monte Carlo (mcmc) samples were discarded as burn-in.

# Results

## Overview of the Virome

This study included 3,404 wild bird cloacal swab specimens belonging to 26 different families of birds. The 3,404 samples were combined into 228 pools for viral metagenomic analysis, each of the pools being of the same species (Supplementary Table 1). After Illumina sequencing, a total of 46,494,515 reads showing similarity to viruses were obtained, accounting for 9.62% of the total reads. The cellular organisms (archaea, bacteria, and eukaryotes) and other non-virion-associated reads were removed. As shown in Fig.1 A, the proportion of RNA virus reads and DNA virus reads was 49.06% and 34.38%, respectively. There were about 36 families of viruses in the gut of the wild birds, the highest of which is parvovirus, 14,068,347 *Parvoviridae*-associated reads, maxing approximately 30.26% of the total virus reads.

The sequence reads of the family *Parvoviridae* were selected for further analysis. The singlets and the *de novo* assembled contigs of 228 pools were compared to the GenBank nucleotide database using BLASTn to remove those showing significant similarity to known virus and finally obtained 170 viral contigs (1.4 to 7.0kb in length) (Supplementary Table 2). 70 out of the 170 contigs were mostly related to densovirus belonging to clades infecting arthropods. In addition, we obtained two Dependoparvovirus contigs and three Aveparvovirus contigs of the *Parvovirinae* subfamily which can infect vertebrate hosts. There were 31 contigs of Parvo-like hybrid virus, 28 contigs belonging to the novel subfamily the *Hamaparvovirinae* and 17 contigs could not be assigned to an existing subfamily. Interestingly, we found 19 uncommon contigs that may be new members of different genera within the *Bidnaviridae* family, which are thought to have evolutionarily derived from a parvoviruses ancestor (47). The mapping analysis using the 170 genomes against the 228 Next-generation sequencing data revealed the virus distribution in the 228 sample pools, where the distribution patterns were further analyzed based on birds' families (Fig.1B) and sampling sites (Supplementary Fig.3). Most Densovirnae, Bidnaviridae and unclassified Parvoviridae sequences shared between samples were related to passeriformes, while Parvovirinae sequences were less likely to be shared. In addition, many parvoviruses were found among different birds at MES mountain, such as MW046591, MW046463 and MW046598, which grouped as unclassified Parvoviridae. Some highly similar viruses were also found in birds from different regions, such as strain fcc107par07 (MW046633) and wiw119par01 (MW046604), both belonging to the subfamily *Densovirinae*, were found in birds from Jilin and Heilongjiang provinces (Supplementary Fig. 3).

## Identification of novel viruses of the subfamily *Parvovirinae*

Among the ten genera of the *Parvovirinae* subfamily, five virus genomes from *Aveparvovirus* (n=3) and *Dependoparvovirus* (n=2) genera were found in the cloaca of birds, and these two genera are known avian parvovirus. The poultry parvovirus, first identified in the early 1980s and later assigned to the genus *Aveparvovirus*, has been found worldwide in the intestines of young and healthy birds with intestinal syndrome (35). The dependoparvovirus, or adeno-associated virus (AAV) as it was originally known, are helper-dependent and require coinfection with a helper virus (herpesvirus or adenovirus) for productive infection.

Sequence analysis of the two nearly complete genomes (MW046460 and MW046577) showed typical genomic size and organization which contained two major ORFs (Fig.2A) The ORF located on the left side of the viral genome encodes the nonstructural protein about 600 aa. The ORF on the right side of the viral genome encodes about 700 aa capsid protein. As shown in Fig.2B, several conserved domains were identified, including a replication initiator domain (xxHxHxxxxx), an SF3 helicase domain with an ATP- or GTP-binding Walker A loop (GxxxxGKT), Walker B loop (xxxxEE), and Walker B' loop (KxxxxGxxxxxxxK). In contrast to dependoparvovirus, the aveparvovirus does not contain the phospholipase A2 (PLA2) sequence motif, the VP1-unique region. Furthermore, a putative nucleoprotein (NP) was identified, in the middle of two major ORFs.

Phylogenetic analysis based on the complete NS1 amino acid sequences showed that these avian parvoviruses grouped into four different clades in the genus *aveparvovirus* and *dependoparvovirus* (Fig.2). Combined with the results of BLASTp, the similarity of five NS1 proteins with their most closely related viruses is all less than 65%, lower than the demarcation criteria of 85%, suggesting that these viruses are new species.

## Identification of novel viruses of the subfamily *Denovirinae*

*Densovirinae* have in common the capacity of causing morphological "dense cores" (nuclei forming large cuboidal or circular inclusions). The entire viral subfamily being named densonucleosisviruses, "densoviruses" for short, because of this pathological feature (48). In recent years, the unexpected diversity of densovirus along with the rapid development of high-throughput sequencing and viral metagenomics has revealed how little we know about their biological characteristics and evolutionary history.

We identified 70 densovirus genomes in this study. Most of them were rather divergent from all other densoviruses with an aa identity of 49–65% except strain wag171par017 (MW046541), coa196par03 (MW046427), stc111par01 (MW046510) and gbt104par01 (MW046508) which shared >85% sequence similarity. As shown in Fig.3A, the phylogenetic tree based on NS1 protein showed that the first 44 densoviruses we identified clustered with members of seven genera of the subfamily *Densovirinae*, while the remaining 26 novel densoviruses (*Densovirinae* sp.) formed new clades that could not cluster with previously established genera.

The NS1 gene is the most conserved of parvovirus gene sequences, while the VP genes are much more diverse. It is therefore reasonable to speculate that the phylogenetic trees of these two genes may have some differences in their topological structure. Even so, the phylogenetic tree shows a similar topology across the board. Specifically, densoviruses have two main genomic structures: the monosense genome, which mainly includes the genus Iteradensovirus; and the ambisense genome, which mostly includes the remaining seven genera and unclassified densovirus. Just like Iteradensoviruses described previously, 20 novel monosense genomes contained three intronless genes with essentially identical positions but slightly different sizes. The largest, ORF1 had a coding capacity of 566-753 aa and the typical nucleoside triphosphatase (NTPase) motif for NS1. ORF2 with the PLA2 motif typical for VP1 had a coding capacity of 590-716 aa. ORF3 corresponded to NS2 with a 253-466 aa coding capacity and typically overlapped the N terminus of NS1. The novel densovirus genomes had an ambisens genome organization of 4514-6256 nucleotide long. In the clade of ambidensovirus, 40 novel densovirus genomes were exceptionally compact in size, including unusually small NS proteins and a predicted major capsid protein (Fig.3B). The NS cassette consisted of three genes on one strand, while a single or two genes encodes the structural proteins on the complementary strand. Interestingly, the PLA2 motif was absent in VP1 but found in the N-terminal region of VP2. It is possible that the leak scanning mechanism divided VP transcripts into VP1 and VP2.

Densoviruses are generally hosted by invertebrates(49), to study the host assignments of these novel densoviruses, densovirus-positive libraries in this study were included for further analysis. We compared the sequences of densovirus-positive libraries against the total mitochondrial proteome database that downloaded from GenBank. The results were displayed as a heatmap which indicates the composition of invertebrate species present in the samples. There were 23 pools that only contain mitochondrial sequences from a single species of Drosophila erecta. Our data indicated that the potential hosts of these 26 novel densoviruses in this study could be fruit fly (*Drosophila erecta*) (Fig. 3A and 3C).

## Identification of novel viruses of the subfamily *Hamaparvovirinae*

In the past few years, a type of divergent parvoviruses has been identified in a broad range of host species, including wild rats, mice, domestic turkey, fish and dogs (21, 50-53). This divergent lineage was described under an unofficial umbrella term “Chapparvovirus” and grouped in unclassified Parvovirinae. In 2019, they were reclassified by the ICTV and placed in the genus *Chaphamaparvovirus* of the newly proposed subfamily *Hamaparvovirinae*(19). The name "Hama" means "together" in Greek, reflecting the fact that their natural host infect both vertebrates and invertebrates.

In this study, 28 virus sequences belonging in three genera (*Ichthamaparvovirus*, n=6, *Brevihamaparvovirus*, n=1, *Chaphamaparvovirus*, n=21) of subfamily *Hamaparvovirinae* had been identified. The novel members of the subfamily *Hamaparvovirinae* had an approximately 4.4 kb genome with a similar monosense genomic organization. The nearly complete genome sequences of novel hamavirus included a partial 5′ untranslated region (UTR), the complete

NS1 sequence, the complete NP overlapping with C terminus of NS1, the complete VP sequence and a partial 3' UTR(Fig.4). Compared to other members in *Parvoviridae* family, the 3' UTR length of novel hamaviruses were very short（17nt-146nt). Moreover, the typical LTR at the terminal of the genome and the conserved PLA2 domain in VP proteins was not found in all members of novel hamaviruses.

The topologies of the tree showed that the hamaviruses formed three relatively independent branches (Fig.4). 21 genomes from 8 different bird species were phylogenetically grouped into the genus *Chaphamaparvovirus*. Five hamaviruses identified from six species of birds clustered together with members of the genera *Ichthamaparvovirus*. was rather divergent from all other hamaviruses with <35% aa similarity with the closest hamavirus *Syngnathus scovelli* chapparvovirus. One genome from Pallas's Leaf Warbler (*Phylloscopus proregulus*) was clustered with viruses belonging to *Brevihamaparvovirus* genus.

### New viruses that may originate from parvovirus

In addition to the members of the family *Parvoviridae* identified above, we also discovered some viruses were too divergent to be grouped into any know genus. In the phylogenetic tree, 17 genomes formed a relatively distinct branch within Parvoviridae which was separated from *Parvovirinae*, *Densovirinae* and *Hamaparvovirinae*(Fig.5A). They all had similar genomic organizations, either monosense or ambisense. But two (MW046628 and MW046637) of the monosense genomes were atypical, only 3.6 kb long, with the N-terminal of the capsid protein overlapping with the C-terminal of the non-structural protein. Furthermore, 31 genomes from 12 different species of birds were closely related to a highly divergent DNA virus, named parvo-like hybrid virus(Fig.5B), which had been found in the blood of seronegative hepatitis patients and in diatoms (54). We also found another parvo-like virus that has been redefined as the family *Bidnaviridae* since it has a different genome organization and replication pattern from Parvoviridae. The novel members of *Bidnaviridea* family with an approximately 6kb-long genome that contains three major ORFs encoding capsid protein, non-structural protein and DNA polymerase of the family B (PolB) protein, respectively. DNA synthesis of *Bidnaviride* family did not initiate by a self-priming mechanism but by using a PolB protein as a primer (55, 56). A key point in bidnavirus evolution was the inheritance of a superfamily 3 helicase and a jelly-roll capsid protein from parvovirus and acquisition of the PolB from Polinton (57, 58). Fig.5C and Fig.5D showed two phylogenetic trees of NS1 from bidnaviruses and parvoviruses and PolB from a wide range of viruses and plasmids. In this phylogeny, it was easy to see that the new bidnaviruses NS proteins are clustered with the parvovirus family, and PolB proteins are closer to the Pollnton family than to other viruses and plasmids.

## Discussion

In the work that is presented here, we explored the viral nucleic acids enriched in cloacal swabs of wild birds and showed the prevalence and diversity of parvovirus dark matter.

The 170 new viruses identified in this study all had similar genomic structures, and no recombination events were found. One major ORF on the left side of the genome, encoding NS protein, is essential for virus packaging and replication and confer helicase, endonuclease and DNA-binding functions (59, 60). Another major ORF encodes capsid proteins that act as nuclear localization signals. The PLA2 enzyme domain allows the virus to be transported to the nucleus for replication without being lysed by late endosomes/lysosomes (61, 62). Unlike other parvoviruses, aveparvovirus, amdoparvovirus and all hamavirus VP1 do not have a PLA2. It had been reported that another membrane-penetrating mechanism dependent on divalent cations had evolved in the absence of PLA2 (63). In phylogenetic analysis, the novel parvoviruses NS1 proteins clustered with the previously established subfamily, but they formed a distinct lineage. In addition, these novel viruses NS has only an average of 40% aa sequence homology with the NS1 proteins of currently known parvoviruses. The mapping analysis showed that there were highly similar parvoviruses among birds from the same habitat/location and among birds from different provinces, indicating that the virus had spread through birds. These results indicate that the novel parvoviruses are previously undetected dark matter and future studies are needed to evaluate their potential for spillover to other species to better understand the risks to human health.

Here, 170 novel viruses were detected in avian cloaca samples using metagenomic analysis, but the real host origin of these new parvoviruses remains unknown. For example, 70 viruses belonged to the *Densovirinae* subfamily, which in thought to only infect arthropods. These newly identified viruses could therefore also be infecting birds, or they could simply be ingested and passed through the intestines temporarily without infecting birds, so we cannot exclude the possibility of a dietary origin of this virus. Although the samples in this study were from seemingly healthy wild birds, it has been shown that parvoviruses cause lethal disease in newly hatched chicks, young ducklings and peafowl (59) (64). Autonomic parvoviruses DNA replicates in cells that are active in division, where they can use the DNA replication element portion of the host cell to accomplish their own replication. As a result, parvovirus often cause high morbidity and mortality in young hosts, and the same viruses generally cause asymptomatic or subclinical infections in adults (48, 60). Hence, the epidemiology and taxonomy of these novel parvoviruses in these protected birds requires further study.

Together, the present findings revealed unexpected diversity and the potential presence of parvovirus dark matter in the bird gut using viral metagenomics and a high-throughput strategy. The shedding light on viral dark matter will facilitate the understanding of the evolution and biological characteristics of parvovirus.

## Acknowledgments

This work was supported by the National Key Research and Development Programs of China under No. 2022YFC2603801 and the Special Funds for Science Development of the Clinical Teaching Hospitals of Jiangsu Vocational College of Medicine under No.20229152.

# Abbreviations

aa: amino acid; AAV: adeno-associated virus; LTR: long terminal repeats; NGS: next-generation sequencing; np: nucleoprotein; NS: non-structural; NVNR: non-virus non-redundant; ORF: Open Reading Frame; PolB: DNA polymerase of the family B; UTR untranslated region

# Author statements

## Conflict of interest

The authors declare that they have no conflicts of interest.

## Ethical approval

We obtained cloacal swabs of wild and breeding bird in accordance with local laws and policies.

**Funding**

Wen Zhang was supported by National Key Research and Development Programs of China grant 2022YFC2603801, and Ziyuan Dai by The Special Funds for Science Development of the Clinical Teaching Hospitals of Jiangsu Vocational College of Medicine, grant 20229152

# Data availability

The nucleotide sequences were deposited in the GenBank database, and the accession numbers are shown in Supplementary Table 2. The sequence raw data of bird cloaca samples were deposited into the NCBI sequence reads archive under accession number PRJNA600556. All data files that support our analysis were submitted to the *GigaScience* database (65).

## References


1. Xiao K, Zhai J, Feng Y, Zhou N, Zhang X, Zou J-J, et al. Isolation of SARS-CoV-2-related coronavirus from Malayan pangolins. Nature. 2020;583(7815):286-9.

2. Phan TG, Vo NP, Boros A, Pankovics P, Reuter G, Li OT, et al. The viruses of wild pigeon droppings. PLoS One. 2013;8(9):e72787.

3. Jones KE, Patel NG, Levy MA, Storeygard A, Balk D, Gittleman JL, et al. Global trends in emerging infectious diseases. Nature. 2008;451(7181):990-3.

4. Dong X, Soong L. Emerging and Re-emerging Zoonoses are Major and Global Challenges for Public Health. Zoonoses. 2021;1(1).

5. Dharmarajan G, Li R, Chanda E, Dean KR, Dirzo R, Jakobsen KS, et al. The Animal Origin of Major Human Infectious Diseases: What Can Past Epidemics Teach Us About Preventing the Next Pandemic? Zoonoses. 2022;2(1).

6. Wolfe ND, Dunavan CP, Diamond J. Origins of major human infectious diseases. Nature. 2007;447(7142):279-83.

7. Chan JF, To KK, Tse H, Jin DY, Yuen KY. Interspecies transmission and emergence of novel viruses: lessons from bats and birds. Trends Microbiol. 2013;21(10):544-55.

8. Chan JF, To KK, Chen H, Yuen KY. Cross-species transmission and emergence of novel viruses from birds. Curr Opin Virol. 2015;10:63-9.

9. Olsen B, Munster VJ, Wallensten A, Waldenstrom J, Osterhaus AD, Fouchier RA. Global patterns of influenza a virus in wild birds. Science. 2006;312(5772):384-8.

10. Naguib MM, Verhagen JH, Mostafa A, Wille M, Li R, Graaf A, et al. Global patterns of avian influenza A (H7): virus evolution and zoonotic threats. FEMS Microbiol Rev. 2019;43(6):608-21.

11. Murray KO, Mertens E, Despres P. West Nile virus and its emergence in the United States of America. Vet Res. 2010;41(6):67.

12. Ziegler U, Fischer D, Eiden M, Reuschel M, Rinder M, Muller K, et al. Sindbis virus- a wild bird associated zoonotic arbovirus circulates in Germany. Vet Microbiol. 2019;239:108453.

13. Papa A, Tsergouli K, Tsioka K, Mirazimi A. Crimean-Congo Hemorrhagic Fever: Tick-Host-Virus Interactions. Front Cell Infect Microbiol. 2017;7:213.

14. Krammer F, Smith GJD, Fouchier RAM, Peiris M, Kedzierska K, Doherty PC, et al. Influenza. Nat Rev Dis Primers. 2018;4(1):3.

15. Fouchier RAM, Schneeberger PM, Rozendaal FW, Broekman JM, Kemink SAG, Munster V, et al. Avian influenza A virus (H7N7) associated with human conjunctivitis and a fatal case of acute respiratory distress syndrome. Proc Natl Acad Sci U S A. 2004;101(5):1356-61.

16. Wille M, Holmes EC. Wild birds as reservoirs for diverse and abundant gamma- and deltacoronaviruses. FEMS Microbiol Rev. 2020;44(5):631-44.

17. Brown KE. The expanding range of parvoviruses which infect humans. Rev Med Virol. 2010;20(4):231-44.

18. Pénzes JJ, de Souza WM, Agbandje-McKenna M, Gifford RJ. An ancient lineage of highly divergent parvoviruses infects both vertebrate and invertebrate hosts. Viruses. 2019 Jun 6;11(6):525. doi: 10.3390/v11060525.

19. Penzes JJ, Soderlund-Venermo M, Canuti M, Eis-Hubinger AM, Hughes J, Cotmore SF, et al. Reorganizing the family Parvoviridae: a revised taxonomy independent of the canonical approach based on host association. Arch Virol. 2020;165(9):2133-46.

20. Phan TG, Vo NP, Bonkoungou IJ, Kapoor A, Barro N, O'Ryan M, et al. Acute diarrhea in West African children: diverse enteric viruses and a novel parvovirus genus. J Virol. 2012;86(20):11024-30.

21. Roediger B, Lee Q, Tikoo S, Cobbin JCA, Henderson JM, Jormakka M, et al. An Atypical Parvovirus Drives Chronic Tubulointerstitial Nephropathy and Kidney Fibrosis. Cell. 2018;175(2):530-43 e24.

22. Kapoor A, Mehta N, Dubovi EJ, Simmonds P, Govindasamy L, Medina JL, et al. Characterization of novel canine bocaviruses and their association with respiratory disease. The Journal of general virology. 2012;93(Pt 2):341-6.

23. Sharp CP, LeBreton M, Kantola K, Nana A, Diffo JLD, Djoko CF, et al. Widespread infection with homologues of human parvoviruses B19, PARV4, and human bocavirus of chimpanzees and gorillas in the wild. Journal of virology. 2010;84(19):10289-96.

24. Pham HT, Yu Q, Bergoin M, Tijssen P. A Novel Ambisense Densovirus, Acheta domesticus Mini Ambidensovirus, from Crickets. Genome Announc. 2013;1(6).

25. Vibin J, Chamings A, Klaassen M, Bhatta TR, Alexandersen S. Metagenomic characterisation of avian parvoviruses and picornaviruses from Australian wild ducks. Sci Rep. 2020;10(1):12800.

26. Wang Y, Yang S, Liu D, Zhou C, Li W, Lin Y, et al. The fecal virome of red-crowned cranes. Arch Virol. 2019;164(1):3-16.

27. Soliman MA, Erfan AM, Samy M, Mahana O, Nasef SA. Detection of Novel Goose Parvovirus Disease Associated with Short Beak and Dwarfism Syndrome in Commercial Ducks. Animals (Basel). 2020;10(10).

28. Wang J, Ling J, Wang Z, Huang Y, Zhu J, Zhu G. Molecular characterization of a novel Muscovy duck parvovirus isolate: evidence of recombination between classical MDPV and goose parvovirus strains. BMC Vet Res. 2017;13(1):327.

29. Zsak L, Strother KO, Day JM. Development of a polymerase chain reaction procedure for detection of chicken and turkey parvoviruses. Avian Dis. 2009;53(1):83-8.

30. Domanska-Blicharz K, Jacukowicz A, Lisowska A, Minta Z. Genetic characterization of parvoviruses circulating in turkey and chicken flocks in Poland. Arch Virol. 2012;157(12):2425-30.

31. Nunez LF, Santander Parra SH, Mettifogo E, Astolfi-Ferreira CS, Piantino Ferreira AJ. Isolation and molecular characterisation of chicken parvovirus from Brazilian flocks with enteric disorders. Br Poult Sci. 2015;56(1):39-47.

32. Feng B, Xie Z, Deng X, Xie L, Xie Z, Huang L, et al. Genetic and phylogenetic analysis of a novel parvovirus isolated from chickens in Guangxi, China. Arch Virol. 2016;161(11):3285-9.

33. Uttenthal A, Lund E, Hansen M. Mink enteritis parvovirus. Stability of virus kept under outdoor conditions. APMIS. 1999;107(3):353-8.

34. Chen H, Tang Y, Dou Y, Zheng X, Diao Y. Evidence for Vertical Transmission of Novel Duck-Origin Goose Parvovirus-Related Parvovirus. Transbound Emerg Dis. 2016;63(3):243-7.

35. Roux S, Hallam SJ, Woyke T, Sullivan MB. Viral dark matter and virus-host interactions resolved from publicly available microbial genomes. Elife. 2015 Jul 22;4:e08490. doi: 10.7554/eLife.08490.

36. Krishnamurthy SR, Wang D. Origins and challenges of viral dark matter. Virus Res. 2017;239:136-42.

37. Shan T, Yang S, Wang H, Wang H, Zhang J, Gong G, et al. Virome in the cloaca of wild and breeding birds revealed a diversity of significant viruses. Microbiome. 2022;10(1):60.

38. Lu X, Hua X, Wang Y, Zhang D, Jiang S, Yang S, et al. Comparison of gut viral communities in diarrhoea and healthy dairy calves. J Gen Virol. 2021;102(10).

39. Zhang W, Li L, Deng X, Kapusinszky B, Pesavento PA, Delwart E. Faecal virome of cats in an animal shelter. J Gen Virol. 2014;95(Pt 11):2553-64.

40. Deng X, Naccache SN, Ng T, Federman S, Li L, Chiu CY, et al. An ensemble strategy that significantly improves de novo assembly of microbial genomes from metagenomic next-generation sequencing data. Nucleic Acids Res. 2015;43(7):e46.

41. Skewes-Cox P, Sharpton TJ, Pollard KS, DeRisi JL. Profile hidden Markov models for the detection of viruses within metagenomic sequence data. PloS one. 2014;9(8):e105067.

42. Eddy SR. A new generation of homology search tools based on probabilistic inference. Genome Inform. 2009;23(1):205-11.

43. Finn RD, Clements J, Eddy SR. HMMER web server: interactive sequence similarity searching. Nucleic Acids Res. 2011;39(Web Server issue):W29-W37.

44. Johnson LS, Eddy SR, Portugaly E. Hidden Markov model speed heuristic and iterative HMM search procedure. BMC Bioinformatics. 2010;11:431.

45. Kearse M, Moir R, Wilson A, Stones-Havas S, Cheung M, Sturrock S, et al. Geneious Basic: an integrated and extendable desktop software platform for the organization and analysis of sequence data. Bioinformatics. 2012;28(12):1647-9.

46. Ronquist F, Teslenko M, van der Mark P, Ayres DL, Darling A, Höhna S, et al. MrBayes 3.2: efficient Bayesian phylogenetic inference and model choice across a large model space. Syst Biol. 2012;61(3):539-42.

47. Krupovic M, Koonin EV. Evolution of eukaryotic single-stranded DNA viruses of the Bidnaviridae family from genes of four other groups of widely different viruses. Sci Rep. 2014;4:5347.

48. Johnson RM, Rasgon JL. Densonucleosis viruses (‘densoviruses’) for mosquito and pathogen control. Current Opinion in Insect Science. 2018;28:90-7.

49. Mietzsch M, Penzes JJ, Agbandje-McKenna M. Twenty-Five Years of Structural Parvovirology. Viruses. 2019;11(4).

50. Yang S, Liu Z, Wang Y, Li W, Fu X, Lin Y, et al. A novel rodent Chapparvovirus in feces of wild rats. Virol J. 2016;13:133.

51. Reuter G, Boros A, Delwart E, Pankovics P. Novel circular single-stranded DNA virus from turkey faeces. Arch Virol. 2014;159(8):2161-4.

52. Penzes JJ, de Souza WM, Agbandje-McKenna M, Gifford RJ. An Ancient Lineage of Highly Divergent Parvoviruses Infects both Vertebrate and Invertebrate Hosts. Viruses. 2019;11(6).

53. Palombieri A, Di Profio F, Lanave G, Capozza P, Marsilio F, Martella V, et al. Molecular detection and characterization of Carnivore chaphamaparvovirus 1 in dogs. Vet Microbiol. 2020;251:108878.

54. Naccache SN, Greninger AL, Lee D, Coffey LL, Phan T, Rein-Weston A, et al. The perils of pathogen discovery: origin of a novel parvovirus-like hybrid genome traced to nucleic acid extraction spin columns. J Virol. 2013;87(22):11966-77.

55. Hayakawa T, Kojima K, Nonaka K, Nakagaki M, Sahara K, Asano Si, et al. Analysis of proteins encoded in the bipartite genome of a new type of parvo-like virus isolated from silkworm - structural protein with DNA polymerase motif. Virus Research. 2000;66(1):101-8.

56. Zhang J, Li G, Chen H, Li X, Lv M, Chen K, et al. Molecular cloning and expression of key gene encoding hypothetical DNA polymerase from B. mori parvo-like virus. Genet Mol Biol. 2010;33(4):739-44.

57. Krupovic M, Koonin EV. Evolution of eukaryotic single-stranded DNA viruses of the Bidnaviridae family from genes of four other groups of widely different viruses. Scientific Reports. 2014;4:5347.

58. Krupovic M, Koonin EV. Polintons: a hotbed of eukaryotic virus, transposon and plasmid evolution. Nature Reviews Microbiology. 2015;13(2):105-15.

59. Kapgate SS, Kumanan K, Vijayarani K, Barbuddhe SB. Avian parvovirus: classification, phylogeny, pathogenesis and diagnosis. Avian Pathol. 2018;47(6):536-45.

60. Kailasan S, Agbandje-McKenna M, Parrish CR. Parvovirus Family Conundrum: What Makes a Killer? Annual Review of Virology. 2015;2(1):425-50.

61. Zádori Z, Szelei J, Lacoste MC, Li Y, Gariépy S, Raymond P, et al. A viral phospholipase A2 is required for parvovirus infectivity. Dev Cell. 2001;1(2):291-302.

62. Girod A, Wobus CE, Zádori Z, Ried M, Leike K, Tijssen P, et al. The VP1 capsid protein of adeno-associated virus type 2 is carrying a phospholipase A2 domain required for virus infectivity. The Journal of General Virology. 2002;83(Pt 5):973-8.

63. Penzes JJ, Pham HT, Chipman P, Bhattacharya N, McKenna R, Agbandje-McKenna M, et al. Molecular biology and structure of a novel penaeid shrimp densovirus elucidate convergent parvoviral host capsid evolution. Proc Natl Acad Sci U S A. 2020;117(33):20211-22.

64. Liu X, Wang H, Liu X, Li Y, Chen J, Zhang J, et al. Genomic and transcriptional analyses of novel parvoviruses identified from dead peafowl. Virology. 2020;539:80-91.

65. Dai Z; Wang H; Wu H; Zhang Q; Ji L; Wang X; Shen Q; Yang S; Ma X; Shan T; Zhang W (2022): Supporting data for "Parvovirus dark matter in the cloaca of wild birds." GigaScience Database. http://dx.doi.org/10.5524/102343

# Figures and tables

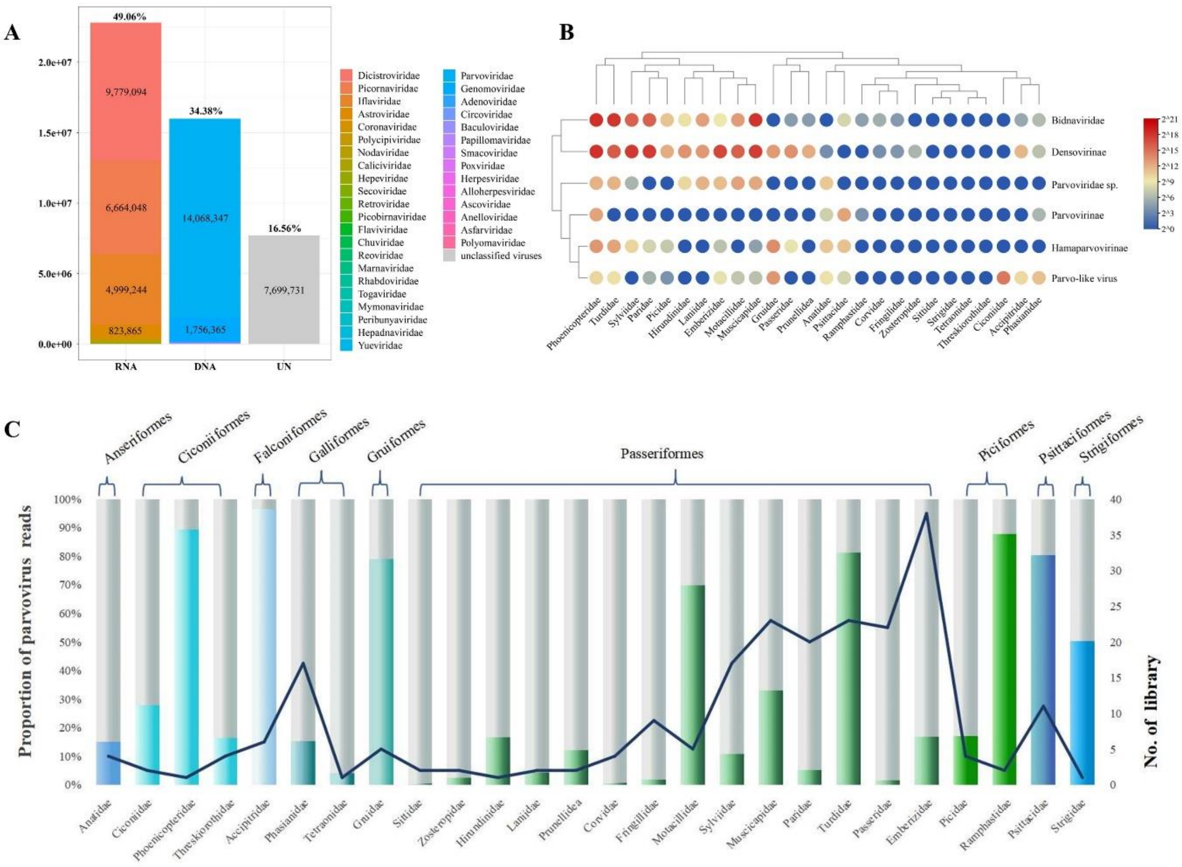


Fig 1. Overview of Virome

(A) Composition of each virus family in bird cloacal sample. (B)The shade of color in each circle shows the abundance of each group virus in each family of birds. The 170 viruses identified in this study were tentatively grouped into six groups. (C)Information of bird species

and library. Colored bars show the proportion of parvovirus reads of all viruses. The broken line shows the number of libraries for each family of birds.

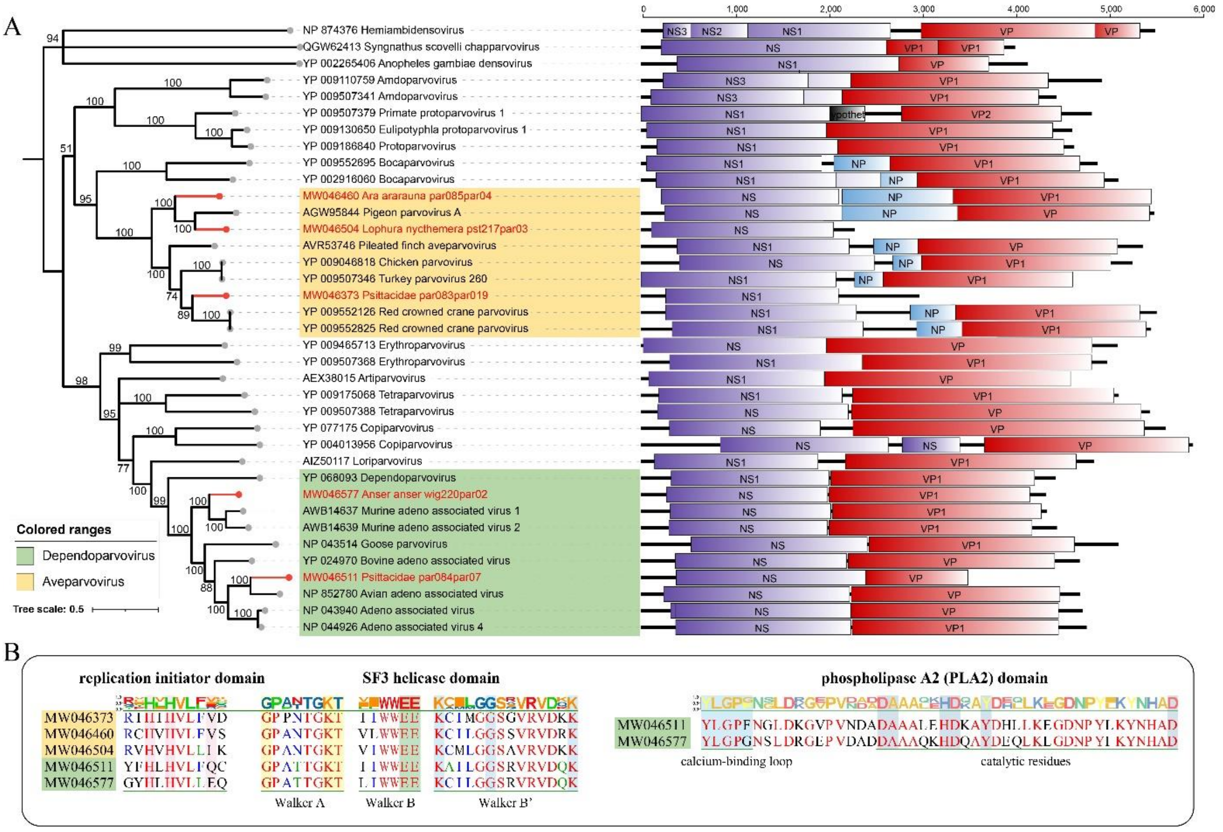


**Fig2. Identification of novel viruses of the subfamily *Parvovirinae***

(A) Bayesian inference trees were constructed using MrBayes v3.2 based on amino acid sequences of NS1 of parvovirus, within trees the viruses found in this study are labeled with red. Scale bar indicates the amino acid substitutions per site. Genome organization of each parvovirus was indicated. Purple rectangles: putative NS; red: putative VP; blue: putative NP. (B) Identification of the replication initiator domain and SF3 helicase domain in the NS1 protein and the phospholipase A2 (PLA2) domain in the N-terminal portion of the VP1 protein.

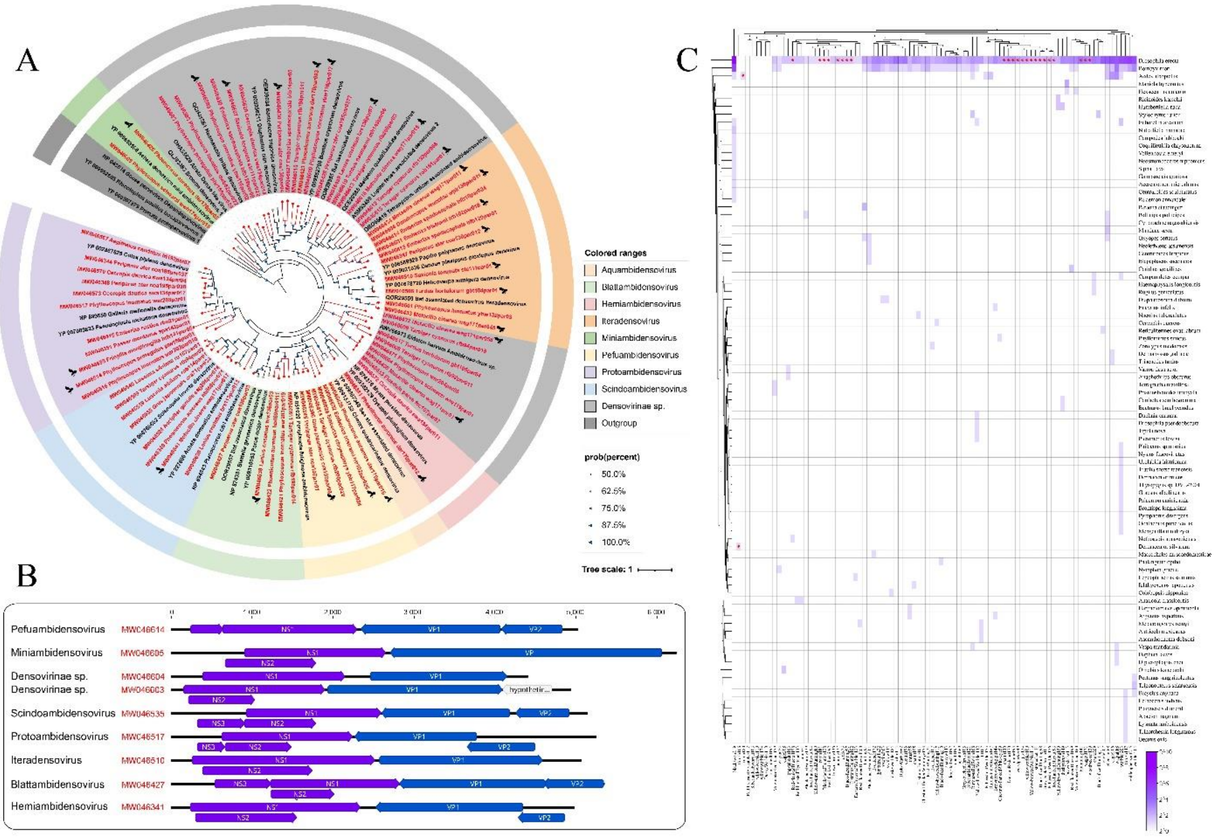


**Fig3. Identification of novel viruses of the subfamily *Densovirinae***

(A) Bayesian inference trees were constructed using MrBayes v3.2 respectively based on amino acid sequences of NS1 of densovirus, within trees the viruses found in this study are labeled with red. Scale bar indicates the amino acid substitutions per site. Potential viral hosts are shown in black silhouette. (B) Genome organization of each genus are indicated. Purple arrows and rectangles: putative NS1; blue: putative VP. (C) The composition of arthropod species in the densovirus-positive pools. The horizontal ordinate represents different pools, while the longitudinal axis represents the arthropod species. The shade of the color represents the number of the sequence reads. The library that detected only one arthropod species was marked with red star.

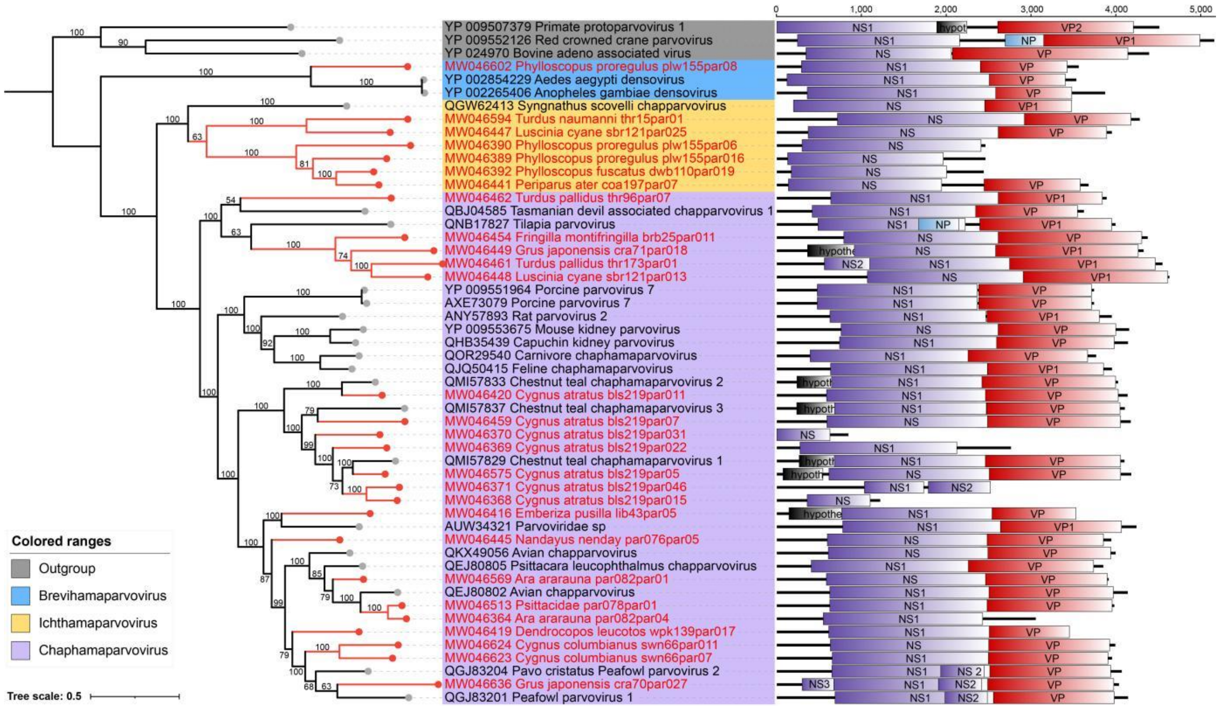


**Fig4. Identification of novel viruses of the subfamily *Hamaparvovirinae***

Bayesian inference trees were constructed using MrBayes v3.2 respectively based on amino acid sequences of NS1 of hamavirus, within trees the viruses found in this study are labeled with blue. Scale bar indicates the amino acid substitutions per site. Genome organization of hamavirus are indicated. Purple rectangles: putative NS; red: putative VP.

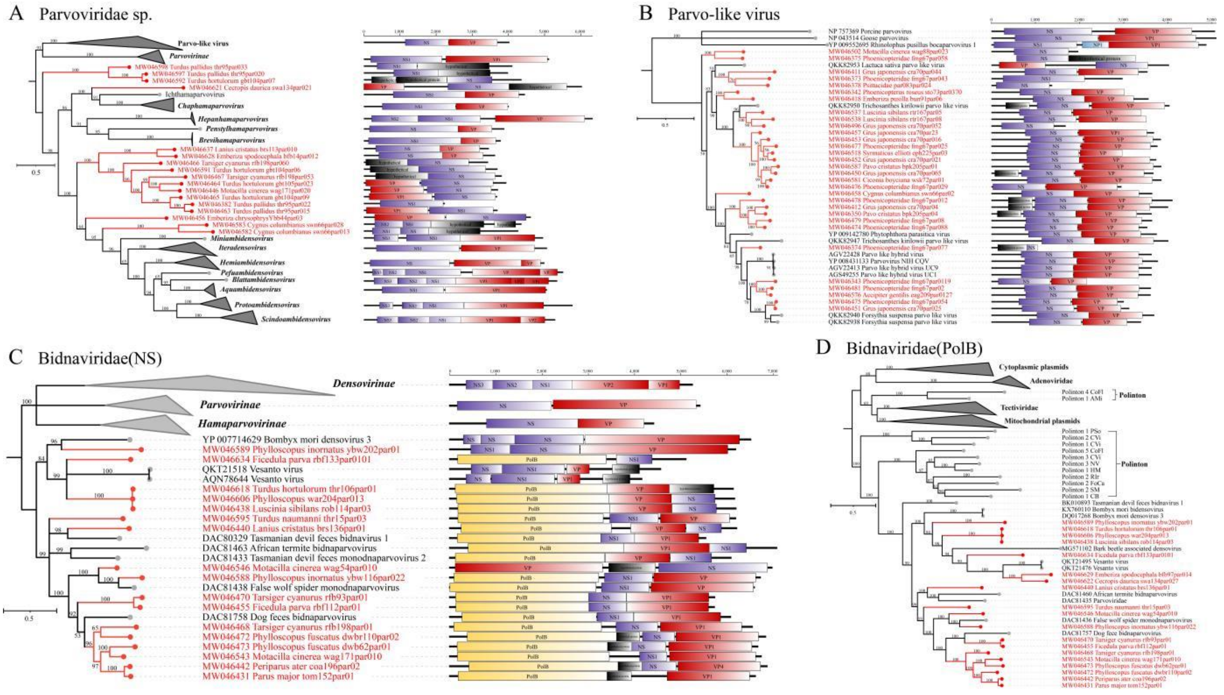


**Fig5. New viruses that may originate from parvovirus**

(A) Bayesian inference tree established based on amino acid sequences of NS1 protein of unclassified *Parvoviridae*. (B) Bayesian inference tree established based on amino acid sequences of NS1 protein of Parvo-like virus. (C) Bayesian inference tree of NS1 of *Bidnaviridae*. (D) Bayesian inference tree of family B DNA polymerases from *Bidnaviridae*, Polintons, eukaryotic linear plasmids and viruses, and bacteriophages. Within trees the viruses found in this study are labeled with red. Each scale bar indicates the amino acid substitutions persite. Purple rectangles: putative NS; red: putative VP; yellow: putative PolB.

# Supplementary Figures

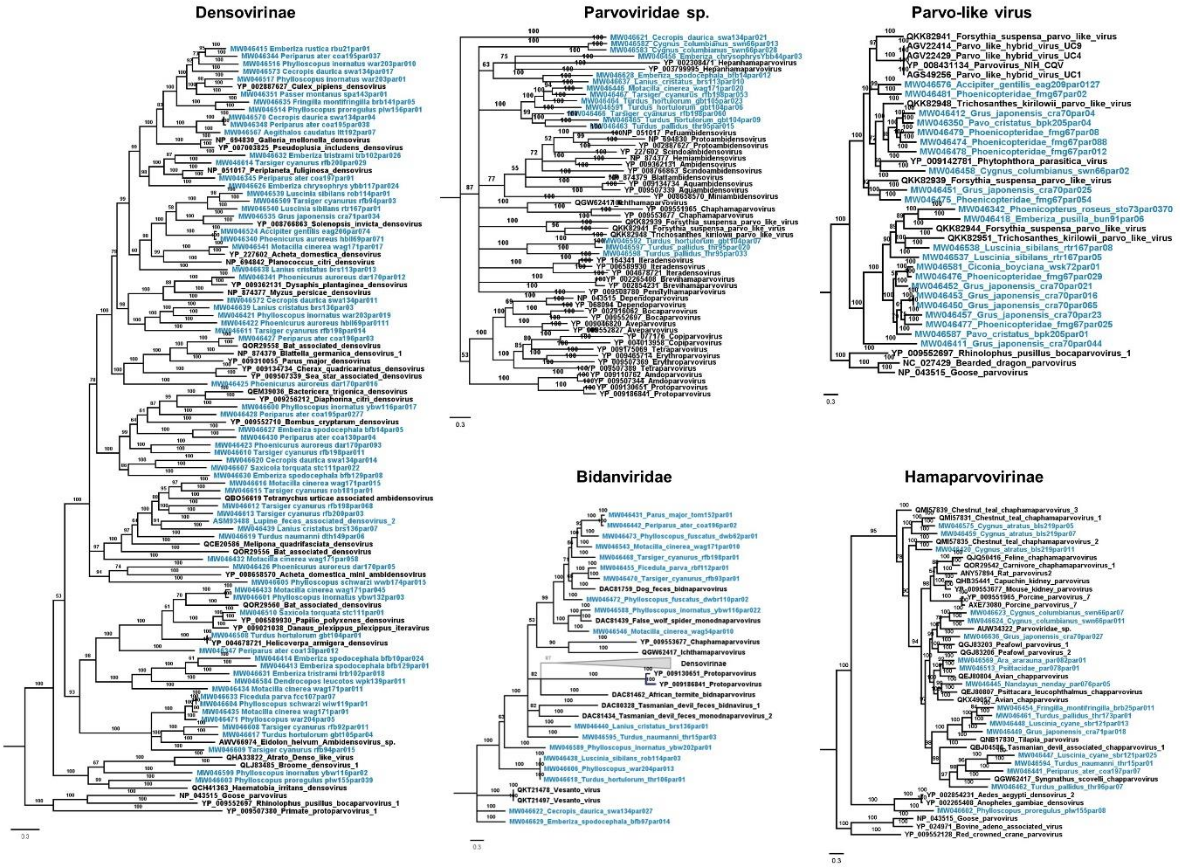


**Supplementary Fig.1 The phylogenies of VP.**

Bayesian inference tree established based on amino acid sequences of VP protein of *Densovirinae*, *Hamaparvovirinae*, Parvo-like virus, *Parvovirdae* sp. and *Bidnaviridae*. Within trees the viruses found in this study are labeled in blue. Each scale bar indicates the amino acid substitutions per site.

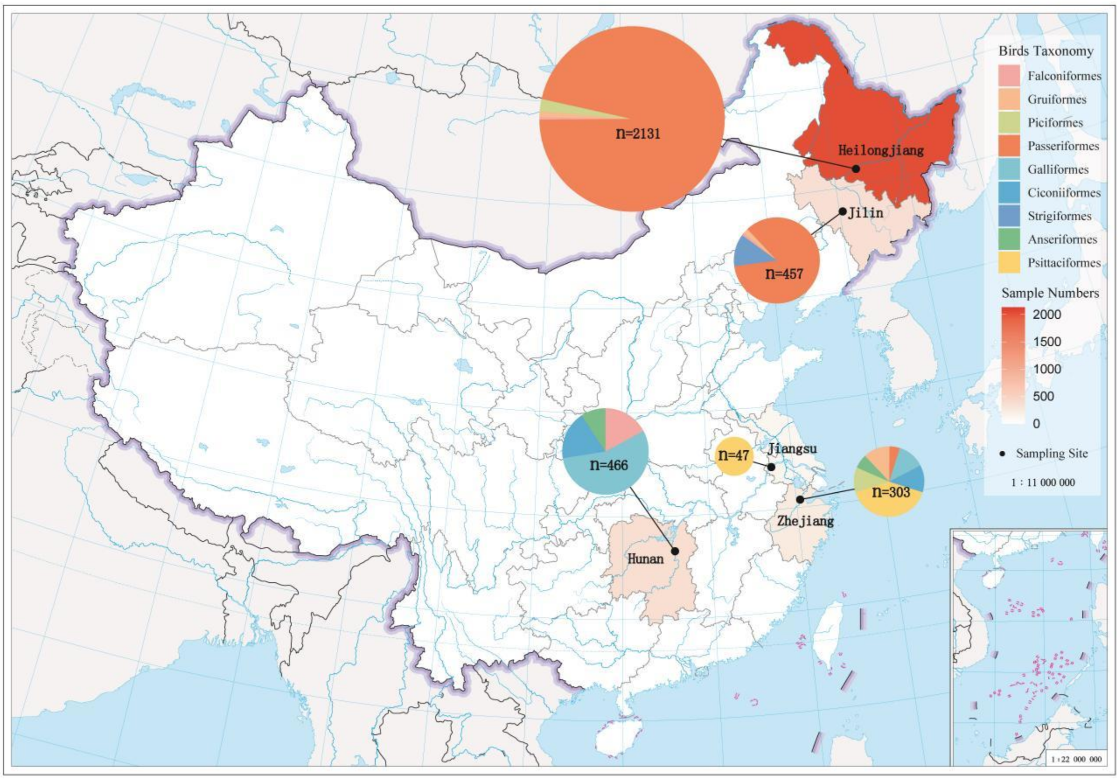


**Supplementary Fig.2 Map of sampling locations.**

The sampling sites are marked with colors. Samples were obtained from 5 provinces in China: Hunan, Zhejiang, Jiangsu, Jilin and Heilongjiang.

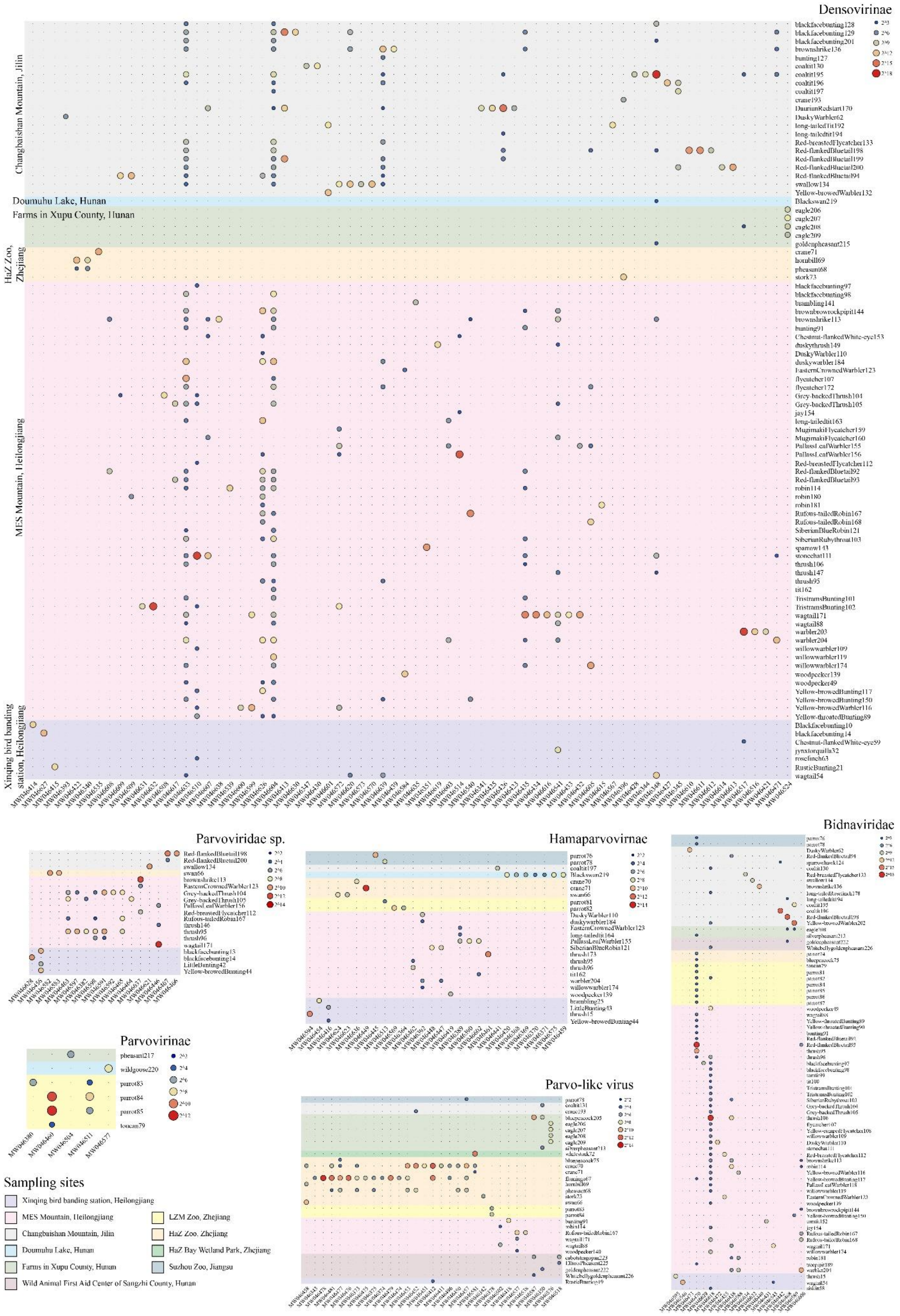
Densovirinae
Changbaishan Mountain, Jilin
Doumuhu Lake, Hunan
Farms in Xupu County, Hunan
HaZ Zoo, Zhejiang
MES Mountain, Heilongjiang
Xinqing bird banding station, Heilongjiang
Parvoviridae sp.
Hamaparvovirnae
Bidnaviridae
Parvovirinae
Parvo-like virus
Sampling sites
Xinqing bird banding station, Heilongjiang
MES Mountain, Heilongjiang
LZM Zoo, Zhejiang
Changbaishan Mountain, Jilin
HaZ Zoo, Zhejiang
Doumuhu Lake, Hunan
HuZ Bay Wetland Park, Zhejiang
Farms in Xupu County, Hunan
Suzhou Zoo, Jiangsu
Wild Animal First Aid Center of Sangzhi County, Hunan

**Supplementary Fig.3 Map of sampling locations.**

The horizontal ordinate represents different virus genomes, while the longitudinal axis represents the pools that are arranged based on birds' sampling sites. Heatmap representing the read number (in exponential form) in the mapping analysis using the genomes against NGS data of the libraries (see color legend).

**Supplementary Table 1. Information of bird species and library included in the present study.**

**Supplementary Table 2. Information of viruses identified in cloaca of birds.**

Fig 1

Click here to access/download;Figure;Fig 1 .jpg

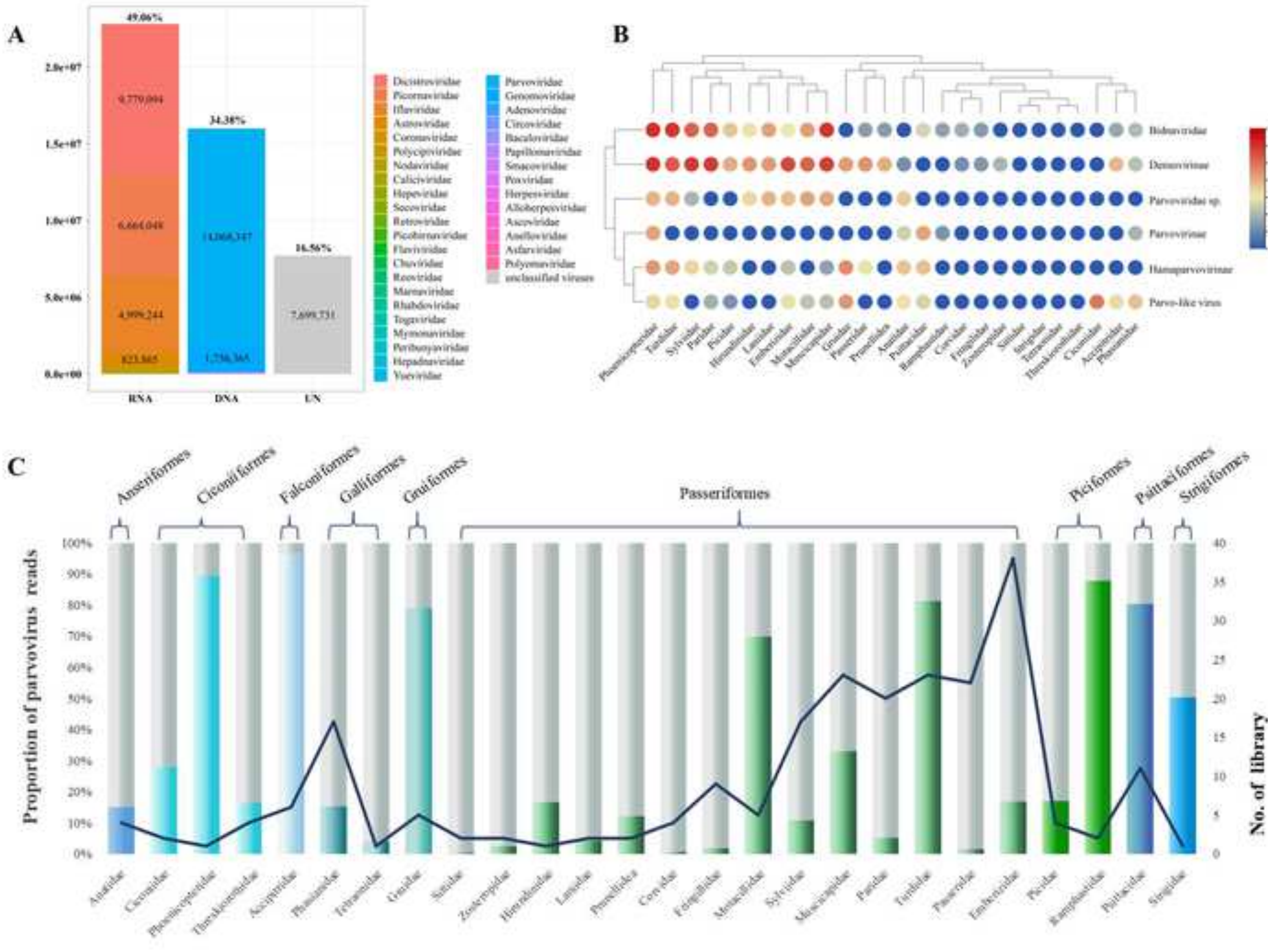

Fig 2

Click here to access/download;Figure;Fig 2 .jpg

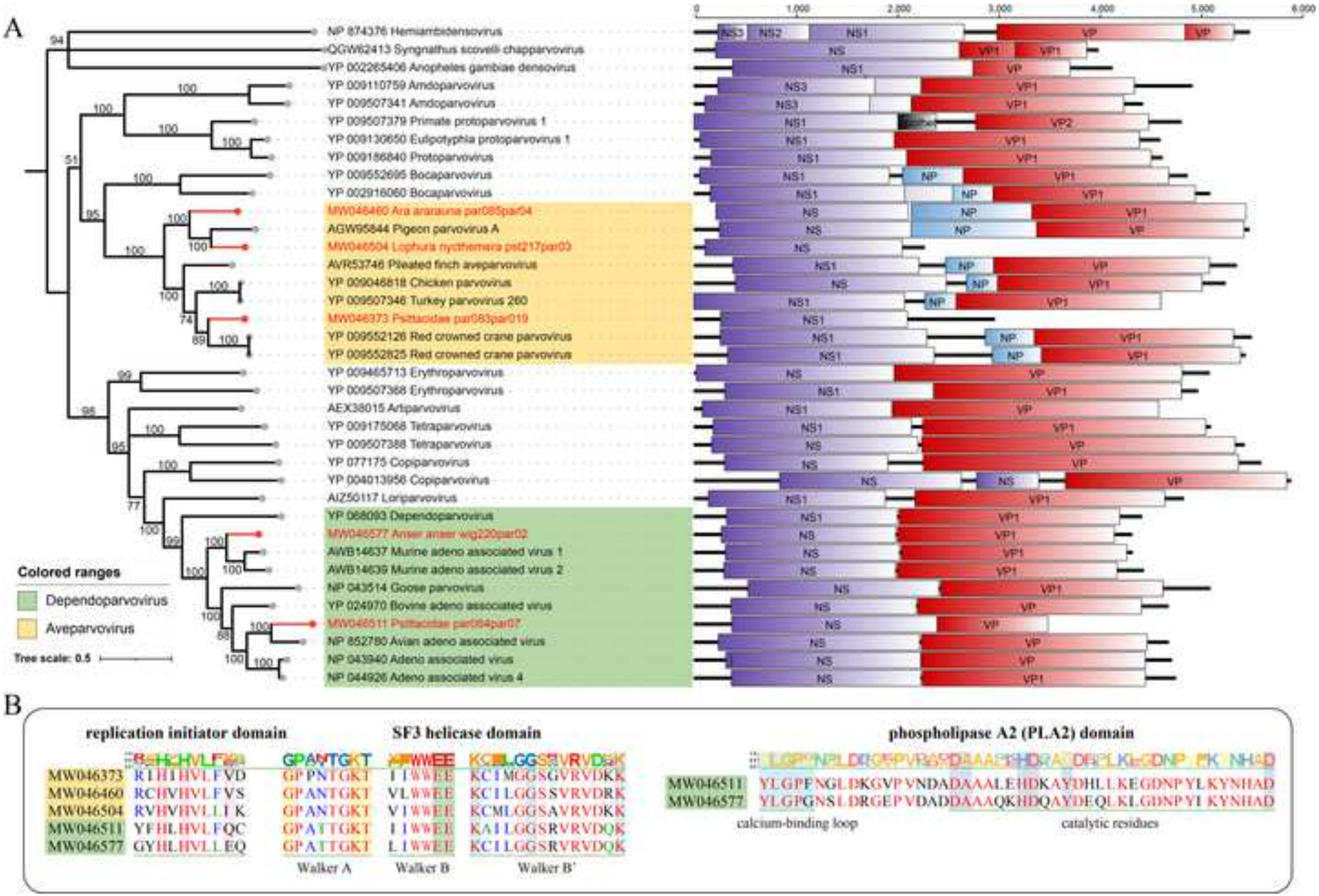

Click here to access/download;Figure;Fig 3 .jpg

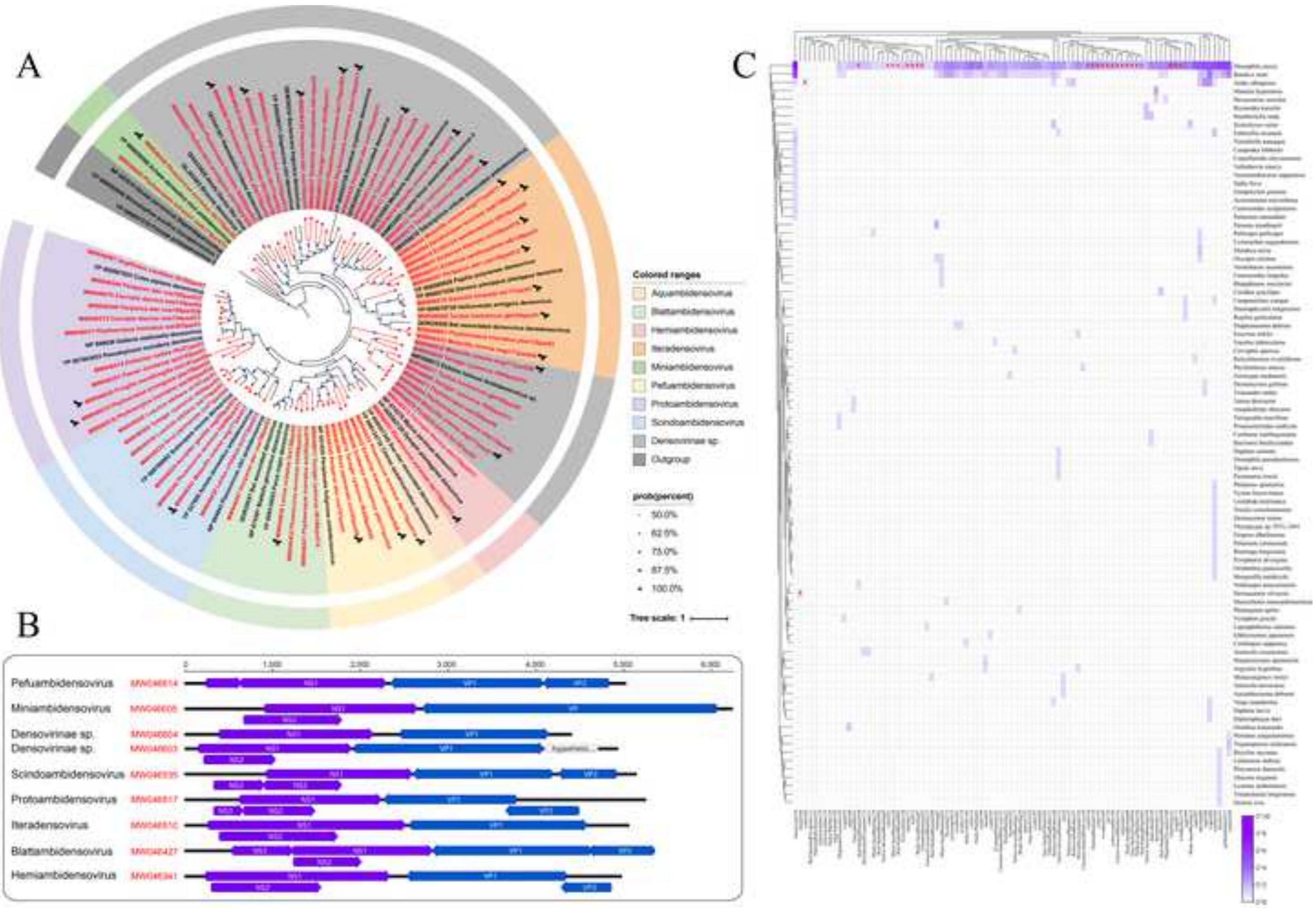

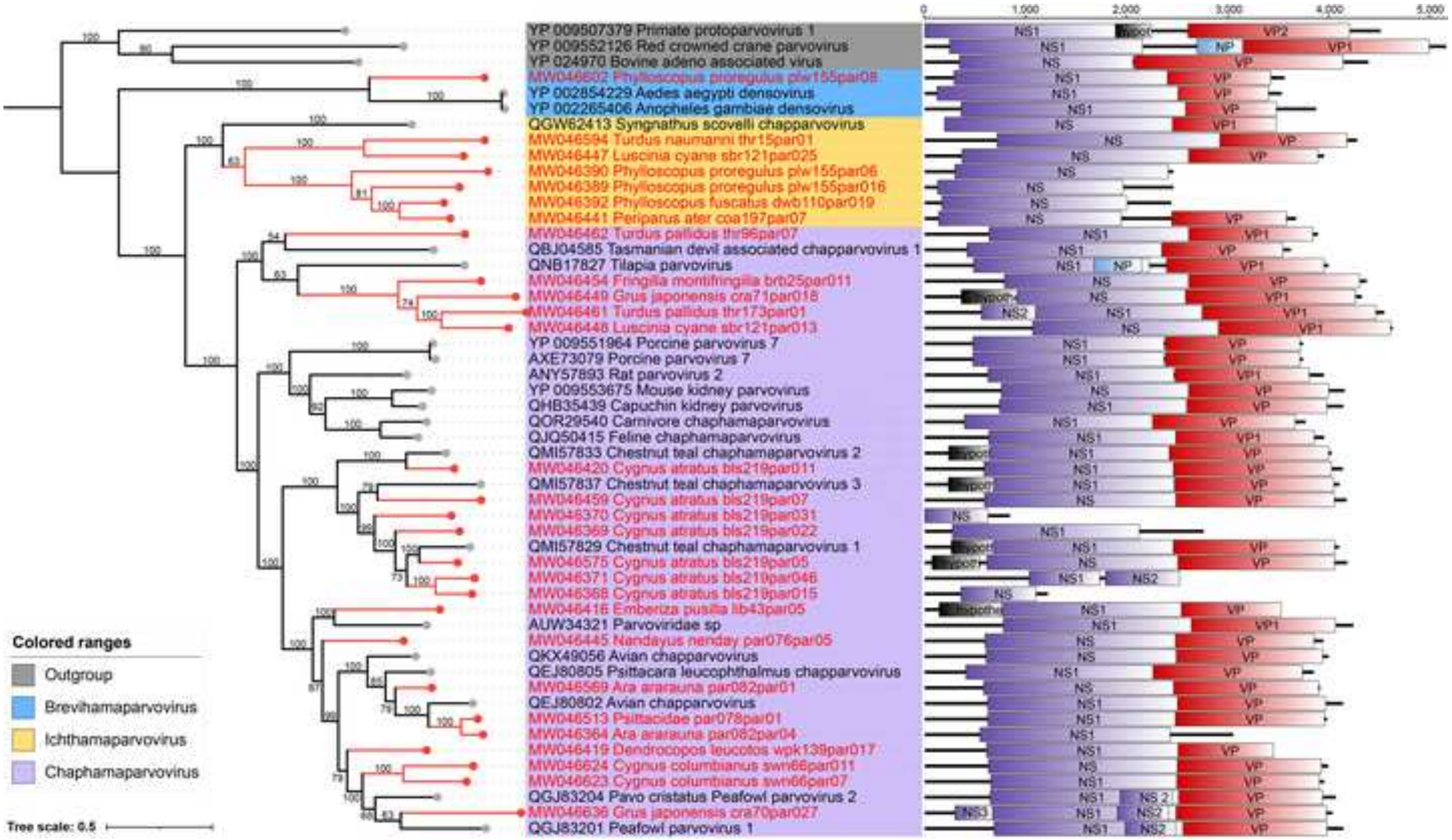
0
1,000
2,000
3,000
4,000
5,000
YP 009507379 Primate protoparvovirus 1
YP 009552126 Red crowned crane parvovirus
YP 024970 Bovine adeno associated virus
MW046602 Phylloscopus proregulus plw155par08
YP 002854229 Aedes aegypti densovirus
YP 002265406 Anopheles gambiae densovirus
QGW62413 Syngnathus scovelli chapparvovirus
MW046594 Turdus naumanni thr15par01
MW046447 Luscinia cyane sbr121par025
MW046390 Phylloscopus proregulus plw155par06
MW046389 Phylloscopus proregulus plw155par016
MW046392 Phylloscopus fuscatus dwb110par019
MW046441 Periparus ater coa197par07
MW046462 Turdus pallidus thr96par07
QBJ04585 Tasmanian devil associated chapparvovirus 1
QNB17827 Tilapia parvovirus
MW046454 Fringilla montifringilla brb25par011
MW046449 Grus japonensis cra71par018
MW046461 Turdus pallidus thr173par01
MW046448 Luscinia cyane sbr121par013
YP 009551964 Porcine parvovirus 7
AXE73079 Porcine parvovirus 7
ANY57893 Rat parvovirus 2
YP 009553675 Mouse kidney parvovirus
QHB35439 Capuchin kidney parvovirus
QOR29540 Carnivore chaphamaparvovirus
QJQ50415 Feline chaphamaparvovirus
QMI57833 Chestnut teal chaphamaparvovirus 2
MW046420 Cygnus atratus bls219par011
QMI57837 Chestnut teal chaphamaparvovirus 3
MW046459 Cygnus atratus bls219par07
MW046370 Cygnus atratus bls219par031
MW046369 Cygnus atratus bls219par022
QMI57829 Chestnut teal chaphamaparvovirus 1
MW046575 Cygnus atratus bls219par05
MW046371 Cygnus atratus bls219par046
MW046368 Cygnus atratus bls219par015
MW046416 Emberiza pusilla lib43par05
AUW34321 Parvoviridae sp
MW046445 Nandayus nenday par076par05
QKX49056 Avian chapparvovirus
QEJ80805 Psittacara leucophthalmus chapparvovirus
MW046569 Ara ararauna par082par01
QEJ80802 Avian chapparvovirus
MW046513 Psittacidae par078par01
MW046364 Ara ararauna par082par04
MW046419 Dendrocopos leucotos wpk139par017
MW046624 Cygnus columbianus swn66par011
MW046623 Cygnus columbianus swn66par07
QGJ83204 Pavo cristatus Peafowl parvovirus 2
MW046636 Grus japonensis cra70par027
QGJ83201 Peafowl parvovirus 1
Colored ranges
Outgroup
Brevihamaparvovirus
Ichthamaparvovirus
Chaphamaparvovirus
Tree scale: 0.5

Fig 5
Click here to access/download;Figure;Fig 5 .jpg

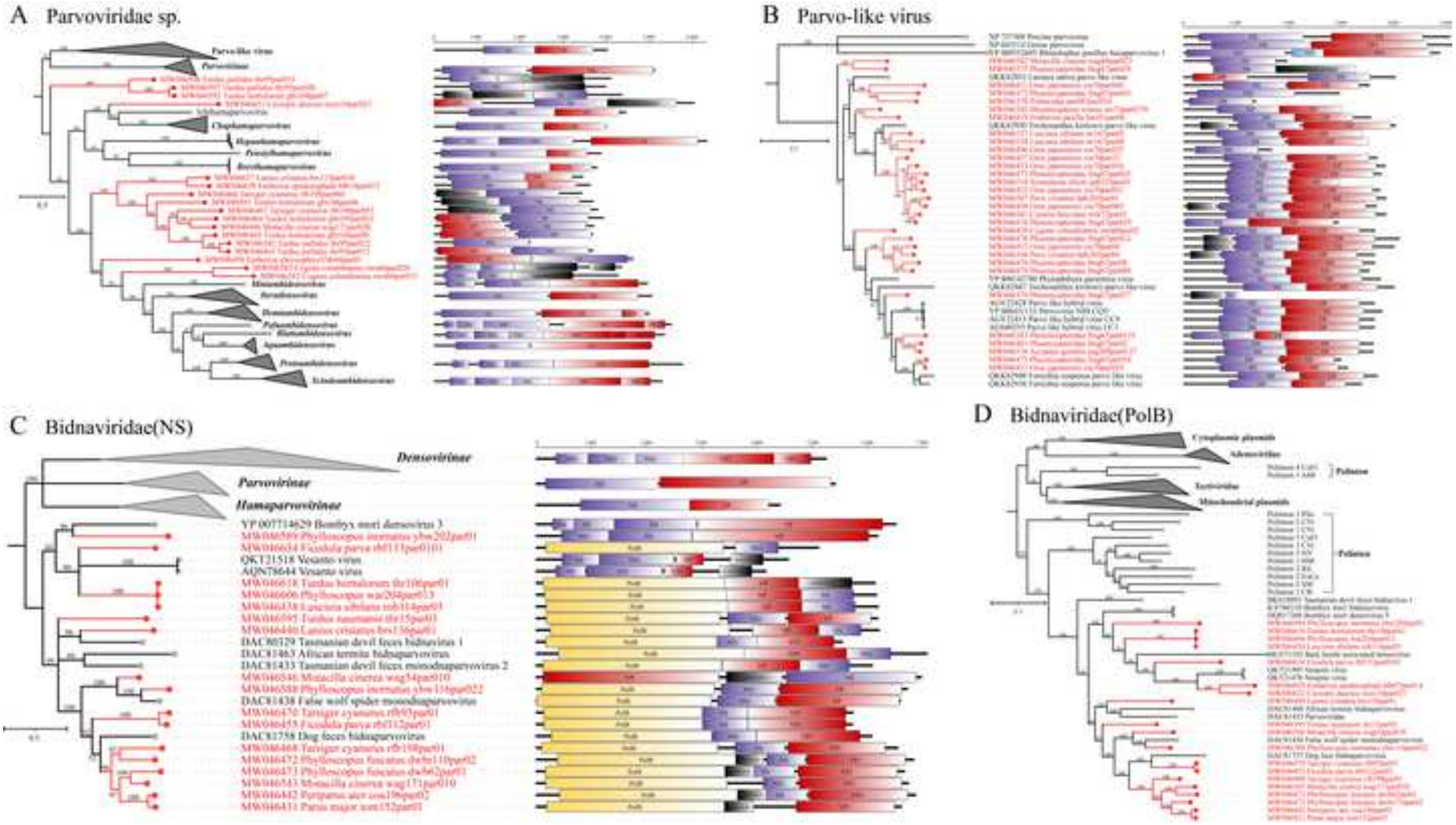

Supplementary Fig.1

Click here to access/download
**Supplementary Material**
Supplementary Fig.1 .jpg

Supplementary Fig.2

Click here to access/download
**Supplementary Material**
Supplementary Fig.2 .jpg

Supplementary Fig.3

Click here to access/download
**Supplementary Material**
Supplementary Fig.3.jpg

Supplementary_Table_1

Click here to access/download
**Supplementary Material**
Supplementary_Table_1.csv

Supplementary Table 2

Click here to access/download
**Supplementary Material**
Supplementary Table 2.xlsx
