# Supplementary figures and images for: Parvovirus dark matter in the cloaca of wild birds
Source: Gigascience. 2023 Feb 3;12:giad001. doi: 10.1093/gigascience/giad001 (PMC9896142; doi:10.1093/gigascience/giad001)

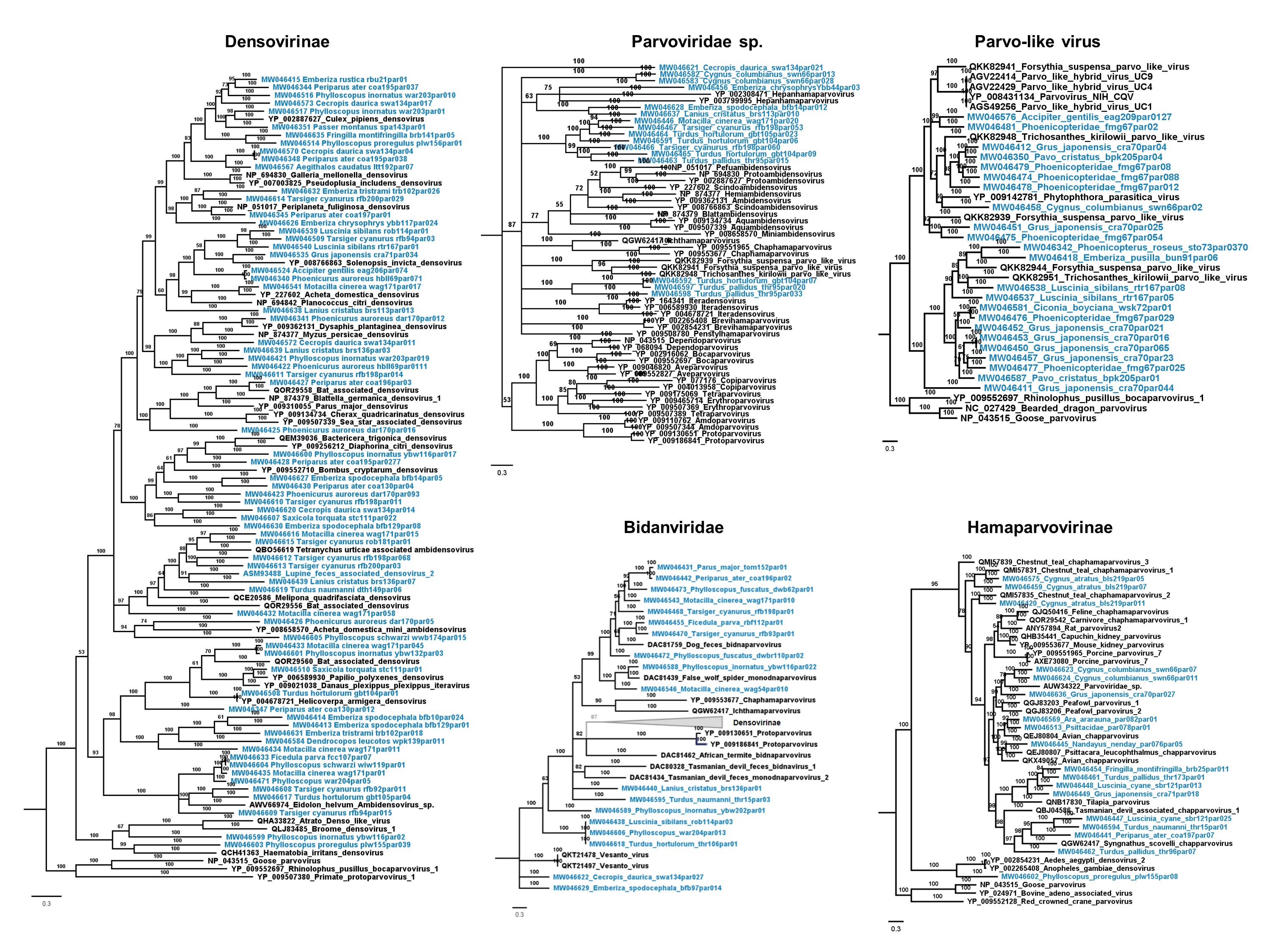

Supplement: giad001_Supplemental_Figures_and_Tables [file giad001_supplemental_figures_and_tables.zip › Supplementary Fig.1 .jpg]

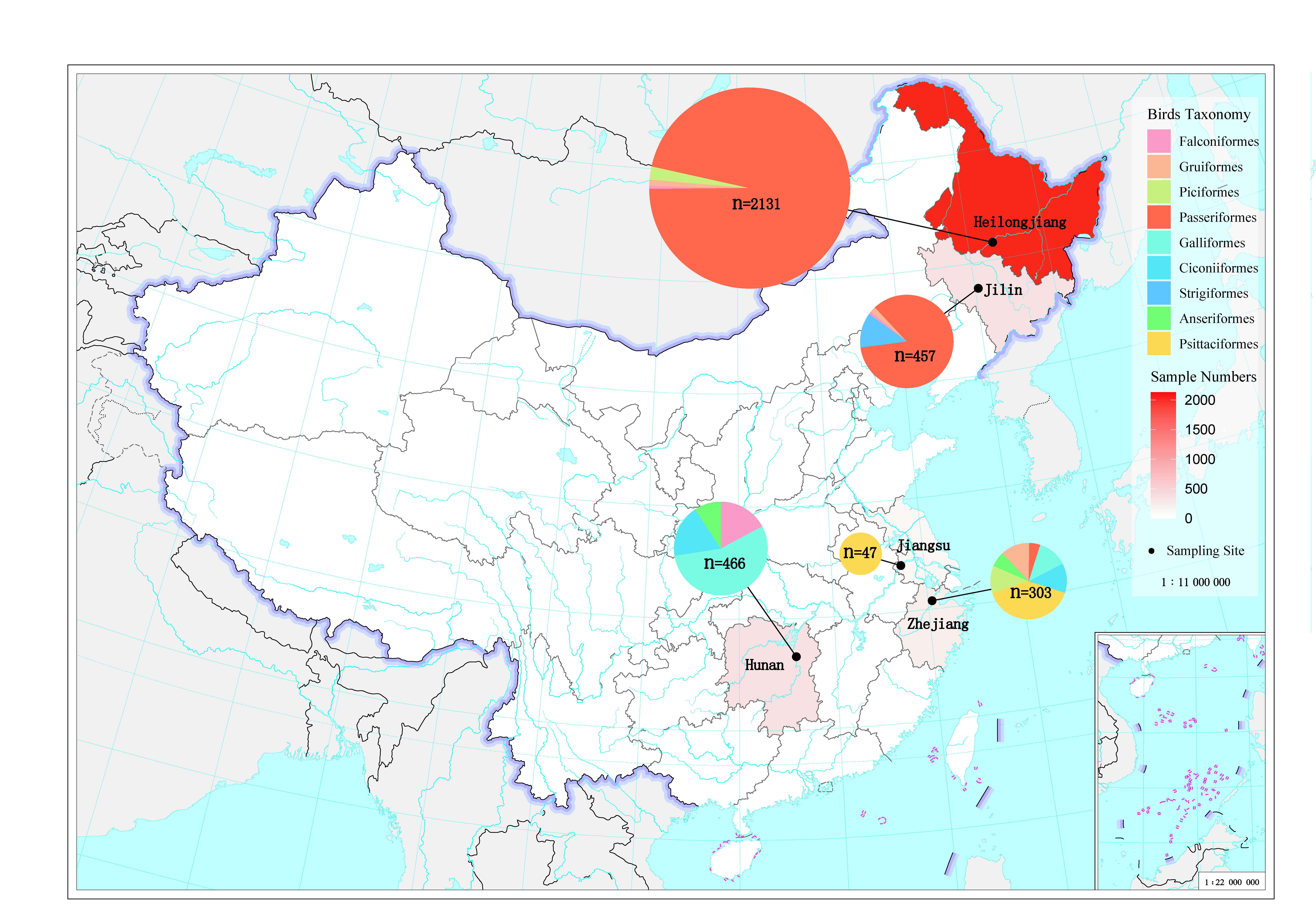

Supplement: giad001_Supplemental_Figures_and_Tables [file giad001_supplemental_figures_and_tables.zip › Supplementary Fig.2 .jpg]

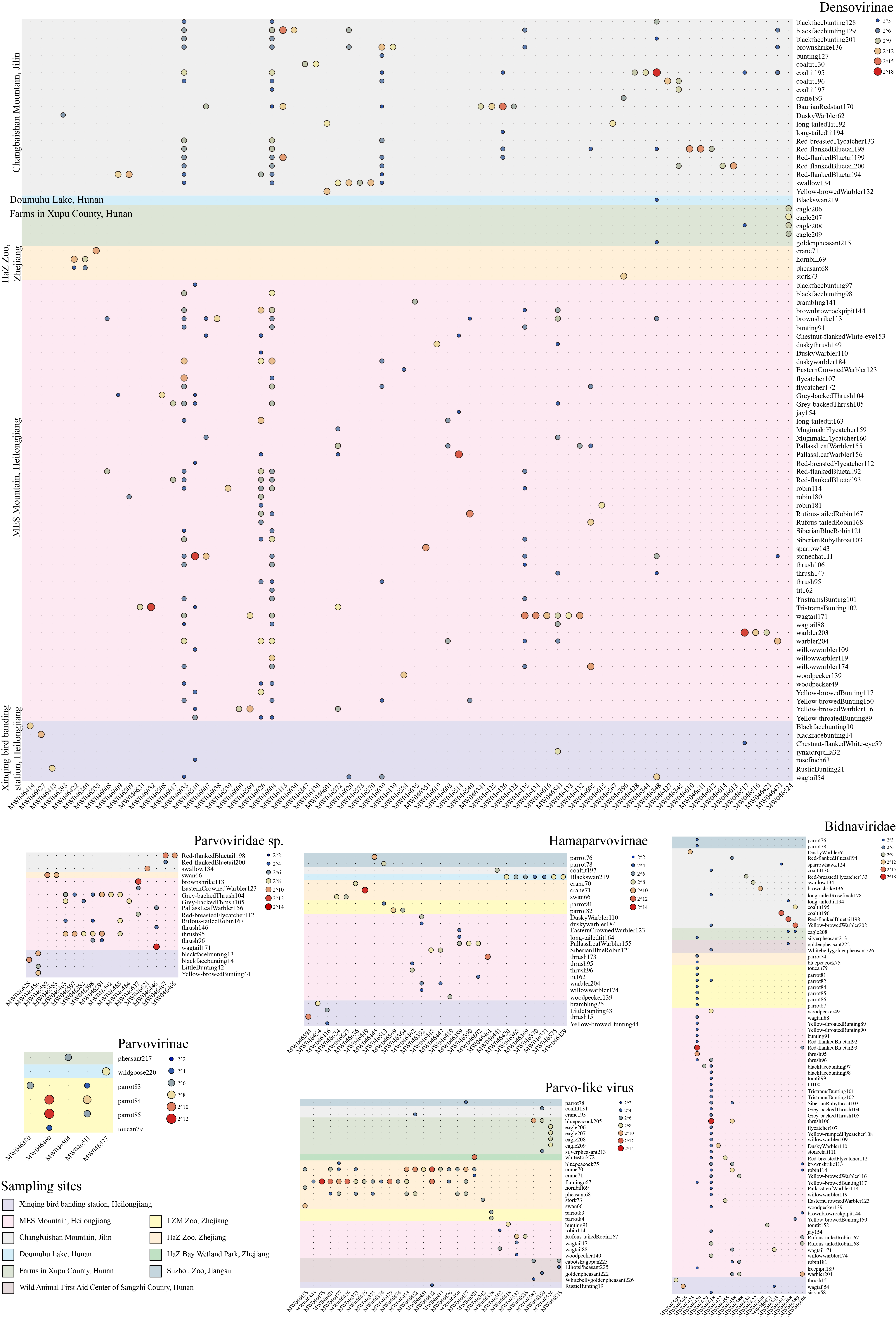

Supplement: giad001_Supplemental_Figures_and_Tables [file giad001_supplemental_figures_and_tables.zip › Supplementary Fig.3.jpg]
