# Supplementary material for: Parvovirus dark matter in the cloaca of wild birds
Source: Gigascience. 2023 Feb 3;12:giad001. doi: 10.1093/gigascience/giad001 (PMC9896142; doi:10.1093/gigascience/giad001)
Supplement: giad001_Response_to_Reviewer_Comments_Original_Submission [file giad001_response_to_reviewer_comments_original_submission.pdf]

Author's Response To Reviewer Comments

Close

Reviewer #1: In this study, Dai and colleagues investigated the diversity of viruses in the cloaca samples from a large number of birds. The authors discovered an unexpected reservoir of pavoviridae family viruses among these species including several novel viruses. The paper is very interesting, I would like to support to be published; however, there are some issues that should be addressed.

1.Densoviruses are generally hosted by invertebrates. Here, 70 novel densoviruses or denso-like viruses were discovered from the bird digestive tract samples, which are often mixed with invertebrates as the later are often birds' diet. This means the host assignments of these densoviruses are not clear. I suggest that the authors should do is use the NGS data of these densovirus-positive libraries to try to identify possible invertebrate hosts of densoviruses in the samples (i.e. the sequence reads from invertebrates in the NGS data might provide useful host information for these densoviruses). This is one of the great benefits of shotgun sequencing over enrichment protocols: the former give you host data. For doing this, the authors can compare (using BLASTx searching) the contigs assembled from the NGS data of densovirus-positive libraries (those containing possible invertebrates sequence reads) against the total mitochondrial proteome database that can be downloaded from GenBank (mitochondrial gene is a good hallmark for species classification). The searching results can be displayed as a heatmap which indicates the composition of invertebrate species present in the samples.
=Thank you for this good advice. For better host assignment of these novel desoviruses or denso-like viruses, we compared (by using BLASTx searching) the NGS data of each densovirus-positive libraries against the total mitochondrial proteome database that were downloaded from GenBank. Based on the BLASTx results, we then analyzed the composition of invertebrate species in the densovirus-positive pools based on mitochondrial genes, which is shown as a heatmap in Fig 3C, we also included the possible host information of these densovirus in Fig 3A. Several sentences describing these results are located in line 241-248 in the revised manuscript.

2.Ethical concerns: (1) No information about permission and approval concerning bird sampling (e.g. licenses from wildlife conservation and/or management offices). Some of the species listed in this study belong to protected species. (2) No information on whether the experiments were conducted under bio-safety level 2 conditions.
=Ethical approvals were given by the Ethics Committee of Key Laboratory of Wildlife Diseases and Biosecurity Management of Heilongjiang Province with the reference number of WDBM2018-023, the Ethics Committee of Jiangsu University with the reference number of 2018ujs18023 and the Ethics Committee of Chinese Academy of Agricultural Sciences with the reference number of SVRI2017091. Sample collecting was performed in accordance with the Wildlife Protection Law of the People’s Republic of China. All samples were shipped to the Shanghai Veterinary Research Institute of Chinese Academy of Agricultural Sciences where sample preparations were conducted in a biosafety level 2 laboratory. Please see lines 98-105.

3. Line 6 "identity" should be "identify"
=OK, we have corrected it. Please see line 6.
4.Lines 11-14. Redundant expressions?
=OK, we have deleted the redundant sentences.
5. Line 239 " initiated" should be "initiate"
=OK, we have corrected it. Please see line 289.

Reviewer #2: This manuscript by Dai et al. reports a large-scale metagenomic study of the fecal virome in wild birds, trying to explore the parvovirus "dark matter" to enrich known viral libraries and to explore their potential public health significance. The analyses were performed appropriately, but some issues need to

be revised to meet the publication requirements.

Introduction
Line 55. Elaborate on what is known about pathology, prevalence, etc., of parvoviruses.
=OK, We describe the pathogenicity of GPV and MDPV, and describe the prevalence and transmission of parvoviruses. Please see lines 65-74.

Line 57. The threshold for species demarcation is 85%, what is the threshold for genus demarcation?
=NS1 proteins of members of the same genus should share at least 35-40% amino acid sequence identity with a coverage of >80% between any two members. Please see lines 53-60.

Materials and methods
Line 73. Change "vigorously" to the actual speed used.
=The "vigorously" have been changed to "1,800 rpm ". Please see line 92.

Line 74. The centrifuge speed is incorrect.
=The correct centrifugal force is “15,000× g”. Please see line 93.

Line 75. "Sample collection and preparation" section does not properly explain the amount of fecal content represented in each one of the supernatants collected after preparation.
= Sample pools were added about 0.1mL supernatant of each cloacal swab specimens from the same bird species. Details of each pool can be found in Supplementary Table 1.

Line 88: Please describe the threshold that were used to define "low-sequencing-quality reads".
==Low-sequencing-quality reads, including low quality bases/reads, tag sequences, duplicates, etc. In NGS technology, the qualities of bases on most sequencing platforms will degrade as the run progresses, so it is common to see the quality of base calls falling towards the end of a read. Our research used 10 as the cutoff value to define "low-sequencing-quality reads".

Line 89. Why did the authors set the threshold to 10? Normally this value is 20.
=Our previous research used 10 as the cutoff value. Thank you for your professional reminder. We will use 20 as the cutoff value in future studies.

Line 91. why did the adapters get removed at the end of the bioinformatics pipeline and not after removing the barcodes?
=We generally remove adapters after trimming low-sequencing-quality tails, because we find that removing adapters before trimming low-sequencing-quality tails will cause more errors in de novo assembly.

Line 119. The phylogenetic analysis is missing a few important details. For instance, what does a Bayesian prior set? Were recombinant events investigated within the whole genome?
=We use the parameter “Aamodelpr” to set the rate matrix for amino acid data. Please see lines 148-150. No recombination events were found in the 170 new viral genomes identified in this study.

How to evaluate the best amino acid substitution model, and what amino acid substitution model was used to construct phylogenetic tree?
=In phylogenetic analysis, we used MrBayes to integrate over a predetermined set of fixed rate matrices and then summarize the MCMC samples and calculate the posterior probability estimate for each of these models. In our study, the particular posterior probability overwhelmingly supports the blosum model, so this maybe the only model sampled after the burn-in phase.

Results
Figure 1A, "unclassified RNA viruses-Shi M.2016" is not a proper taxa. Does it mean these RNA viruses are

highly similar to the unclassified RNA viruses reported by Shi M?
= Thank you for your professional advice. We carefully reviewed the virus taxonomic information and replotted stacked bar graphs to show the composition of individual virus families in bird cloacal sample. Please see Fig.1A.

Line 159. The conserved PLA2 motif of VP1 was found and stated within the manuscript. Were the authors able to find conserved domains in NS1? Please refer to manuscript above for guidance with this motif.
= In NS1, several conserved domains were identified, including a replication initiator domain (xxHxHxxxxx), an SF3 helicase domain with an ATP- or GTP-binding Walker A loop (GxxxxGKT), Walker B loop (xxxxEE), and Walker B' loop (KxxxxGxxxxxxxK). These conservative domains are shown in Fig.2B.

Please indicate in the manuscript whether recombination events have been found. Birds could travel long distance and are major vectors for virus spread. Did they find any highly similar parvoviruses (potential transmission) among birds from the same habitat/location, and among birds from different provinces? This could be mentioned and discussed.
=No recombination events were found in the 170 new viral genomes identified in this study. Many parvoviruses were found among different birds at MES mountain, such as MW046591, MW046463 and MW046598, which grouped as unclassified Parvoviridae. Some highly similar viruses were also found in birds from different regions, such as strain fcc107par07 (MW046633) and wiw119par01 (MW046604), both belonging to the subfamily Densovirinae, were found in birds from Jilin and Heilongjiang provinces (Supplementary Fig. 3). Future studies are needed to evaluate their potential for spillover to other species to better understand the risks to human health. Please see lines 174-184.

In the results, archaea, bacteria, and phage … were removed. What the percentage of viruses reads before remove not viruses? And what are the proportions of RNA and DNA viruses in this study?
= In total, the 228 libraries generated 483,194,686 sequence reads. Virus sequence reads accounted for 9.62%. The total number of reads in each library was added in column G of Supplementary Table 1. As shown in Figure 1A, the proportion of RNA virus reads and DNA virus reads was 49.06% and 34.38%, respectively.

Discussion
Line 252. The viruses identified in this study were from cloacal swabs, please discuss whether or not cloacal swab is associated with infection within the birds.
=Thank you for your suggestion, please see lines 320 to 325.

Close
