# Supplementary material for: Parvovirus dark matter in the cloaca of wild birds
Source: Gigascience. 2023 Feb 3;12:giad001. doi: 10.1093/gigascience/giad001 (PMC9896142; doi:10.1093/gigascience/giad001)
Supplement: giad001_Reviewer_1_Report_Original_Submission — Wei Liu -- 10/30/2022 Reviewed [file giad001_reviewer_1_report_original_submission.pdf]

Reviewer Report

Title: Parvovirus dark matter in the cloaca of wild birds

Version: Original Submission **Date:** 10/30/2022

Reviewer name: Wei Liu

Reviewer Comments to Author:

In this study, Dai and colleagues investigated the diversity of viruses in the cloaca samples from a large number of birds. The authors discovered an unexpected reservoir of pavoviridae family viruses among these species including several novel viruses. The paper is very interesting, I would like to support to be published; however, there are some issues that should be addressed.1. Densoviruses are generally hosted by invertebrates. Here, 70 novel densoviruses or denso-like viruses were discovered from the bird digestive tract samples, which are often mixed with invertebrates as the later are often birds' diet. This means the host assignments of these densoviruses are not clear. I suggest that the authors should do is use the NGS data of these densovirus-positive libraries to try to identify possible invertebrate hosts of densoviruses in the samples (i.e. the sequence reads from invertebrates in the NGS data might provide useful host information for these densoviruses). This is one of the great benefits of shotgun sequencing over enrichment protocols: the former give you host data. For doing this, the authors can compare (using BLASTx searching) the contigs assembled from the NGS data of densovirus-positive libraries (those containing possible invertebrates sequence reads) against the total mitochondrial proteome database that can be downloaded from GenBank (mitochondrial gene is a good hallmark for species classification). The searching results can be displayed as a heatmap which indicates the composition of invertebrate species present in the samples.2. Ethical concerns: (1) No information about permission and approval concerning bird sampling (e.g. licenses from wildlife conservation and/or management offices). Some of the species listed in this study belong to protected species. (2) No information on whether the experiments were conducted under bio-safety level 2 conditions.3. Line 6 "identity" should be "identify"4. Lines 11-14. Redundant expressions?5. Line 239 " initiated" should be "initiate"

Level of Interest

Please indicate how interesting you found the manuscript: Choose an item.

Quality of Written English

Please indicate the quality of language in the manuscript: Choose an item.

Declaration of Competing Interests

Please complete a declaration of competing interests, considering the following questions:

- Have you in the past five years received reimbursements, fees, funding, or salary from an organisation that may in any way gain or lose financially from the publication of this manuscript, either now or in the future?
- Do you hold any stocks or shares in an organisation that may in any way gain or lose financially from the publication of this manuscript, either now or in the future?
- Do you hold or are you currently applying for any patents relating to the content of the manuscript?
- Have you received reimbursements, fees, funding, or salary from an organization that holds or has applied for patents relating to the content of the manuscript?
- Do you have any other financial competing interests?
- Do you have any non-financial competing interests in relation to this paper?

If you can answer no to all of the above, write 'I declare that I have no competing interests' below. If your reply is yes to any, please give details below.

I declare that I have no competing interests'

I agree to the open peer review policy of the journal. I understand that my name will be included on my report to the authors and, if the manuscript is accepted for publication, my named report including any attachments I upload will be posted on the website along with the authors' responses. I agree for my report to be made available under an Open Access Creative Commons CC-BY license (http://creativecommons.org/licenses/by/4.0/). I understand that any comments which I do not wish to be included in my named report can be included as confidential comments to the editors, which will not be published.

Choose an item.

To further support our reviewers, we have joined with Publons, where you can gain additional credit to further highlight your hard work (see: https://publons.com/journal/530/gigascience). On publication of this paper, your review will be automatically added to Publons, you can then choose whether or not to claim your Publons credit. I understand this statement.

Yes Choose an item.
