# Supplementary material for: Parvovirus dark matter in the cloaca of wild birds
Source: Gigascience. 2023 Feb 3;12:giad001. doi: 10.1093/gigascience/giad001 (PMC9896142; doi:10.1093/gigascience/giad001)
Supplement: giad001_Reviewer_2_Report_Original_Submission — Yanpeng Li -- 10/31/2022 Reviewed [file giad001_reviewer_2_report_original_submission.pdf]

Reviewer Report

Title: Parvovirus dark matter in the cloaca of wild birds

Version: Original Submission **Date:** 10/31/2022

Reviewer name: Yanpeng Li

Reviewer Comments to Author:

This manuscript by Dai et al. reports a large-scale metagenomic study of the fecal virome in wild birds, trying to explore the parvovirus "dark matter" to enrich known viral libraries and to explore their potential public health significance. The analyses were performed appropriately, but some issues need to be revised to meet the publication requirements.IntroductionLine 55. Elaborate on what is known about pathology, prevalence, etc., of parvoviruses.Line 57. The threshold for species demarcation is 85%, what is the threshold for genus demarcation?Materials and methodsLine 73. Change "vigorously" to the actual speed used.Line 74. The centrifuge speed is incorrect.Line 75. "Sample collection and preparation" section does not properly explain the amount of fecal content represented in each one of the supernatants collected after preparation.Line 88: Please describe the threshold that were used to define "low-sequencing-quality reads".Line 89. Why did the authors set the threshold to 10? Normally this value is 20.Line 91. why did the adapters get removed at the end of the bioinformatics pipeline and not after removing the barcodes?Line 119. The phylogenetic analysis is missing a few important details. For instance, what does a Bayesian prior set? Were recombinant events investigated within the whole genome?The authors did not mention negative controls neither for sample preparation nor metagenomic analysis.How to evaluate the best amino acid substitution model, and what amino acid substitution model was used to construct phylogenetic tree?ResultsFigure 1A, "unclassified RNA viruses-Shi M.2016" is not a proper taxa. Does it mean these RNA viruses are highly similar to the unclassified RNA viruses reported by Shi M?Line 159. The conserved PLA2 motif of VP1 was found and stated within the manuscript. Were the authors able to find conserved domains in NS1? Please refer to manuscript above for guidance with this motif.Please indicate in the manuscript whether recombination events have been found. Birds could travel long distance and are major vectors for virus spread. Did they find any highly similar parvoviruses (potential transmission) among birds from the same habitat/location, and among birds from different provinces? This could be mentioned and discussed.In the results, archaea, bacteria, and phage … were removed. What the percentage of viruses reads before remove not viruses? And what are the proportions of RNA and DNA viruses in this study?DiscussionLine 252. The viruses identified in this study were from cloacal swabs, please discuss whether or not cloacal swab is associated with infection within the birds.

Level of Interest

Please indicate how interesting you found the manuscript: Choose an item.

Quality of Written English

Please indicate the quality of language in the manuscript: Choose an item.

Declaration of Competing Interests

Please complete a declaration of competing interests, considering the following questions:

- Have you in the past five years received reimbursements, fees, funding, or salary from an organisation that may in any way gain or lose financially from the publication of this manuscript, either now or in the future?
- Do you hold any stocks or shares in an organisation that may in any way gain or lose financially from the publication of this manuscript, either now or in the future?
- Do you hold or are you currently applying for any patents relating to the content of the manuscript?
- Have you received reimbursements, fees, funding, or salary from an organization that holds or has applied for patents relating to the content of the manuscript?
- Do you have any other financial competing interests?
- Do you have any non-financial competing interests in relation to this paper?

If you can answer no to all of the above, write 'I declare that I have no competing interests' below. If your reply is yes to any, please give details below.

I declare that I have no competing interests

I agree to the open peer review policy of the journal. I understand that my name will be included on my report to the authors and, if the manuscript is accepted for publication, my named report including any attachments I upload will be posted on the website along with the authors' responses. I agree for my report to be made available under an Open Access Creative Commons CC-BY license (http://creativecommons.org/licenses/by/4.0/). I understand that any comments which I do not wish to be included in my named report can be included as confidential comments to the editors, which will not be published.

Choose an item.

To further support our reviewers, we have joined with Publons, where you can gain additional credit to further highlight your hard work (see: https://publons.com/journal/530/gigascience). On publication of this paper, your review will be automatically added to Publons, you can then choose whether or not to claim your Publons credit. I understand this statement.

Yes Choose an item.
